# Mechanism of Plasmon-Induced Catalysis of Thiolates and the Impact of Reaction Conditions

**DOI:** 10.1021/jacs.3c09309

**Published:** 2024-01-26

**Authors:** Xiaobin Yao, Sadaf Ehtesabi, Christiane Höppener, Tanja Deckert-Gaudig, Henrik Schneidewind, Stephan Kupfer, Stefanie Gräfe, Volker Deckert

**Affiliations:** † Leibniz Institute of Photonic Technology, Albert-Einstein-Str. 9, 07745 Jena, Germany; ‡ Institute of Physical Chemistry and Abbe Center of Photonics, Friedrich Schiller University Jena, Helmholtzweg 4, 07743 Jena, Germany; § Fraunhofer Institute of Applied Optics and Precision Engineering, Albert-Einstein-Str. 7, 07745 Jena, Germany

## Abstract

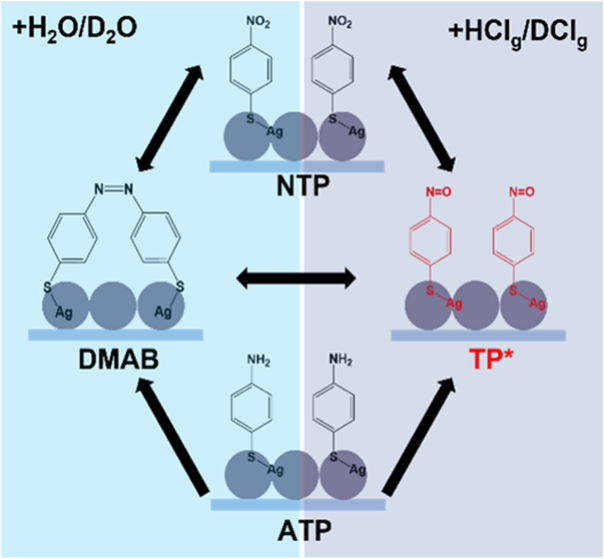

The conversion of the thiols 4-aminothiophenol (ATP) and 4-nitrothiophenol (NTP) can be considered as one of the standard reactions of plasmon-induced catalysis and thus has already been the subject of numerous studies. Currently, two reaction pathways are discussed: one describes a dimerization of the starting material yielding 4,4′-dimercaptoazobenzene (DMAB), while in the second pathway, it is proposed that NTP is reduced to ATP in HCl solution. In this combined experimental and theoretical study, we disentangled the involved plasmon-mediated reaction mechanisms by carefully controlling the reaction conditions in acidic solutions and vapor. Motivated by the different surface-enhanced Raman scattering (SERS) spectra of NTP/ATP samples and band shifts in acidic solution, which are generally attributed to water, additional experiments under pure gaseous conditions were performed. Under such acidic vapor conditions, the Raman data strongly suggest the formation of a hitherto not experimentally identified stable compound. Computational modeling of the plasmonic hybrid systems, i.e., regarding the wavelength-dependent character of the involved electronic transitions of the detected key intermediates in both reaction pathways, confirmed the experimental finding of the new compound, namely, 4-nitrosothiophenol (TP*). Tracking the reaction dynamics via time-dependent SERS measurements allowed us to establish the link between the dimer- and monomer-based pathways and to suggest possible reaction routes under different environmental conditions. Thereby, insight at the molecular level was provided with respect to the thermodynamics of the underlying reaction mechanism, complementing the spectroscopic results.

## Introduction

**Scheme 1:**
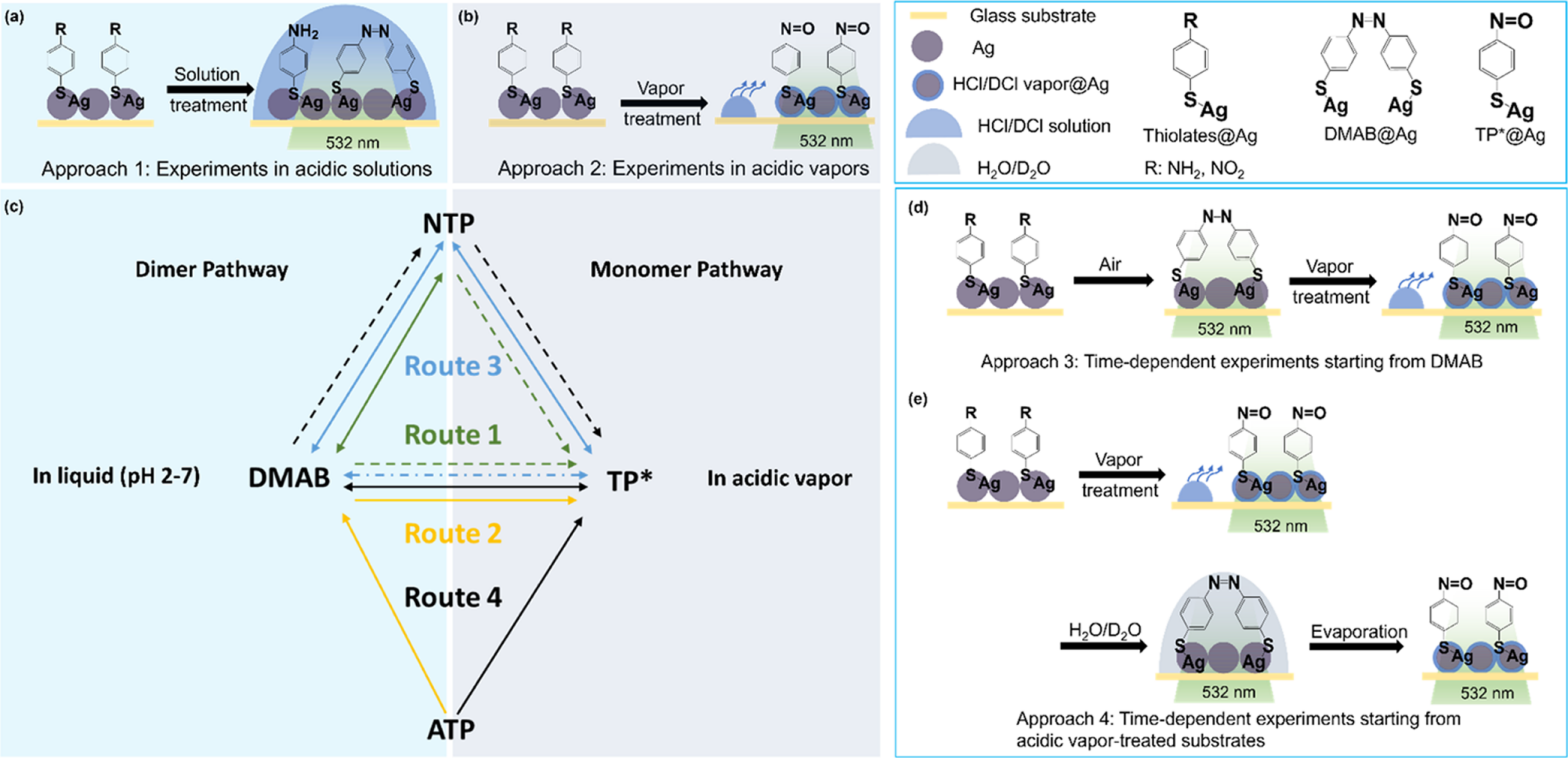
Sketch of Different Experimental Approaches Involved in This Study (a) Approach 1: Experiments in acidic solution. Measurements were taken at the spots with acidic solution. (b) Approach 2: Experiments in acidic vapor. Acidic solutions were dropped at the corners of substrates. Color change indicated the readiness for measurements. (c) Sketch of the plasmon-induced reactions of ATP and NTP on Ag-island SERS substrates. Depending on the experimental conditions, different routes are being followed. Single arrows indicate irreversible reactions, and double arrows indicate reversible reactions. Dashed lines indicate the possibility of unfavored back-reactions. Each route is labeled with corresponding colors. (d) Approach 3: Time-dependent experiments starting from DMAB. With irradiation of the laser, thiolates were converted into DMAB in air. The substrates were then treated by acidic vapor. (e) Approach 4: Time-dependent experiments starting on acidic-vapor-treated substrates. Substrates were treated with acidic vapor prior to measurements. After that, H_2_O/D_2_O was dropped onto the substrates to monitor the reactions. Measurements were continued until the water drops evaporated.

Thiols like 4-aminothiophenol (ATP) and 4-nitrothiophenol (NTP) are widely used as model systems for plasmon-induced catalysis in surface-enhanced Raman scattering (SERS).^1−9^ Self-assembled monolayers (SAMs) of these molecules on gold (Au) and silver (Ag) nanoparticle substrates can be easily prepared via Au/Ag–S bonds.^10^ Under the commonly applied illumination conditions of SERS, i.e., when matching the plasmon resonance for near-spherical Ag or Au nanoparticles around 532 nm irradiation, the adsorbed thiols efficiently dimerize to 4,4′-dimercaptoazobenzene (DMAB).^11−13^ The molecular structures of the thiolates are sketched in Scheme 1, and the Raman band positions are given in Table 1. As in the standard Raman spectra, DMAB can be clearly distinguished from the starting materials in the SERS spectra. This reaction is proposed to be induced by plasmon-mediated catalysis,^14^ as during plasmon decay, hot carriers including electrons and holes are generated which trigger chemical reactions.^15^ The multiple reactions observed in the SERS experiments of ATP and NTP stimulated an intense discussion of the involved reaction pathways. Currently, two major pathways are considered: first, the formation of DMAB, which is defined as the dimer pathway,^5,6^ and second, the reduction of NTP to ATP, which is usually produced in the presence of hydrochloric acid solution (HCl_aq_) and is referred to as the monomer pathway.^16^ It has been reported that the conversion of ATP and NTP to DMAB depends on the laser wavelength, power, and/or illumination time.^17,18^ We found that DMAB dominates the spectra even under acidic conditions between pH 2 and pH 7, which is consistent with a previous study by Qiu et al.^19^ This situation is sketched in the “liquid” approach in Scheme 1c. Only when the pH value of HCl_aq_ reaches 0 (i.e., 1 mol/L HCl), the appearance of the NO_2_ band indicates a reaction change from the dimer to the monomer pathway. It has been reported that a Raman band at ca. 1580–1590 cm^–1^ (aromatic ring stretching) can be detected when NTP-SAMs on Ag colloid substrates are treated with Cl^–^-containing acidic solutions such as HCl_aq_, which was also found in our experiments (Table 1).^16^ This band is usually considered as an indication for the reduction of NTP to ATP and is assigned to the ATP’s ring stretching mode. However, Zhang et al. reported that a direct reduction of NTP to ATP is difficult to detect without additional reducing agents.^20^ Unanticipatedly, similar phenomena on band shifts of the ring stretching mode were observed when oxidizing acids, *e.g.*, HNO_3_ and H_2_SO_4_, were used.^21^ Consequently, considering only the shift of the ring stretching mode for the formation of ATP is not reliable. Additionally, even though there is a tight connection between NTP and ATP via DMAB in the dimer pathway, direct experimental evidence of intermediates in the monomer pathway and the connection between the dimer and the monomer pathway is still missing.

**Table 1:** Band Positions and Assignments of Thiolates in Conventional Raman and SERS Spectroscopy, Summarized from Experiments of This Study^a^

		wavenumber (cm^–1^)				
		conventional Raman	SERS (@Ag film substrate)			
					acidic vapor^e^	
sample	assignments	solid^b^	air^c^	HCl_aq_ (pH 0)^d^	HCl_g_	DCl_g_
NTP	Region 1: aromatic ring breathing mode/C–S stretching	1095	1072	1079	1077	1080
	Region 2	1326 (NO_2_)	1332 (NO_2_) 1372 (N=O) 1386/1435 (Azo group)	1330 (NO_2_)	1330 (NO_2_) 1360 (N=O)	1330 (NO_2_) 1356 (N=O)
	Region 3: aromatic ring stretching	1568	1566	1561 1585 (ATP?)	1580	1577
ATP	Region 1: aromatic ring breathing mode/C–S stretching	1082	1071	1077	1077	1080
	Region 2		1362 (N=O) 1383/1430 (Azo group)	1329 (NO_2_?) 1364 (N=O)	1352 (N=O)	1353 (N=O)
	Region 3: aromatic ring stretching	1585	1570	1571	1573	1573

a Based on the spectra, the main bands are categorized into 3 regions: Region 1: ca. 1070–1100 cm^–1^ (aromatic ring breathing mode/C–S stretching), Region 2: ca. 1320–1450 cm^–1^ (functional group), and Region 3: ca. 1560–1590 cm^–1^ (aromatic ring stretching mode). All band parameters were determined via Gauss fitting.

b Data from Figure S1a,b.

c Data from Figure S1c,d.

d Data from Figure S5.

e Data from Figure 2.

In this study, joint experimental-theoretical investigations on the monomer pathway and, generally, the potential connection of the two reaction pathways were carried out. It is important to note that ligands,^22,23^ various ions,^24,25^ and even molecular orientations in single-molecule reactions^26,27^ may influence the process and can lead to distinct results. In order to avoid the interference of additional agents on nanoparticles, SERS substrates were prepared by physical vapor deposition (PVD) on precleaned glass slides. To explore the influence of environmental conditions, the SAMs on the respective substrates were investigated in both acidic solutions and acidic vapor. The different conditions are outlined in Scheme 1, which also relates such conditions to the observed reaction steps. Approach 1 displayed in Scheme 1a refers to the conditions used for the investigation of thiolates in plasmon-induced catalysis. To investigate the influence of water, we developed a facile acidic vapor method in Approach 2 (Scheme 1b). Under acidic vapors, a new compound is found in the monomer pathway for the first time, manifesting itself by the appearance of a Raman band at ∼1360 cm^–1^. These experimental results were complemented by quantum chemical calculations of the surface-immobilized intermediates to assess the thermodynamic quantities of the underlying reaction mechanism. These simulations highlight and corroborate the experimental observation of the new compound under an acidic vapor. To establish a connection between both pathways, we investigated the four different reaction routes, which comprise both the monomer and dimer pathway by tracing the associated intermediates spectroscopically through time-dependent experiments (Approaches 3 and 4, Scheme 1d,e). The results from these approaches are outlined in Scheme 1c.

## Experimental Section

ATP ≥ 97% and NTP ≥ 80% were used without further purification (Sigma-Aldrich). Deuterochloric acid (DCl_aq_) and D_2_O (Sigma-Aldrich) with concentrations of 35 wt % in D_2_O and 99.9 atom % D, respectively, were used. Hydrochloride acid solution (HCl_aq_, 37%, Sigma-Aldrich) was used for the vapor experiments and for dilution. Ethanol (>96%) was purchased from Carl Roth. 10^–7^ to 1 mol/L DCl_aq_ and HCl_aq_ were prepared from stock solutions by dilution with deionized water.

Conventional Raman spectra of ATP and NTP were acquired from the neat solid compounds using 532 nm excitation, with the laser power and the acquisition times for ATP and NTP of ∼28 mW, 1 s (power density: ∼1.4 × 10^6^ W/cm^2^) and ∼3.7 mW, 0.5 s (power density: ∼1.8 × 10^5^ W/cm^2^), respectively, at 100 accumulations (see Supporting Information, Figure S1). Conventional Raman spectra were background-corrected by subtracting the reference spectra of cleaned glass slides. The silicon signal at ∼519.4 cm^–1^ of the AFM tip was used as an internal standard.

SERS substrates were prepared by physical vapor deposition (PVD) of silver on precleaned 18 × 18 mm^2^ coverslips.^28,29^ Since Ag particles detach quickly from the substrate in liquids, the glass slides were first sputter-coated with 3 nm chromium, and subsequently, a 6 nm thick silver layer was evaporated. Annealing at 290 °C for 60 s under argon followed. This way, silver island films were formed containing densely distributed Ag particles. 1 mmol/L ATP and 1 mmol/L NTP ethanolic solutions were freshly prepared. Ag-SERS substrates were immersed in the respective solutions for 1–2 h to obtain SAMs of the thiols. The samples were thoroughly rinsed with ethanol and air-dried before the investigations. Experiments in Approach 1 were carried out in HCl_aq_ or DCl_aq_, respectively, by dropping 0.5–1 μL of 1 mol/L solution directly onto the SERS substrates. Reference experiments for the concentration dependence of NTP-SAMs on SERS substrates using HCl_aq_ and DCl_aq_ are summarized in the Supporting Information (Figure S2). In Approach 2, SERS substrates were treated with HCl/DCl vapor (HCl_g_/DCl_g_). Here, 0.5–2 μL of 1 mol/L solution was dropped onto the corners of the SERS substrates (far from the laser spot/probed area) for a few seconds to minutes to get a vapor-treated surface. For time-dependent experiments in Approach 3, DMAB was obtained by irradiating the NTP- and ATP-SAMs on the SERS substrates which were subsequently treated with acidic vapor. For the time-dependent experiments in Approach 4, TP* was directly obtained by the same treatment of NTP- and ATP-SAMs on SERS substrates. After that, ∼0.5 μL of H_2_O/D_2_O was dropped right onto the sample at the laser spot to further monitor the reaction. During the entire time-dependent experiment, the SERS substrate was constantly illuminated. Once the SERS substrates were treated with acidic vapor, a color change of the substrate from purple to brown was observed. UV–vis spectra (Varian Cary 5000) of thiolate SAMs-coated SERS substrates show that DCl_g_ treatments caused blue shifts of ∼20 nm (details see Supporting Information, Figure S3). Topography maps of the silver island films were obtained using an atomic force microscope (AFM, JPK-Bruker Nanowizard ULTRA Speed) with Budget Sensors Tap190Al-G cantilevers before and after treatment with DCl_g_ (details see Supporting Information, Figure S4). Based on the AFM images in Figure S4, it was observed that DCl affected the substrates and caused a spectral shift in the UV–vis spectra. This was probably also the reason for the observed decreasing signal-to-noise ratio during long-term measurements.

All SERS experiments were performed using a Raman microscope setup equipped with an inverted microscope (IX 71, Olympus, Japan), spectrometer (Acton Advanced, Teledyne Princeton Instruments), and CCD camera (PIXIS 256, Teledyne Princeton Instruments), 532 nm laser (Cobolt Samba, Hübner Photonics, Germany) and a 20x (N.A. 0.40) objective (LCPlanFl, Olympus, Japan).^30^ The acquisition time in single and time-dependent measurements was 1 and 0.5 s, respectively. The number of accumulations for each spectrum was between 1 and 100 and is provided with the specific experiment. A laser power of ∼280 μW was used on the SERS samples (power density: ∼1.4 × 10^4^ W/cm^2^). Except for spike removal, no further data treatment was applied to the SERS spectra. OriginPro 2020b was used for peak finding and Gauss fitting. The respective fitting conditions can be found in the Supporting Information.

Supplementary experimental data including Raman spectra of ATP and NTP, SERS spectra of ATP- and NTP-SAMs on Ag SERS substrates, AFM images, and UV–vis spectra of substrates are provided in the Supporting Information (Figures S1–S18, Table S1 and Schemes S1–S2). Supplementary modeling data including CDDs images and UV–vis spectra are provided in Supporting Information (Figures S19–S25, Tables S2–S8).

## Results and Discussion

### Reactions of Thiolates in Acidic Solutions

When comparing SERS spectra of NTP- and ATP-SAMs in the presence of HCl_aq_, a new broad band at ∼1364 cm^–1^ (ca. 1360–1370 cm^–1^) appears only for the ATP-SAMs (Figure S5b). We emphasize that this band is also not present in the conventional Raman spectrum of ATP. Interestingly, the Gauss fit of the marker band of DMAB at ∼1384 cm^–1^ reveals that this band is actually composed of several bands, particularly a small band at ca. 1360–1370 cm^–1^ which indicates the existence of a yet unknown compound (see Supporting Information, Figure S1c,d). In comparison to the band position of the NO_2_ group of NTP acquired during conventional Raman and SERS measurements (Table 1), one can then safely assume that the reaction of ATP in HCl_aq_ (under plasmonic conditions) leads to the formation of a new compound, most likely due to the presence of active hot carriers in the strong electromagnetic field, as hot carriers-induced reactions can strongly influence SERS spectra.^31^ Although the redox reactions of ATP and NTP have been the subject of numerous studies,^32^ the intermediates formed within the monomer pathway have not been characterized in detail, yet. As detailed above, the NO_2_ and ring stretching modes enable a clear distinction between the compounds formed in the reaction. However, due to the similarity of the chemical structures, bands in the SERS spectra can overlap. Even with time-dependent measurements (Figure S6), a clear identification of compounds is difficult, and thus, possible intermediates or other compounds might be overlooked. Notably, if O–H, N–H, and C–H groups were substituted by O–D, N-D, and C–D groups during the reactions, this should result in isotopic band shifts in the SERS spectra in the spectral regions from ca. 3000–4000 cm^–1^ to ca. 2000–3000 cm^–1^.^33−35^ It is also noteworthy that no deuterium-related signal (except for D_2_O bumps) was detected in the Raman-silent region (ca. 1800–2800 cm^–1^) in any NTP and ATP sample (Figure S7). Thus, an H-D substitution can be excluded. Currently, the investigation of potential intermediates has been suggested mainly via theoretical studies^36,37^ and the monomer and dimer pathways were discussed separately. The relation between the two pathways has not been studied experimentally so far. In contrast to the ring stretching mode, the new band at ca. 1360–1370 cm^–1^ is more substantial for the identification of new compounds. Consequently, it is important to verify whether the reactions starting from either ATP or NTP yielded identical compounds.

**Figure 1:**
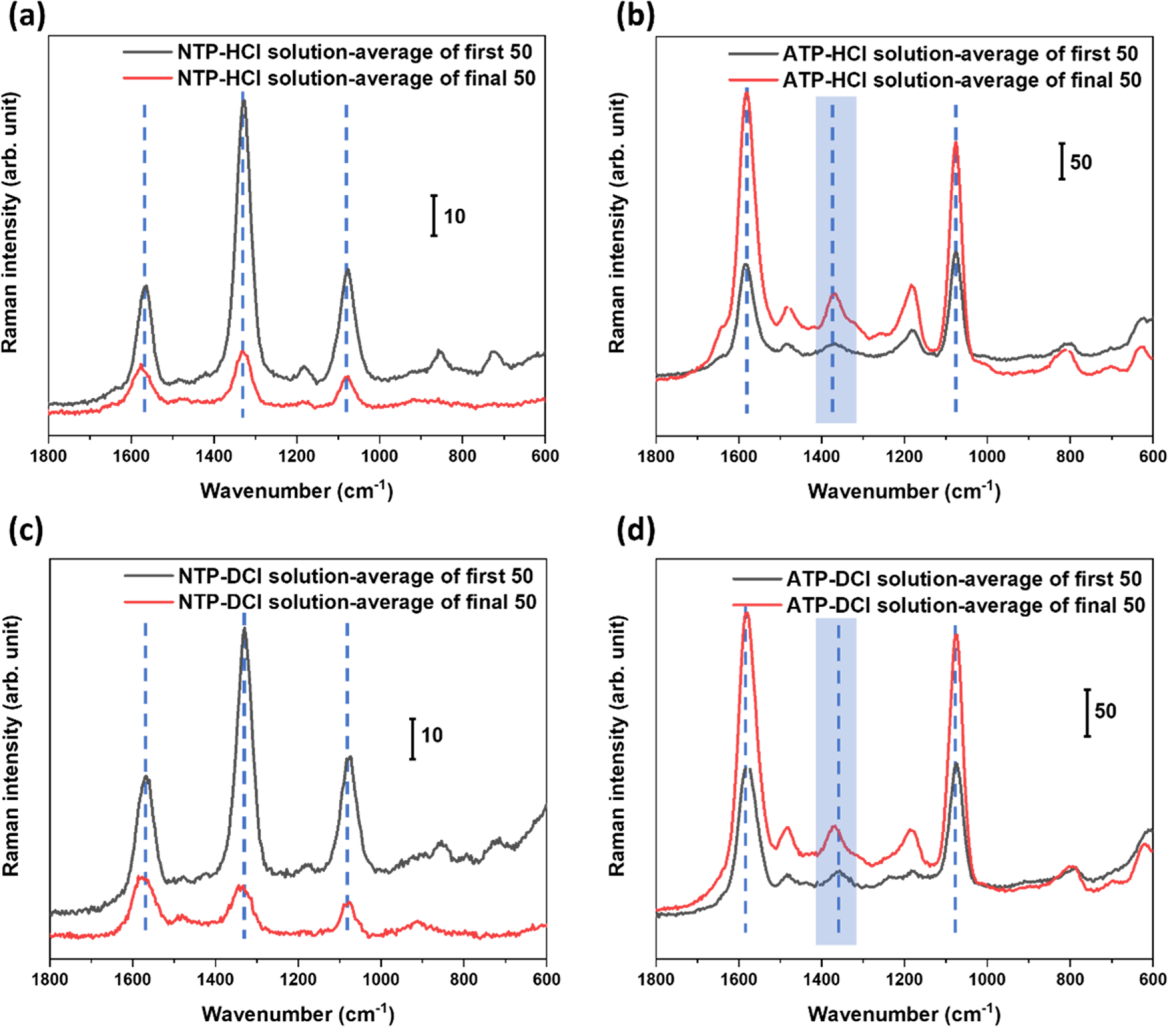
(a) SERS spectra of ATP and NTP in the presence of HCl_aq_. The ring stretching (left line) and the NO_2_ mode (middle line) indicate the newly formed product. Average SERS spectra from time-dependent measurements of NTP (a, c) and ATP (b, d) in the presence of HCl_aq_ and DCl_aq_ are from Figure S6. The red and black spectra show the average of the first and last 50 spectra of the experiments, respectively. The characteristic bands are highlighted with blue dashed lines. The blue shades in (b) and (d) indicate the positions of the new band. The assignments of these spectra are provided in Table S1 (Supporting Information). λ = 532 nm, *P* = ∼280 μW, *t*_acq_ = 0.5 s, 1acc.

In addition to the occurrence of the new band, clear band shifts on both NO_2_ and ring stretching bands were observed in the time-dependent experiments of NTP- and ATP- SAMs on SERS substrates. The NO_2_ band is relatively stable within ∼2 min while the ring stretching mode of NTP in HCl_aq_ at ∼1565 cm^–1^ is shifted by ∼10 cm^–1^ (Figure 1a). Almost no band shifts were observed from the spectra of ATP (Figure 1b) up to ∼13 min in HCl_aq_, but a new broad band at ∼1370 cm^–1^ was detected, which is indicated by the blue shade. Importantly, the intensity of the NO_2_ band of NTP is higher or comparable to that of the ring stretching mode while in the case of ATP, the ∼1370 cm^–1^ band is much weaker than the corresponding ring stretching mode. In other words, an assignment to the NO_2_ band can be excluded. Interestingly, in the presence of DCl_aq_, both the NO_2_ band and ring stretching band of NTP showed a blue shift by ca. 5–10 cm^–1^ (Figure 1c). In contrast, in the ATP spectra recorded in the presence of DCl_aq_, the new band at ∼1360 cm^–1^ shifted by ca. 5–10 to ∼1370 cm^–1^ (see the blue shade of Figure 1d). Additional measurements where band shifts were observed are presented in Table S1, and several factors are suspected to cause these shifts in HCl_aq_ and DCl_aq_ such as metal–adsorbate interactions, hydrogen bonds and the chemical modification of the side group (NO, NO_2_, NH_2_). Since it is highly likely that the substrate properties and the experimental conditions affected the reactions, a direct comparison of the current results with previous ones where colloidal Ag and Au/Pd nanostructures were used is not applicable.^16,38^ It has been reported that hydrogen bonds between N–O···H_2_O can induce band shifts.^39−41^ Apparently, the influence of the hydrogen bond depends on the susceptibility of the side group. It is supposed that band shifts in HCl_aq_ and DCl_aq_ are caused by H_2_O and D_2_O and not by the proton (H^+^, D^+^). For instance, it has been reported that H_2_O has different intensities of hydrogen bonds compared to D_2_O^42^ and might explain the band shifts difference of NTP and ATP in HCl_aq_ and DCl_aq_. The pH-dependent experiments of NTP-SAMs on SERS substrates (Supporting Information, Figure S2) further support the involvement of H_2_O directly or indirectly in the reactions. Therefore, not only the effect of surface interactions between Ag and thiols must be considered but also possible hydrogen bonds between NTP/ATP and water molecules in HCl_aq_/DCl_aq_. Thus, with respect to the band shift issue, we postulated that the ∼1364 cm^–1^ band acquired from solutions in Figure S5b has an original position at ∼1360 cm^–1^ or even at a lower wavenumber, which is affected by the measured environments. At this point, it is noteworthy that a quantitative evaluation of the influence of hydrogen bonds via H_2_O and D_2_O and the respective exchange is complex and beyond the scope of this work. Additionally, band shifts caused by the formation of new product should be also considered. Inspired by the different SERS spectra of NTP and ATP in HCl_aq_/DCl_aq_ and the potential water effect, in the next step, we conducted experiments under acidic vapor to reduce the influence of water.

### Detection of a Stable Compound in Acidic Vapor

Under ambient conditions, HCl_aq_ (1 mol/L) has a high vapor pressure and HCl_g_ dominates in the gas phase which effectively suppresses possible competitive reactions induced by water. Furthermore, working in a gaseous environment reduces (or at least slows down) the possible destruction, e.g., the detachment or reshaping of the AgNP by direct solvent interaction. The experimental sketch using acidic vapor is presented in Approach 2, Scheme 1b.

**Figure 2:**
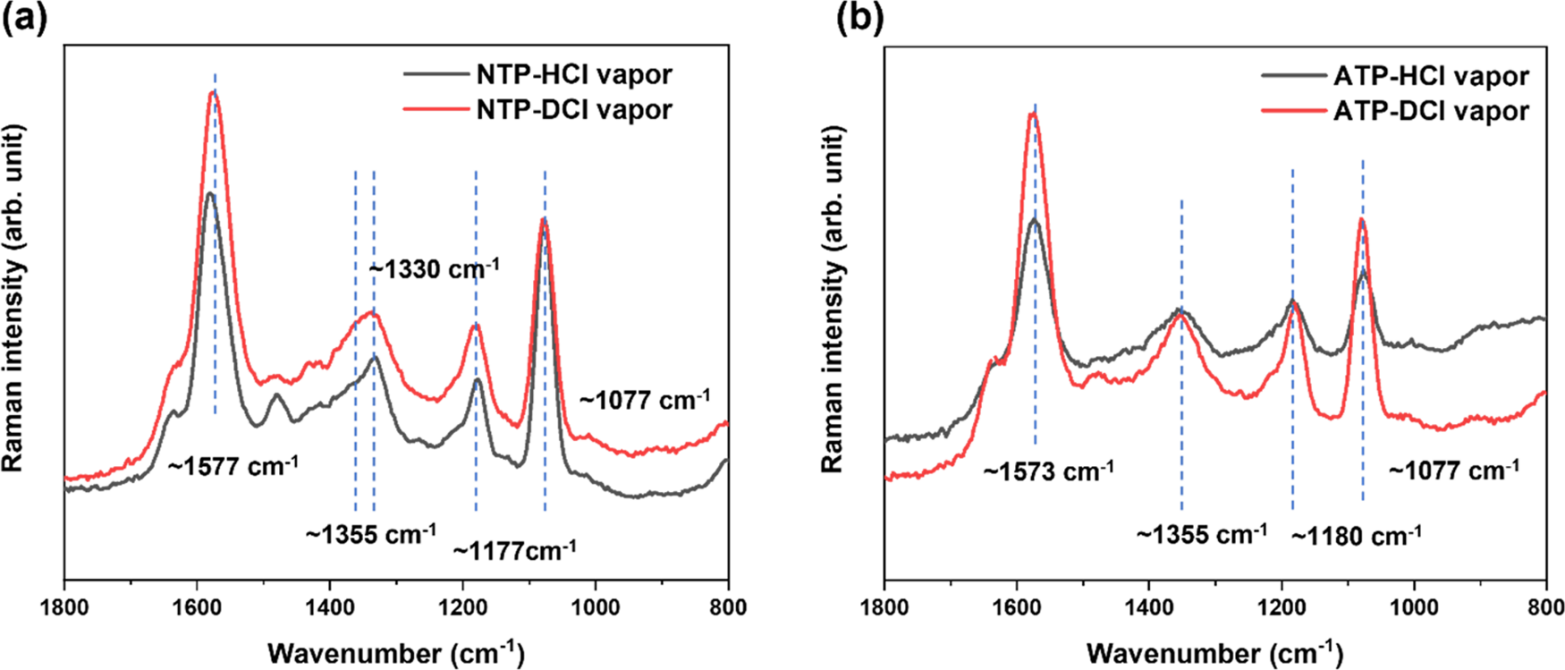
SERS spectra of (a) NTP- and (b) ATP-SAMs treated with HCl_g_/DCl_g_. λ = 532 nm, *P* = ∼280 μW, *t*_acq_ = 1 s, 20 acc. Each spectrum is an average of 3–6 spectra acquired on different sites on the samples. The blue dashed lines indicate the characteristic bands and their centers. The new band at ∼1355 cm^–1^ can be assigned to an N=O mode. The highly similar spectra in (a) and (b) point to the formation of the same chemical compound proposed to 4-nitrosothiophenol (TP*).

**Figure 3:**
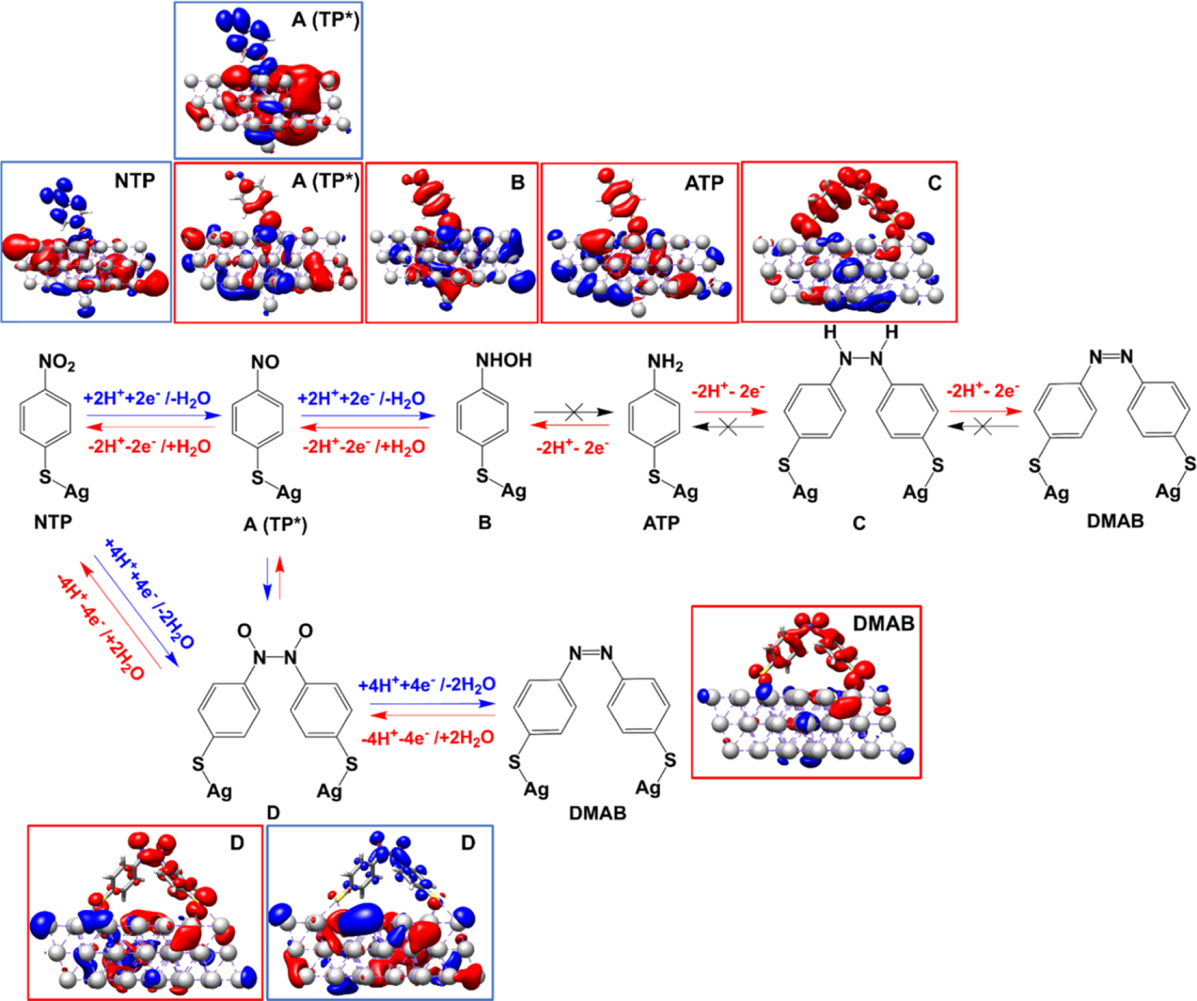
Charge density differences (CDDs) of the molecular-plasmonic system illustrating the nature of the low-lying dipole-allowed excitations at 532 nm. In the CDD images, charge transfer takes place from the red (hole) to the blue (electron) region. Accordingly, CDDs in blue frames are associated with the reduction of the surface-immobilized molecules (transferring charge from metal to molecule) while CDDs in red frames represent substrate oxidation (transferring charge from molecule to metal). Potential reaction pathways connecting NTP and ATP and further to DMAB are shown. Pathways accessible by light-driven processes at ∼2.33 eV (532 nm, see CDDs) are indicated by arrows (red: oxidative; blue: reductive). Photophysically inaccessible pathways (for energetic reasons) are crossed out (black X). Intermediates A and D can be (photo)reduced or oxidized at the given excitation wavelength of 532 nm, see respective CDDs. A: 4-nitrosothiophenol, TP*; B: 4-hydroxylaminothiophenol; C: 4,4′-dimercaptohydrazobenzene; D: 4, 4′-dimercaptoazodioxybenzene.

Like the previously discussed reactions of NTP- and ATP-SAMs in HCl_aq_, under vapor conditions (Figure 2), a broad band at ca. 1350–1360 cm^–1^ was detected in the SERS spectra. Most importantly, the similarity of the spectra suggests that under liquid and gaseous HCl reaction conditions, the same compound was formed. The band position of the ring stretching modes at ca. 1570–1580 cm^–1^, indicates that the functional group at the phenyl ring of the compound is neither NH_2_ nor NO_2_. According to previous theoretical^36,37^ and electrochemical approaches^43^ and our quantum chemical simulations, 4-nitrosothiophenol (−S-C_6_H_4_–NO) and 4-hydroxylaminothiophenol (−S-C_6_H_4_–NHOH) are the most likely structures (refer to Figure 3). To further verify the structure of this compound, a deuterium strategy using DCl_aq_ and D_2_O was applied. This allowed us to examine whether hydrogen or deuterium was involved in the respective reactions. Therefore, in subsequent experiments, the reactions were repeated with DCl_g_. It is obvious that the spectra of the reaction products from ATP and NTP in the presence of DCl_g_ are very similar to those recorded in the presence of HCl_g_ (Figure 2). In the spectra, neither the O–D, N-D nor C–D bands were observed (see also the Supporting Information, Figure S8). More importantly, the fitting of the new band in the 1300–1400 cm^–1^ region in the NTP spectrum in Figure 2 shows that the band at ∼1355 cm^–1^ is clearly different from the NO_2_ band position at ∼1330 cm^–1^ (Supporting Information, Figure S9). Consequently, the ∼1355 cm^1^ band must originate from a new compound with a structure different from those of ATP and NTP. The position of this new band under HCl_g_/DCl_g_ vapor conditions shows a slight shift compared to the band (ca. 1360–1370 cm^–1^) under liquid conditions (Figure 1). Slight band position shifts among spectra acquired under vapor and solution conditions are most likely due to environmental changes (e.g., concentration gradients, etc.) while the droplet evaporates. As those conditions are difficult to control and the effects on the spectra are comparatively small, we will ignore those in this study. In summary, the results of Figures 1 and 2 indicate that the same compound was formed and was assigned to −S-C_6_H_4_–NO (4-nitrosothiophenol, further denoted as TP*). Additionally, Tsutsumi’s work also supports the existence of TP*. According to the reported spectral positions of the nitroso group,^44^ our theoretical investigations and the spectrum of a similar nitroso compound (Figure S10), the band at ∼1355 cm^–1^ under acidic vapor conditions can be assigned to an N=O mode. The detection of an S–C_6_H_4_–NHOH intermediate can be excluded, as no bands or band shifts that indicate an H-D substitution were observed. To the best of our knowledge, this is the first time that such a compound has been experimentally detected in research on plasmonic catalysis. To further confirm our experimental results, computational modeling was conducted.

### Computational Modeling of Reaction Routes and Involved Intermediates

Theoretical modeling of the involved surface-immobilized reactants on a Ag slab based on periodic density functional theory (DFT) and nonperiodic time-dependent DFT simulations (TDDFT, see Supporting Information for details) were performed to corroborate the experimental results. Thereby, we apply our recently introduced computational approaches to assess the spectra and thermodynamic properties of plasmonic hybrid system.^45−50^ Based on our computational approach and referred to previous works,^36,37,51^ we explore the given plasmon-driven reaction along ground-state reaction pathways by periodic DFT calculations while the excited-state properties of the involved intermediates are addressed by means of TDDFT. The driving forces associated with the given reactions were approximated by balancing the reactions using isolated H_2_O or H_2_ molecules. Intermolecular interactions such as hydrogen bonds or H_2_O/H_2_ adsorption on the Ag slab were not considered, which might affect the predicted driving forces to a certain degree. On the basis of charge density differences (CDDs), which depict localized electron and hole distributions, we examined the lowest 600 electronic states (containing both the Ag-slab and the reactant) regarding their respective electronic character (molecule-only, metal-only, metal-to-molecule, molecule-to-metal charge transfer). The high number of electronic states is necessary to address the electronic transitions accessible within the experimental laser excitation region (2.33 eV, corresponding to 532 nm). In the following, we restrain from the discussion of specific electronic excitations, as the plasmonic hybrid system model features a plethora of highly mixed weakly absorbing transitions, which is in particular evident in the case of charge transfer processes involving the Ag slab and the surface-immobilized substrate. Details regarding the respective transitions are summarized in Supporting Information.

The performed computational studies demonstrate that the conversion of the dimer pathway (NTP to DMAB) takes place in three steps: Initially, NTP is reduced to the intermediate TP* (S–C_6_H_4_–NO). It is clear from the variety of (weakly dipole-allowed) electronic states of the surface-immobilized NTP that those states accessible in the vicinity of 2.33 eV (532 nm) are mainly Ag → NTP transitions (see Figure 3 (NTP)), indicating that upon light excitation, NTP is reduced to TP*.

**Figure 4:**
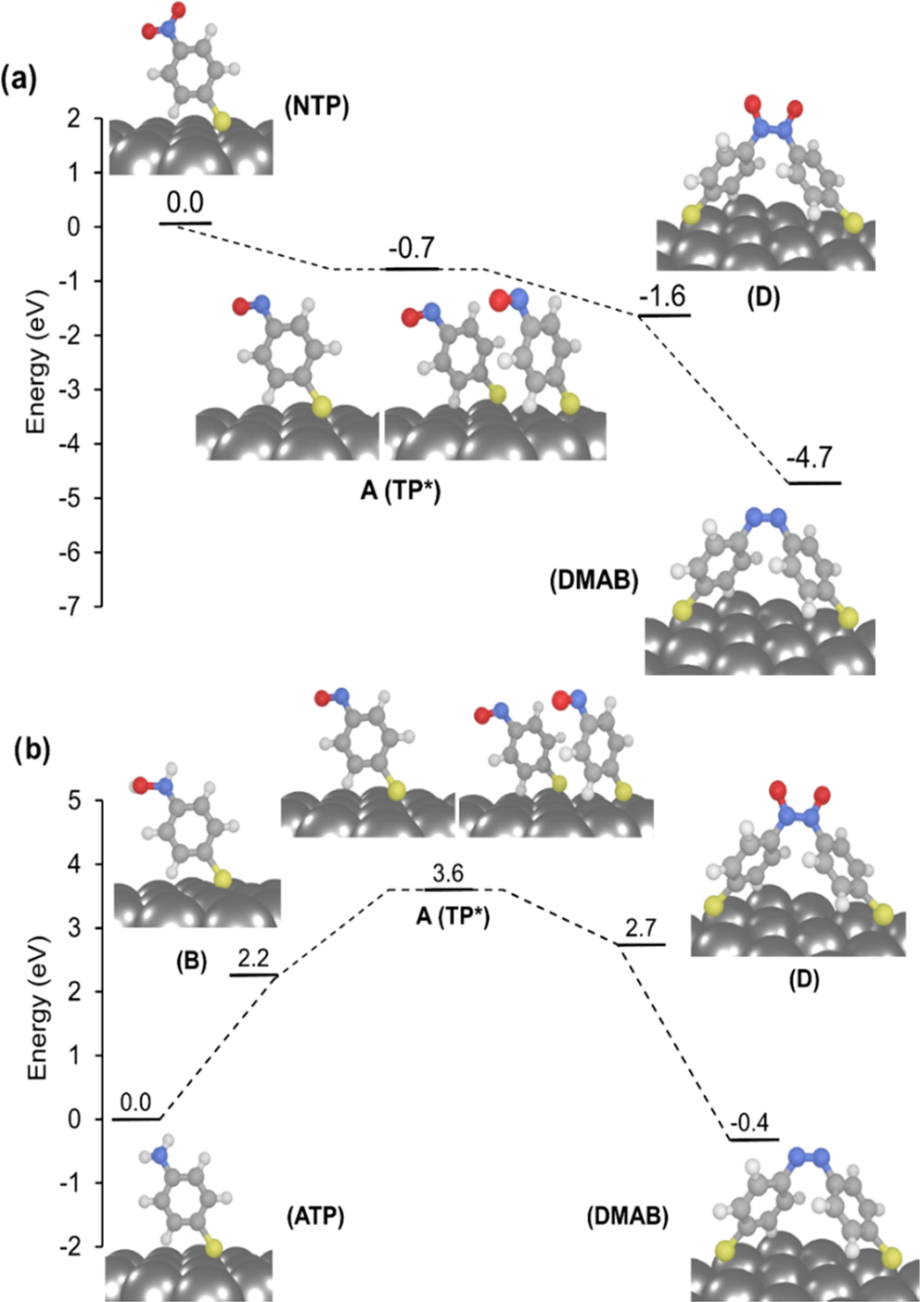
Ground-state reaction pathways for the formation of DMAB from NTP (a) and ATP (b), as predicted by periodic DFT calculations. The driving forces (Δ*G*) for each stepwise redox reaction are shown. Structures of the surface-immobilized intermediates formed in different reaction pathways are illustrated.

Subsequently, two TP* molecules dimerize to form another intermediate (S_2_–C_12_H_8_-(NO)_2_) (see Figure 3D). The CDDs of this intermediate illustrate that it has the potential for both oxidation and reduction under light excitation in the experimental excitation window, as evident from both metal-to-molecule as well as molecule-to-metal transitions. Thus, the photoinduced reduction of D leads to the formation of DMAB. The thermodynamical quantities (see Figure 4a) confirm that these are energetically accessible under the experimental conditions. In reverse, the predicted light-driven charge transfer from DMAB to the silver surface indicates that DMAB can be oxidized to form D, while D is further oxidized to form TP* (Figure 3). The formation of TP* from DMAB is therefore accessible via charge transfer excited states in the range of laser radiation (2.33 eV).

Moreover, computationally, it can be shown that further reduction of TP* to B is possible due to the presence of energetically low-lying charge transfer transitions from the Ag slab to the molecule. However, as soon as B is formed, it will be photooxidized immediately to TP* as intermediate B features a plethora of substrate-to-silver charge transfer states at the excitation wavelength of the laser. Thus, B is converted to TP*, while a conversion from B to ATP is highly unfavorable due to the lack of charge transfer states (at 2.33 eV) which are associated with an enhancement of the electronic density of the surface-immobilized B. Therefore, the reaction’s course is mainly governed by the nature of the energetically accessible excited states and not by driving forces within the electronic ground state. The nature of the light-driven charge transfer processes among the Ag cluster and the various intermediates of the substrate have been carefully investigated by means of CDDs of the electronic transitions in the region of the excitation wavelength of 532 nm, see Figure 3. As becomes evident from these CDDs, the charge transfer from B toward the silver surface indicates that only oxidation can occur at 532 nm. Electronic excitations associated with the (photo)reduction of B are energetically inaccessible. Since all electronic states are oxidative (see Supporting Information, Table S6), the only possible reaction pathway of B leads to its oxidation and the recovery of TP*.

We propose two possible pathways leading to DMAB formation from ATP: (1) Dimerization of two ATP molecules yields the intermediate C, which is subsequently oxidized to DMAB, and (2) stepwise double oxidization of ATP to intermediate B and further to TP*; see upper and lower reaction pathways in Figures 3 and 4b. Finally, the reaction step TP* → DMAB is identical to that in the NTP pathway. Based on the performed quantum chemical simulations, both reaction pathways leading to the formation of DMAB are feasible within the excited states accessible with 532 nm excitation. However, the experiments indicate that pathway 2 seems to be favored. Furthermore, from the nature of the light-driven processes as predicted at the TDDFT level, it can be concluded that charge transfer from DMAB to the silver nanoparticle will oxidize DMAB to intermediate D, while the formation of C from DMAB is not possible at the given excitation wavelength. Finally, further (photo)oxidation of the sample leads to the intermediate TP*.

Our computational study suggests that the conversion of NTP to TP* is reversible, which is verified by experiments in the following section (Route 3, Figure 6a). According to the theoretical modeling, a photoinduced charge transfer occurs from the molecule to the silver surface, leading to the oxidation of the intermediate TP* and finally to NTP. However, TDDFT also predicts charge transfer processes in the opposite direction, which leads, eventually, to the reduction of the surface-immobilized thiolates. These charge transfer processes are related to the DMAB formation. Therefore, it is possible to regain intermediate TP* as well as NTP from DMAB because the electronic transitions are accessible at 532 nm excitation. On the other hand, this is not the case for ATP: The reduction of TP* to ATP requires the formation of intermediate B. However, the excited states of B in resonance with the given excitation wavelength are of molecule-to-silver charge transfer character. Thus, an immediate photooxidation of B back to TP* occurs while a photoreduction of B and the formation of ATP, i.e., charge transfer in the opposite direction, is only possible at considerably higher excitation energies. Thus, ATP cannot be regained from DMAB. We note that the subsequent dissociation of DMAB to TP* occurs via intermediate D.

### Investigation of the Pathway Relations in Time-Dependent SERS Experiments

**Figure 5:**
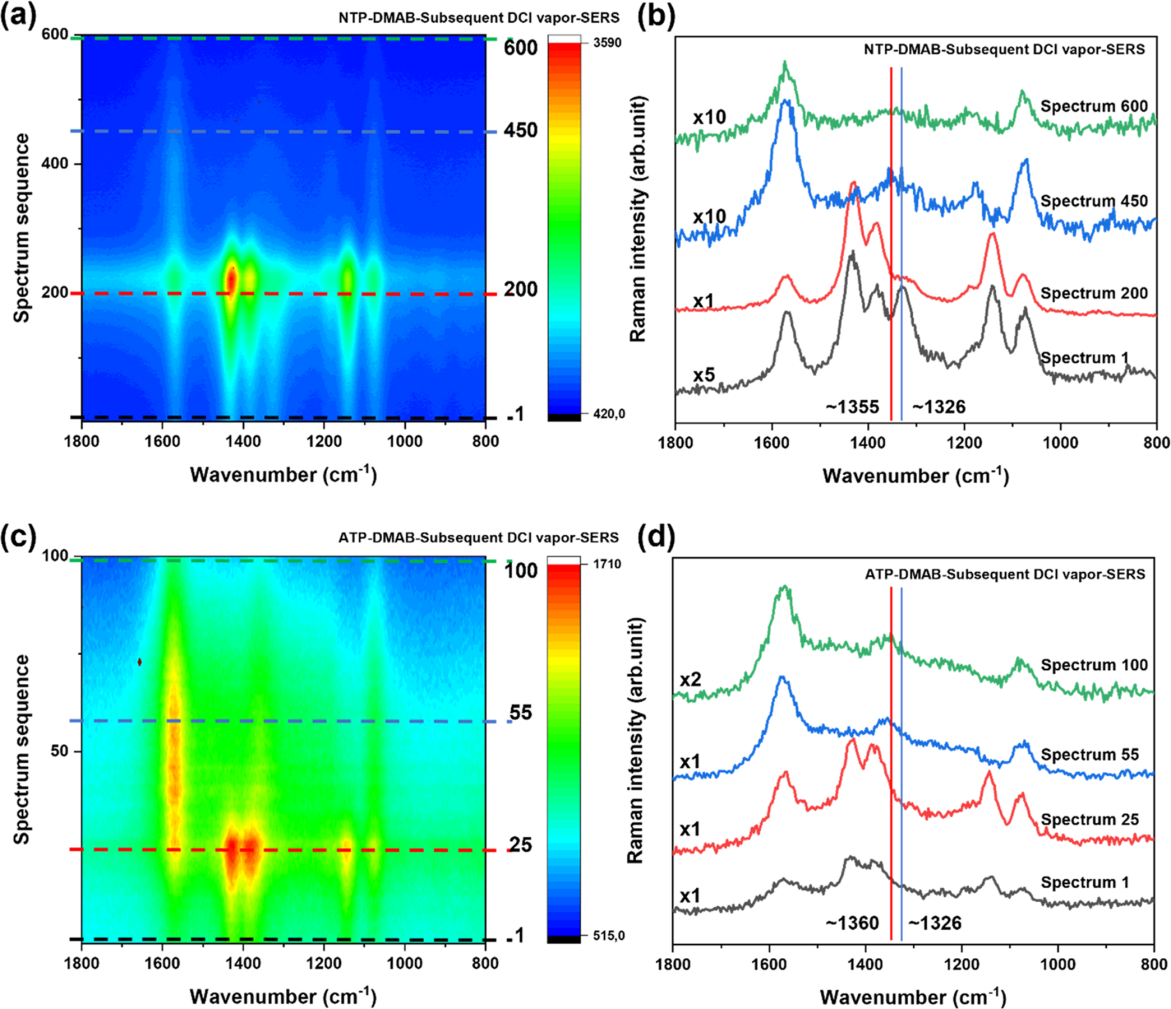
Time-dependent SERS spectra of (a) NTP- and (c) ATP-SAMs dimerizing on SERS substrates followed by the treatment with DCl_g_. (b, d) Selected spectra corresponding to contour plots labeled by dashed lines (indicated by the same color), which show the important reaction steps of the conversion from DMAB to TP*. In the selected spectra in (b) and (d), the transition of DMAB to TP* is clearly visible. The red lines indicate the NO band (1350 cm^–1^), and the blue lines indicate the ring stretching and NO_2_ modes (1326 cm^–1^). All spectra are recorded with λ= 532 nm, *P* = ∼280 μW, and t_acq_ = 0.5 s/spectrum.

**Figure 6:**
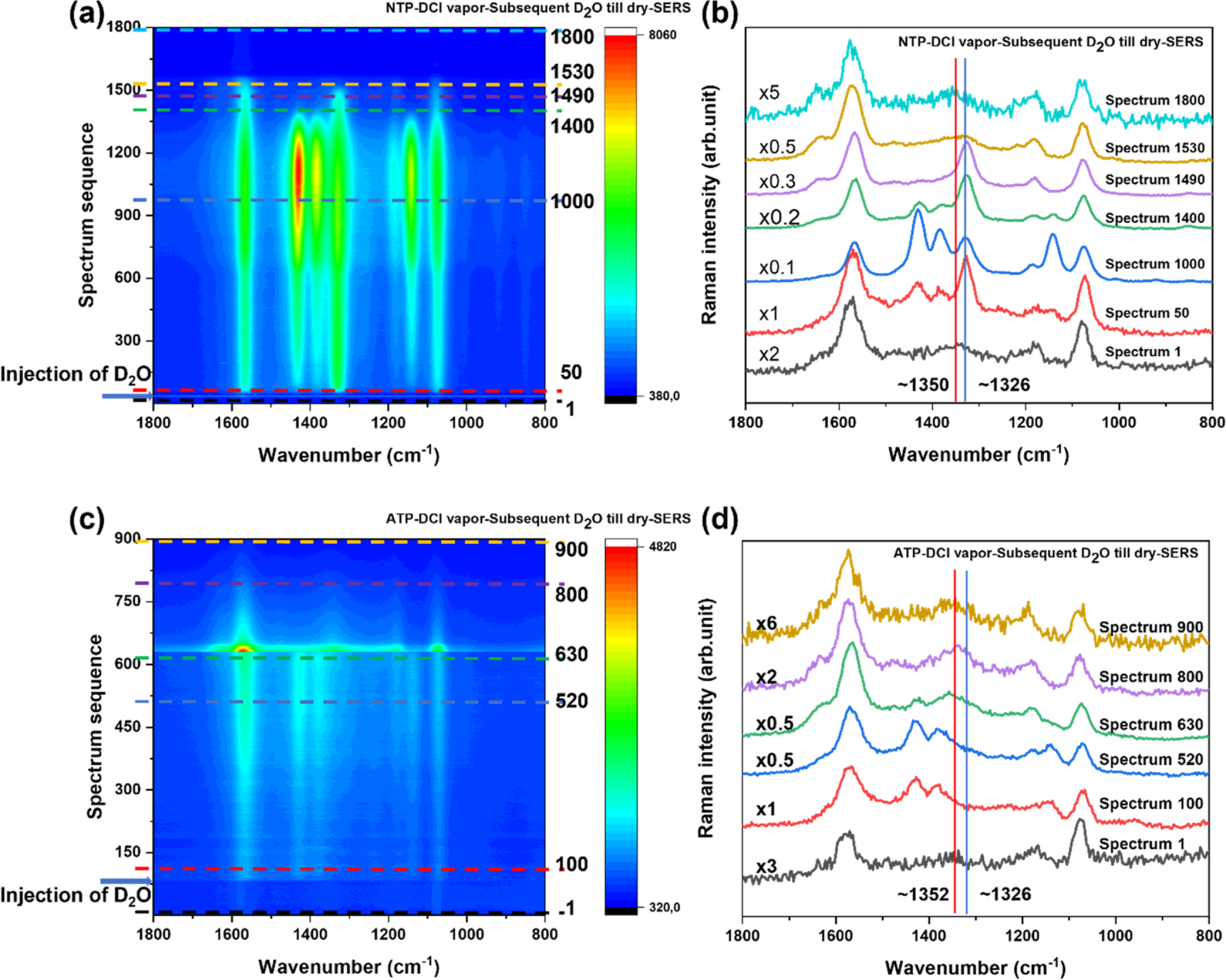
Time-dependent SERS spectra of (a) NTP- and (c) ATP-SAMs treated with DCl vapor followed by treatment with D_2_O. (b, d) Selected spectra corresponding to contour plots labeled by dashed lines (indicated by the same color), which show the important reaction steps of the conversion from TP* to DMAB. The red lines indicate the NO band (∼1350 cm^–1^), and the blue lines indicate the ring stretching and NO_2_ modes (∼1326 cm^–1^). All spectra are recorded with λ = 532 nm, *P* = ∼280 μW, and *t*_acq_ = 0.5 s/spectrum.

To follow the dynamic transformations of the components in the reactions and experimentally identify those intermediates and the possible connection of two pathways discussed in the theoretical modeling in Figure 3, time-dependent SERS experiments were performed. In time-dependent experiments, the samples were irradiated continuously (Approach 3 and 4, Scheme 1d,e), and a spectrum was recorded every 0.5 s. Under these conditions, the reaction processes can be traced reliably, and the relations between the dimer and monomer pathways could be explored in more detail. The reaction steps of different pathways involving DMAB-NTP-TP* and DMAB-ATP-TP*, respectively, were investigated using DCl_g_ (Figures 5 and 6) and HCl_g_ (Supporting Information, Figures S11–S12).

Figure 5a shows the time trace of the spectra starting with NTP as an image plot. The data were collected on dried samples that were treated with DCl_g_ during the experiment. The transition in the spectra from DMAB to TP* was very smooth and was clearly observed around spectrum number 450. Under irradiation, NTP dimerized quickly to DMAB and the NO_2_ band decreased, accordingly. In the presence of DCl_g_, the DMAB signals gradually decreased, while the broad N=O band increased simultaneously. During this process, very weak NO_2_ bands were observed in both DCl_g_ and HCl_g_ cases. At the end of the experiment, no clear NO_2_-related bands could be detected, which pointed to the formation of TP*. The decreasing TP* signal at the end of the measurement is likely caused by a slow SERS substrate degradation in the presence of the acidic vapor. A Gauss-fitting procedure was applied to separate the bands (Figure S13). Under the chosen experimental conditions, the N=O band was detected at ∼1355 cm^–1^. This path (NTP → DMAB → TP*) is termed Route 1 (see Scheme 1c) indicating the reactions from NTP to TP* under vapor conditions.

In the case of ATP (Figure 5c), a similar course of the reaction was observed. When the ATP sample was irradiated, DMAB signals appeared immediately and increased prior to the influence of DCl_g_. At longer DCl_g_ incubation, the DMAB signal intensity gradually decreased. Remarkably, no C–D, N-D, and O–D bond formation was detected (Figure S8). Referring to the observations in Route 1, we suggest that DMAB directly dissociated to TP*. Gauss-fitting provides a reliable recognition of the N=O band at ∼1360 cm^–1^ (Figure S14). This pathway is termed Route 2 (ATP → DMAB → TP*) indicating the reaction of ATP to TP* (Scheme 1c).

In the next step, we examined the reverse reaction from TP* to DMAB as a function of time. Figure 6a illustrates the conversion of TP* to DMAB on an NTP-SAM sample. Figure 6b shows spectra at selected time points. These spectra highlight the most important reaction changes observed for the conversion of TP* to DMAB.

In the experiment, the NTP-SAM-covered SERS substrate was first treated with DCl_g_. TP* was detected during the subsequent continuous irradiation, as expected from Figure 5a. Finally, the sample was treated with D_2_O by dropping a small volume into the same illumination position without switching off the laser. Obviously, the reaction was different from Routes 1 and 2. An increase of the NO_2_ band (∼1326 cm^–1^) was observed, followed by the appearance of DMAB marker bands, indicating that TP* was oxidized to NTP, which then dimerized to DMAB. Interestingly, during the evaporation of D_2_O the NO_2_ band appeared and dominated the spectra again, but the intermediately produced NTP finally converted to TP*. However, from these spectra, it was not possible to determine the ratio of this route (TP* dimerized into DMAB via NTP, TP* → NTP → DMAB) versus a direct dimerization of TP* (without via NTP, TP* → DMAB). In our control experiments (Figure S15), the capability of Ag^+^-induced oxidation of DMAB to NTP was assessed. Presumably, the injection of D_2_O affected the reaction routes by changing the pH of the sample. In addition, electron-accepting Ag^+^ cations were formed, and hot carriers were generated, which oxidized TP* to NTP. A Gauss fit in the range ca. 1300–1400 cm^–1^ shows that the N=O band slightly shifted from ∼1353 to ∼1350 cm^–1^ (Figure S16). This route (NTP → TP* → NTP → DMAB → NTP → TP*) is referred to as Route 3 (Scheme 1c), indicating the reversibility of the reactions between TP* and DMAB in the presence of D_2_O on the NTP samples. The possible individual reaction steps are summarized in Supporting Information, Scheme S1.

Similar results were found in the reaction of TP* to DMAB starting from ATP (Figure 6c). The ATP-SAM on the SERS substrate was first treated with DCl_g_. TP* was detected during subsequent continuous irradiation. After the addition of D_2_O to the illumination area, the N=O-related band quickly disappeared within a few seconds, accompanied by the rise of DMAB signals. Apparently, no clear NO_2_ mode was detected during the reaction. During the evaporation of D_2_O, hardly observable NO_2_ bands were detected (Figure 6d, selected spectrum 630). Compared to the distinct NO_2_ bands at the same step of the aforementioned NTP case, it seems that a reaction process from DMAB to NTP on ATP samples is energetically unfavorable. It remains unclear why the formation of NTP was prevented. Additionally, only in 1 out of 30 time-dependent experiments (Figure S17), potential ATP-related bands were detected in one spectrum prior to the formation of DMAB, which also supports the theoretical results that the formation of ATP is unfavorable. In this regard, several aspects should be considered. First, the increasing energy level from ATP → TP* → NTP renders this (oxidation) pathway thermodynamically unfavored. Second, our results indicate the influence of external parameters, i.e., presence of DCl/HCl and D_2_O/H_2_O. Finally, it has been reported that surface catalytic reactions can be affected by both energy barrier and steric hindrance of thiolates.^52^ In fact, without an apparent characteristic band such as NO_2_ or NO, the signals of ATP are easily obscured by others and can hardly be distinguished. Thus, from these spectra, limited by the time resolution and signal-to-noise ratio of the spectra, it is difficult to confirm or disprove the formation of ATP. A sudden increase in band intensity was observed at spectrum 640, and it can only be assumed that this was caused by the evaporation of water. Interestingly, this observation was found only with the reaction of ATP and never occurred with NTP. A Gauss fit of the bands in the range ca. 1300–1400 cm^–1^ shows a shift of the N=O band from 1359 to 1352 cm^–1^ (Figure S18). This route (ATP → TP* → DMAB → (NTP) → TP*) is referred to as Route 4 (Scheme 1c) indicating the reversible reaction of TP* and DMAB on ATP samples in the presence of D_2_O. The possible individual reaction steps are summarized in Supporting Information, Scheme S2.

In summary, the time-dependent experiments verify the computational analysis, which provides several new insights into the common and newly recognized reaction steps involved in the monomer and dimerization pathways of NTP and ATP. Notably, the reversibility on the NTP side (DMAB-NTP-TP*) is very convincing. The injection of D_2_O or H_2_O after treating the samples with DCl or HCl vapors clearly shows the direction of the reaction. This indicates that environmental controls could definitely influence the energy of metal–adsorbate bonding/antibonding orbitals, leading to the different selectivity of the chemical reactions. ATP formation, however, was difficult to confirm in this study. Apart from the absence of characteristic bands of ATP to other thiolates, insufficient time resolution and the signal-to-noise ratio of the spectra may also prevent ATP detection. Following previous studies on the electron-based reduction of NTP,^16,23^ the oxygen source of the reactions of NTP and ATP is assumed to be different. The oxygen in TP* produced during the reduction of NTP most likely comes from the NO_2_ group, whereas the oxygen in TP* produced during the oxidation of ATP must originate from atmospheric O_2_ or H_2_O. Atmospheric O_2_ can affect the reaction of ATP → DMAB as has been demonstrated previously^53^ and may be also a potential factor affecting the reaction rate of ATP → TP*. As the majority of the reported reactions take place under ambient conditions, this will not affect our conclusions.

Finally, our theoretical studies do not predict ATP formation. A comparison among different SERS substrates considering the influence of surface composition and adlayers on nanoparticles would be helpful to address the formation of ATP in a further stage.

## Conclusions

In this study, the plasmon-induced redox reactions of 4-nitrothiophenol (NTP) and 4-aminothiophenol (ATP) under different acidic conditions on silver island film-based SERS substrates were systematically investigated. Physical vapor deposition SERS substrates were used to exclude the influence of any additional chemicals. In HCl solution, band shifts were observed indicating the involvement of water, however, mostly as an environmental parameter, not as an actual reactivity influence. Most importantly, a new band was observed in the SERS spectra of the ATP samples. Motivated by the influence of water and the detection of a new band in acidic solutions, the experimental conditions were changed from the liquid–solid interface toward a vapor–solid phase. These experimental conditions enabled, for the first time, the successful experimental recognition of a new stable compound, namely, 4-nitrosothiophenol (TP*) in the reaction of NTP- and ATP-SAMs on SERS substrates. Periodic DFT and TDDFT simulations addressed thermodynamic properties and charge transfer contributions, respectively, and confirmed the existence of TP* in the monomer pathway. The identification of this compound manifests the selectivity of plasmon-induced catalysis. Moreover, the results also enabled the definition of two different pathways (monomer and dimer pathways) of the redox reactions of ATP and NTP in the SERS experiments. With the control of reaction environmental parameters, time-dependent SERS experiments allowed detailed monitoring of the dynamic transitions among different thiolates, and indeed, TP* was found to be the only stable intermediate in our study. Furthermore, it could be shown that the two pathways are closely connected. Last but not least, a deeper insight into the influence of experimental conditions on the redox reaction pathways of thiolates in plasmon-induced catalysis was comprehensively presented.

## Supporting Information Available

CDDs images and UV–vis spectra (PDF)
Raman spectra of ATP and NTP, SERS spectra of ATP- and NTP-SAMs on Ag SERS substrates, and AFM images and UV–vis spectra of substrates (PDF)

## References

[ref1] Chen K.; Wang H. Plasmon-Driven Photocatalytic Molecular Transformations on Metallic Nanostructure Surfaces: Mechanistic Insights Gained from Plasmon-Enhanced Raman Spectroscopy. Mol. Syst. Des. Eng. 2021, 6, 250–280. 10.1039/D1ME00016K.

[ref2] Brongersma M. L.; Halas N. J.; Nordlander P. Plasmon-Induced Hot Carrier Science and Technology. Nat. Nanotechnol. 2015, 10, 25–34. 10.1038/nnano.2014.311. 25559968

[ref3] Zhang Y.; He S.; Guo W.; Hu Y.; Huang J.; Mulcahy J. R.; Wei W. D. Surface-Plasmon-Driven Hot Electron Photochemistry. Chem. Rev. 2018, 118, 2927–2954. 10.1021/acs.chemrev.7b00430. 29190069

[ref4] Zhang Z.; Zhang C.; Zheng H.; Xu H. Plasmon-Driven Catalysis on Molecules and Nanomaterials. Acc. Chem. Res. 2019, 52, 2506–2515. 10.1021/acs.accounts.9b00224. 31424904

[ref5] Huang Y.-F.; Zhu H.-P.; Liu G.-K.; Wu D.-Y.; Ren B.; Tian Z.-Q. When the Signal Is Not from the Original Molecule To Be Detected- Chemical Transformation of para-Aminothiophenol on Ag during the SERS Measurement. J. Am. Chem. Soc. 2010, 132, 9244–9246. 10.1021/ja101107z. 20527877

[ref6] Sun M.; Xu H. A Novel Application of Plasmonics: Plasmon-Driven Surface-Catalyzed Reactions. Small 2012, 8, 2777–2786. 10.1002/smll.201200572. 22777813

[ref7] Zhang Z.; Kinzel D.; Deckert V. Photo-Induced or Plasmon-Induced Reaction: Investigation of the Light-Induced Azo-Coupling of Amino Groups. J. Phys. Chem. C 2016, 120, 20978–20983. 10.1021/acs.jpcc.6b03233.

[ref8] Zhang Z.; Deckert-Gaudig T.; Singh P.; Deckert V. Single molecule level plasmonic catalysis – a dilution study of p-nitrothiophenol on gold dimers. Chem. Commun. 2015, 51, 3069–3072. 10.1039/C4CC09008J. 25599345

[ref9] Thomas M.; Mühlig S.; Deckert-Gaudig T.; Rockstuhl C.; Deckert V.; Marquetand P. Distinguishing chemical and electromagnetic enhancement in surface-enhanced Raman spectra: The case of para-nitrothiophenol. J. Raman Spectrosc. 2013, 44, 1497–1505. 10.1002/jrs.4377.

[ref10] Love J. C.; Estroff L. A.; Kriebel J. K.; Nuzzo R. G.; Whitesides G. M. Self-Assembled Monolayers of Thiolates on Metals as a Form of Nanotechnology. Chem. Rev. 2005, 105 (4), 1103–1170. 10.1021/cr0300789. 15826011

[ref11] Zhang M.; Zhao L.-B.; Luo W.-L.; Pang R.; Zong C.; Zhou J.-Z.; Ren B.; Tian Z.-Q.; Wu D.-Y. Experimental and Theoretical Study on Isotopic Surface-Enhanced Raman Spectroscopy for the Surface Catalytic Coupling Reaction on Silver Electrodes. J. Phys. Chem. C 2016, 120, 11956–11965. 10.1021/acs.jpcc.6b02252.

[ref12] Wu D.-Y.; Liu X.-M.; Huang Y.-F.; Ren R.; Xu X.; Tian Z.-Q. Surface Catalytic Coupling Reaction of p-Mercaptoaniline Linking to Silver Nanostructures Responsible for Abnormal SERS Enhancement: A DFT Study. J. Phys. Chem. C 2009, 113, 18212–18222. 10.1021/jp9050929.

[ref13] Matsuda N.; Sawaguchi T.; Osawa M.; Uchida I. Surface-Assisted Photoinduced Reduction of p-Nitrothiophenol Self-Assembled Monolayer Adsorbed on a Smooth Silver Electrode. Chem. Lett. 1995, 24, 145–146. 10.1246/cl.1995.145.

[ref14] Zhang X.; Chen Y. L.; Liu R.-S.; Tsai D. P. Plasmonic Photocatalysis. Rep. Prog. Phys. 2013, 76, 046401 10.1088/0034-4885/76/4/046401. 23455654

[ref15] Gellé A.; Jin T.; de la Garza L.; Price G. D.; Besteiro L. V.; Moores A. Applications of Plasmon-Enhanced Nanocatalysis to Organic Transformations. Chem. Rev. 2020, 120, 986–1041. 10.1021/acs.chemrev.9b00187. 31725267

[ref16] Xie W.; Schlücker S. Hot Electron-Induced Reduction of Small Molecules on Photorecycling Metal Surfaces. Nat. Commun. 2015, 6, 7570 10.1038/ncomms8570. 26138619 PMC4506517

[ref17] Dong B.; Fang Y.; Chen X.; Xu H.; Sun M. Substrate-, Wavelength-, and Time-Dependent Plasmon-Assisted Surface Catalysis Reaction of 4-Nitrobenzenethiol Dimerizing to p,p’-Dimercaptoazobenzene on Au, Ag, and Cu Films. Langmuir 2011, 27, 10677–10682. 10.1021/la2018538. 21819110

[ref18] Kang L.; Xu P.; Zhang B.; Tsai H.; Han X.; Wang H.-L. Laser Wavelength- and Power-Dependent Plasmon-Driven Chemical Reactions Monitored using Single Particle Surface Enhanced Raman Spectroscopy. Chem. Commun. 2013, 49, 3389–3391. 10.1039/c3cc40732b. 23440353

[ref19] Qiu L.; Pang G. A.; Zheng G.; Bauer D.; Wieland K.; Haisch C. Kinetic and Mechanistic Investigation of the Photocatalyzed Surface Reduction of 4-Nitrothiophenol Observed on a Silver Plasmonic Film via Surface-Enhanced Raman Scattering. ACS Appl. Mater. Interfaces 2020, 12, 21133–21142. 10.1021/acsami.0c05977. 32286058

[ref20] Zhang Z.; Gernert U.; Gerhardt R. F.; Höhn E.-M.; Belder D.; Kneipp J. Catalysis by Metal Nanoparticles in a Plug-In Optofluidic Platform: Redox Reactions of p-Nitrobenzenethiol and p-Aminothiophenol. ACS Catal. 2018, 8, 2443–2449. 10.1021/acscatal.8b00101.

[ref21] Zhou B.; Ou W.; Shen J.; Zhao C.; Zhong J.; Du P.; Bian H.; Li P.; Yang L.; Lu J.; Li Y. Controlling Plasmon-Aided Reduction of p-Nitrothiophenol by Tuning the Illumination Wavelength. ACS Catal. 2021, 11, 14898–14905. 10.1021/acscatal.1c04091.

[ref22] Kafle B.; Poveda M.; Habteyes T. G. Surface Ligand-Mediated Plasmon-Driven Photochemical Reactions. J. Phys. Chem. Lett. 2017, 8, 890–894. 10.1021/acs.jpclett.7b00106. 28177626

[ref23] Zhang Z.; Li Y.; Frisch J.; Bär M.; Rappich J.; Kneipp J. In Situ Surface-Enhanced Raman Scattering Shows Ligand-Enhanced Hot Electron Harvesting on Silver, Gold, and Copper nanoparticles. J. Catal. 2020, 383, 153–159. 10.1016/j.jcat.2020.01.006.

[ref24] Zhang Z.; Merk V.; Hermanns A.; Unger W. E. S.; Kneipp J. Role of Metal Cations in Plasmon-Catalyzed Oxidation: A Case Study of p-Aminothiophenol Dimerization. ACS Catal. 2017, 7, 7803–7809. 10.1021/acscatal.7b02700.

[ref25] Yan X.; Wang L.; Tan X.; Tian B.; Zhang J. Surface-Enhanced Raman Spectroscopy Assisted by Radical Capturer for Tracking of Plasmon-Driven Redox Reaction. Sci. Rep. 2016, 6, 30193 10.1038/srep30193. 27444268 PMC4957100

[ref26] Sun J.-J.; Su H.-S.; Yue H.-L.; Huang S.-C.; Huang T.-X.; Hu S.; Sartin M. M.; Cheng J.; Ren B. Role of Adsorption Orientation in Surface Plasmon-Driven Coupling Reactions Studied by Tip-Enhanced Raman Spectroscopy. J. Phys. Chem. Lett. 2019, 10, 2306–2312. 10.1021/acs.jpclett.9b00203. 31013094

[ref27] Cai Z.-F.; Merino J. P.; Fang W.; Kumar N.; Richardson J. O.; De Feyter S.; Zenobi R. Molecular-Level Insights on Reactive Arrangement in On-Surface Photocatalytic Coupling Reactions Using Tip-Enhanced Raman Spectroscopy. J. Am. Chem. Soc. 2022, 144, 538–546. 10.1021/jacs.1c11263. 34941263

[ref28] Stöckle R. M.; Deckert V.; Fokas C.; Zenobi R. Controlled Formation of Isolated Silver Islands for Surface-Enhanced Raman Scattering. Appl. Spectrosc. 2000, 54, 1577–1583. 10.1366/0003702001948826.

[ref29] Feofanov A.; Ianoul A.; Kryukov E.; Maskevich S.; Vasiliuk G.; Kivach L.; Nabiev I. Nondisturbing and Stable SERS-Active Substrates with Increased Contribution of Long-Range Component of Raman Enhancement Created by High-Temperature Annealing of Thick Metal Films. Anal. Chem. 1997, 69, 3731–3740. 10.1021/ac970304c.

[ref30] Deckert-Gaudig T.; Kurouski D.; Hedegaard M.; Singh P.; Lednev I. K.; Deckert V. Spatially resolved spectroscopic differentiation of hydrophilic and hydrophobic domains on individual insulin amyloid fibrils. Sci. Rep. 2016, 6, 33575 10.1038/srep33575. 27650589 PMC5030623

[ref31] Yao X.; Höppener C.; Schneidewind H.; Hoeppener S.; Tang Z.; Buchholz A.; König A.; Mogavero S.; Diegel M.; Dellith J.; Turchanin A.; Plass W.; Hube B.; Deckert V. Targeted Suppression of Peptide Degradation in Ag-Based Surface-Enhanced Raman Spectra by Depletion of Hot Carriers. Small 2022, 18, 2205080 10.1002/smll.202205080. 36344458

[ref32] Rani K. K.; Devasenathipathy R.; Wang J.-Z.; Hui X.-Y.; Lin J.-D.; Zhang Y.-M.; Zhao L.-B.; Zhou J.-Z.; Wu D.-Y.; Tian Z.-Q. Plasmonic Photoelectrochemical Reactions on Noble Metal Electrodes of Nanostructures. Curr. Opin. Electrochem. 2022, 34, 100985 10.1016/j.coelec.2022.100985.

[ref33] Okotrub K. A.; Shamaeva D. V.; Surovtsev N. V. Raman spectra of Deuterated Hydrocarbons for Labeling Applications. J. Raman Spectrosc. 2022, 53, 297–309. 10.1002/jrs.6279.

[ref34] Tian Z.-Q.; Ren B.; Li J.-F.; Yang Z.-L. Expanding Generality of Surface-Enhanced Raman Spectroscopy with Borrowing SERS Activity Strategy. Chem. Commun. 2007, 3514–3534. 10.1039/b616986d. 18080535

[ref35] Amorim da Costa A. M.; Marques M. P. M.; de Carvalho L. A. E. B. Raman Spectra of Putrescine, Spermidine and Spermine Polyamines and Their N-Deuterated and N-Ionized Derivatives. J. Raman Spectrosc. 2003, 34, 357–366. 10.1002/jrs.1001.

[ref36] Zhao L.-B.; Zhang M.; Huang Y.-F.; Williams C.-T.; Wu D.-Y.; Ren B.; Tian Z.-Q. Theoretical Study of Plasmon-Enhanced Surface Catalytic Coupling Reactions of Aromatic Amines and Nitro Compounds. J. Phys. Chem. Lett. 2014, 5, 1259–1266. 10.1021/jz5003346. 26274481

[ref37] Zhao L.-B.; Chen J.-L.; Zhang M.; Wu D.-Y.; Tian Z.-Q. Theoretical Study on Electroreduction of p-Nitrothiophenol on Silver and Gold Electrode Surfaces. J. Phys. Chem. C 2015, 119, 4949–4958. 10.1021/jp512957c.

[ref38] Li Z.; Wang R.; Kurouski D. Nanoscale Photocatalytic Activity of Gold and Gold–Palladium Nanostructures Revealed by Tip-Enhanced Raman Spectroscopy. J. Phys. Chem. Lett. 2020, 11, 5531–5537. 10.1021/acs.jpclett.0c01631. 32568534

[ref39] Howard A. A.; Tschumper G. S.; Hammer N. I. Effects of Hydrogen Bonding on Vibrational Normal Modes of Pyrimidine. J. Phys. Chem. A 2010, 114, 6803–6810. 10.1021/jp101267w. 20527867

[ref40] Ling Y.; Xie W.-C.; Liu G.-K.; Yan R.-W.; Wu D.-Y.; Tang J. The Discovery of the Hydrogen Bond from p-Nitrothiophenol by Raman Spectroscopy: Guideline for the Thioalcohol Molecule Recognition Tool. Sci. Rep. 2016, 6, 31981 10.1038/srep31981. 27659311 PMC5034243

[ref41] Singh A.; Gangopadhyay D.; Nandi R.; Sharma P.; Singh R. K. Raman Signatures of Strong and Weak Hydrogen Bonds in Binary Mixtures of Phenol with Acetonitrile, Benzene and Orthodichlorobenzene. J. Raman Spectrosc. 2016, 47, 712–719. 10.1002/jrs.4880.

[ref42] Clark T.; Heske J.; Kühne T. D. Opposing Electronic and Nuclear Quantum Effects on Hydrogen Bonds in H_2_O and D_2_O. ChemPhysChem 2019, 20, 2461–2465. 10.1002/cphc.201900839. 31449714 PMC6790677

[ref43] Tsutsumi H.; Furumoto S.; Morita M.; Matsuda Y. Electrochemical Behavior of a 4-Nitrothiophenol Modified Electrode Prepared by the Self-Assembly Method. J. Colloid Interface Sci. 1995, 171, 505–511. 10.1006/jcis.1995.1209.

[ref44] Holze R. Spectroelectrochemical study of p-nitroso-N,N-dimethylaniline adsorbed on a gold electrode. Vib. Spectrosc. 1993, 4, 175–184. 10.1016/0924-2031(93)87036-S.

[ref45] Latorre F.; Kupfer S.; Bocklitz T.; Kinzel D.; Trautmann S.; Gräfe S.; Deckert V. Spatial resolution of tip-enhanced Raman spectroscopy – DFT assessment of the chemical effect. Nanoscale 2016, 8, 10229–10239. 10.1039/C6NR00093B. 27123952

[ref46] Fiederling K.; Abasifard M.; Richter M.; Deckert V.; Gräfe S.; Kupfer S. The chemical effect goes resonant - a full quantum mechanical approach on TERS. Nanoscale 2020, 12 (11), 6346–6359. 10.1039/C9NR09814C. 32134418

[ref47] Rodriguez R. D.; Villagómez C. J.; Khodadadi A.; Kupfer S.; Averkiev A.; Dedelaite L.; Tang F.; Khaywah M. Y.; Kolchuzhin V.; Ramanavicius A.; Adam P.-M.; Gräfe S.; Sheremet E. Chemical Enhancement vs Molecule–Substrate Geometry in Plasmon-Enhanced Spectroscopy. ACS Photonics 2021, 8 (8), 2243–2255. 10.1021/acsphotonics.1c00001.

[ref48] Fiederling K.; Abasifard M.; Richter M.; Deckert V.; Kupfer S.; Grafe S. A Full Quantum Mechanical Approach Assessing the Chemical and Electromagnetic Effect in TERS. ACS Nano 2023, 17 (14), 13137–13146. 10.1021/acsnano.2c11855. 37429582 PMC10373516

[ref49] Li Z.; Ehtesabi S.; Gojare S.; Richter M.; Kupfer S.; Gräfe S.; Kurouski D. Plasmon-Determined Selectivity in Photocatalytic Transformations on Gold and Gold–Palladium Nanostructures. ACS Photonics 2023, 10 (9), 3390–3400. 10.1021/acsphotonics.3c00893. PMC10863388 38356782

[ref50] Li Z.; Rigor J.; Ehtesabi S.; Gojare S.; Kupfer S.; Gräfe S.; Large N.; Kurouski D. Role of Plasmonic Antenna in Hot Carrier-Driven Reactions on Bimetallic Nanostructures. J. Phys. Chem. C 2023, 127 (46), 22635–22645. 10.1021/acs.jpcc.3c06520. PMC10863061 38357685

[ref51] Zhao L.-B.; Huang Y.-F.; Liu X.-M.; Anema J. R.; Wu D.-Y.; Ren B.; Tian Z.-Q. A DFT study on photoinduced surface catalytic coupling reactions on nanostructured silver: selective formation of azobenzene derivatives from para-substituted nitrobenzene and aniline. Phys. Chem. Chem. Phys. 2012, 14, 12919–12929. 10.1039/c2cp41502j. 22899166

[ref52] Cai Z.-F.; Merino J. P.; Fang W.; Kumar N.; Richardson J. O.; De Feyter S.; Zenobi R. Molecular-Level Insights on Reactive Arrangement in On-Surface Photocatalytic Coupling Reactions Using Tip-Enhanced Raman Spectroscopy. J. Am. Chem. Soc. 2022, 144 (1), 538–546. 10.1021/jacs.1c11263. 34941263

[ref53] Zhang Z.; Kneipp J. Mapping the Inhomogeneity in Plasmonic Catalysis on SupportedGold Nanoparticles Using Surface-Enhanced Raman ScatteringMicrospectroscopy. Anal. Chem. 2018, 90, 9199–9205. 10.1021/acs.analchem.8b01701. 29969010

