# Supplementary material for: Mechanism of Plasmon-Induced Catalysis of Thiolates and the Impact of Reaction Conditions
Source: J Am Chem Soc. 2024 Jan 26;146(5):3031–42. doi: 10.1021/jacs.3c09309 (PMC10859934; doi:10.1021/jacs.3c09309)
Supplement: Supplementary file 1 — ja3c09309_si_001.pdf [file ja3c09309_si_001.pdf]

Supporting Information 2

Mechanism of Plasmon-Induced Catalysis of Thiolates and the Impact of Reaction Conditions

Xiaobin Yao[1,2], Sadaf Ehtesabi[2], Christiane Höppener[1,2], Tanja Deckert-Gaudig[1,2], Henrik Schneidewind[1], Stephan Kupfer[2], Stefanie Gräfe[2,3], Volker Deckert[1,2]

1. Leibniz Institute of Photonic Technology (IPHT), Albert-Einstein-Str. 9, 07745 Jena, Germany

2. Institute of Physical Chemistry (IPC) and Abbe Center of Photonics, Friedrich Schiller University Jena, Helmholtzweg 4, 07743 Jena, Germany

3. Fraunhofer Institute of Applied Optics and Precision Engineering, Albert-Einstein-Str. 7, 07745 Jena, Germany

Corresponding author: volker.deckert@leibniz-ipht.de

Computational Details

Quantum chemical simulations were performed in order to elucidate the mechanism underlying the plasmon-induced conversion of the 4-aminothiophenol (ATP) and 4-nitrothiophenol (NTP) into 4,4'-dimercaptoazobenzene (DMAB). Therefore, all geometry optimizations for singlet ground states of ATP, NTP, DMAB and intermediates (A-D) surface-immobilized on an Ag cluster were performed at the density functional level of theory. Periodic DFT calculations were based on the projector-augmented wave (PAW) method employing optB88-vdW functional in a real-space grid of 0.2 Å resolution, implemented in the GPAW program package in cooperation with the ASE interface. The Ag slab is represented by a 4×4×3 fcc(111) cluster, resulting in 3 layers of 16 Ag atoms using an optimized lattice constant of 4.1884 Å. All molecules were chemically anchored via the strongly bonding sulfur atom of the thiol-moiety to the respective Ag cluster. The subsequent partial structural relaxation was performed employing two-dimensional periodic boundary conditions (x- and y-direction) while the second and third layers of the Ag slab were frozen to reduce computational costs.

In the case of the immobilized substrates comprising merely one aromatic ring, several conformations are investigated, e.g. with respect to the relative orientation of the substituents with respect to the metallic surface. In addition, we identify a pronounced impact on the orientation with respect to the surface coverage. Hence, in case of high surface coverage, where in addition to the i) strong chemical interaction arising from the sulfur-metal bond,

ii) weaker substrate-metal interactions based on physisorption, as well as on iii) dispersive interactions between the substrate molecules, favor configurations with perpendicular orientation of the aromatic planes relative to the metal surface. In order to evaluate the dispersive intermolecular interactions among neighboring surface-immobilized substrate molecules in more detail, two conformers with parallel as well as with antiparallel orientation were assessed. The simulations of intermediate B reveal that parallel structures are approximately 0.15 eV more favorable in comparison to antiparallel orientations. Consequently, parallel structures were employed in all calculations, including two molecules on an Ag cluster. As the dimerization, yielding DMAB, is restricted to neighboring molecules, the structure of this product features a more pronounced degree of rigidity, e.g. the strong metal-sulfur bond of the monomers allows exclusively the formation of the cis-isomer. Therefore, only cis-isomer was investigated for the DMAB molecule.

Subsequently, non-periodic time-dependent DFT (TDDFT) simulations were performed using the Gaussian 16 program. The electronic nature of light-driven processes in resonance upon 532-nm photoexcitation (2.33 eV) of the plasmonic hybrid system(s) were investigated at the CAM-B3LYP/def2-svp level of theory. In particular, the photoinduced redox chemistry between the Ag cluster and the respective substrate (ATP, NTP, DMAB, intermediates) were assessed. The electronic character, i.e. local excitation of the substrate as well as charge transfer excitation between the substrate and the Ag cluster, was evaluated based on charge-density differences (CDDs). See Tables S2-S8 for details on the excited state properties for the bright singlet excitations in the range of 1.33 to 3.33 eV.

(The numbers of Figures and tables are continued to those of Supporting Information 1.)

Table S2: Charge density differences (CDDs) illustrating the nature of the low-lying bright excitations of NTP. Charge transfer takes place from red to blue.

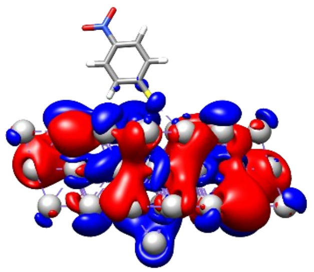<br>State 44<br>Energy: 1.491 eV<br>Osc.: 0.014	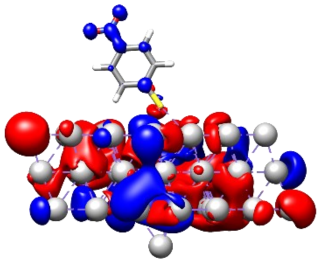<br>State 51<br>Energy: 1.587 eV<br>Osc.: 0.012	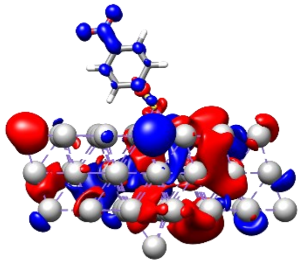<br>State 52<br>Energy: 1.627 eV<br>Osc.: 0.010	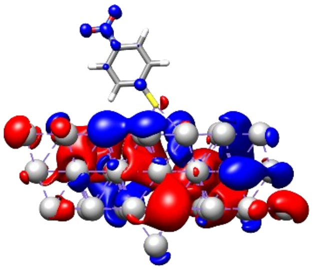<br>State 53<br>Energy: 1.640 eV<br>Osc.: 0.015
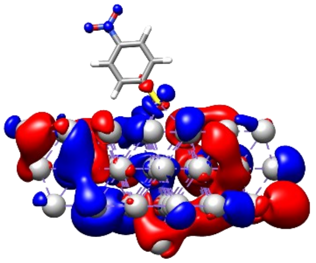<br>State 62<br>Energy: 1.814 eV<br>Osc.: 0.010	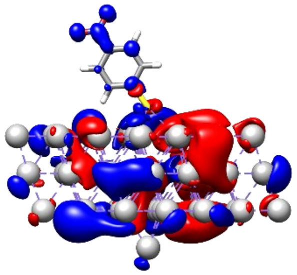<br>State 65<br>Energy: 1.846 eV<br>Osc.: 0.024	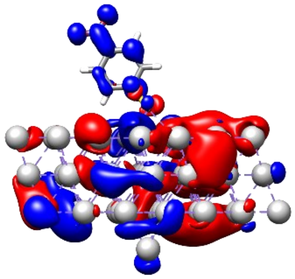<br>State 66<br>Energy: 1.865 eV<br>Osc.: 0.012	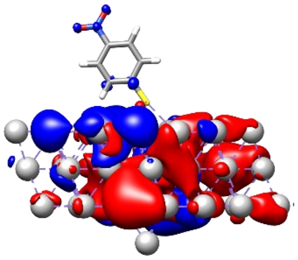<br>State 73<br>Energy: 1.933 eV<br>Osc.: 0.012
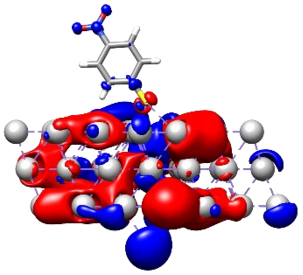<br>State 84<br>Energy: 2.084 eV<br>Osc.: 0.016	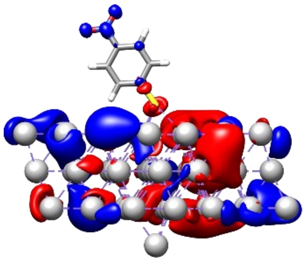<br>State 87<br>Energy: 2.132 eV<br>Osc.: 0.016	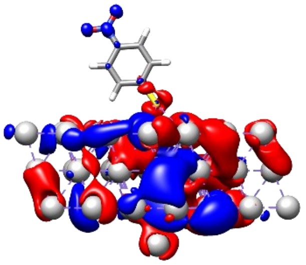<br>State 89<br>Energy: 2.160 eV<br>Osc.: 0.010	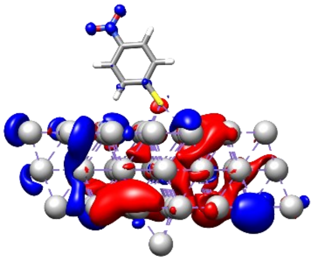<br>State 90<br>Energy: 2.171 eV<br>Osc.: 0.017

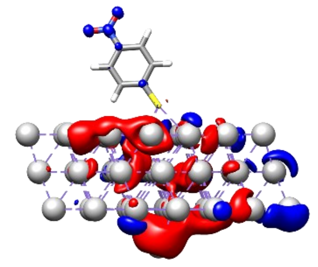

State 99
Energy: 2.280 eV
Osc.: 0.014

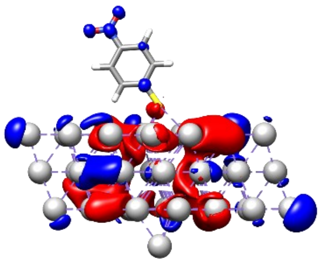

State 102
Energy: 2.302 eV
Osc.: 0.013

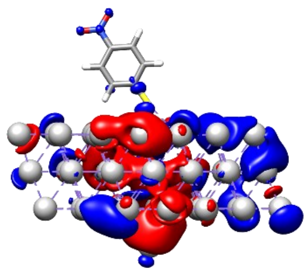

State 103
Energy: 2.313 eV
Osc.: 0.010

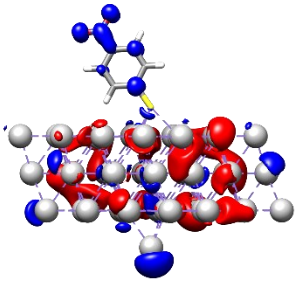

State 105
Energy: 2.334 eV
Osc.: 0.010

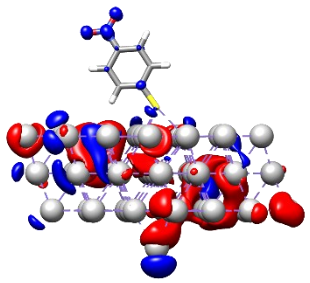

State 106
Energy: 2.357 eV
Osc.: 0.021

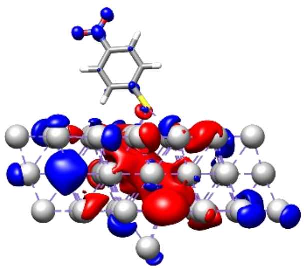

State 109
Energy: 2.383 eV
Osc.: 0.047

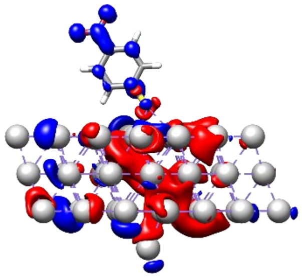

State 110
Energy: 2.398 eV
Osc.: 0.033

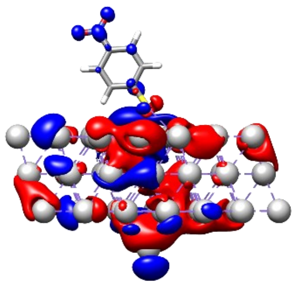

State 111
Energy: 2.404 eV
Osc.: 0.038

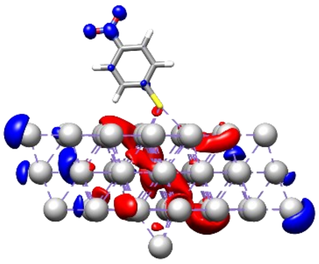

State 112
Energy: 2.433 eV
Osc.: 0.081

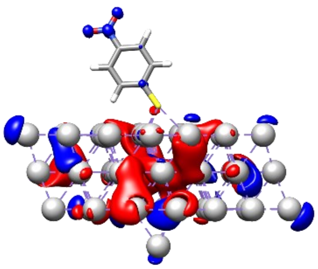

State 113
Energy: 2.447 eV
Osc.: 0.031

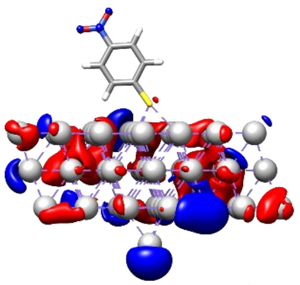

State 114
Energy: 2.452 eV
Osc.: 0.021

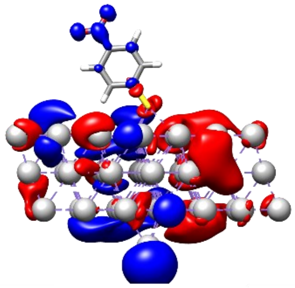

State 115
Energy: 2.455 eV
Osc.: 0.010

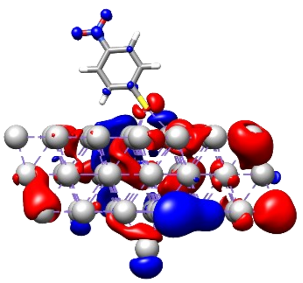

State 118
Energy: 2.488 eV
Osc.: 0.027

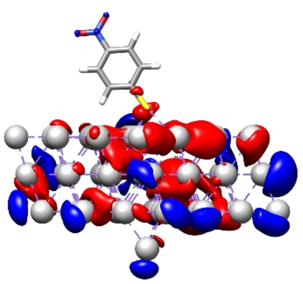

State 119
Energy: 2.498 eV
Osc.: 0.016

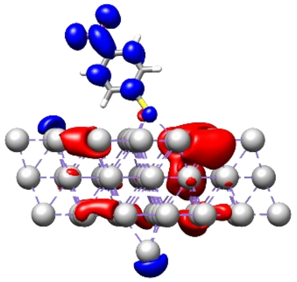

State 123
Energy: 2.530 eV
Osc.: 0.033

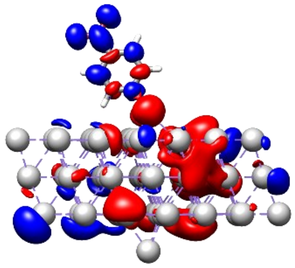

State 128
Energy: 2.580 eV
Osc.: 0.045

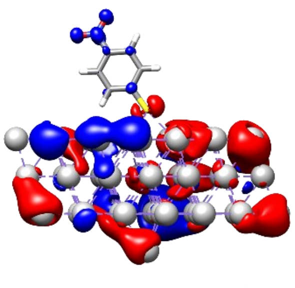

State 129
Energy: 2.589 eV
Osc.: 0.049

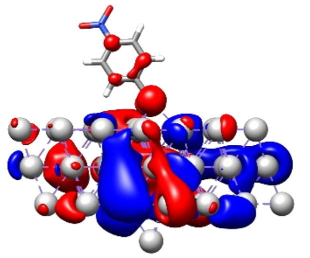

State 131
Energy: 2.600 eV
Osc.: 0.016

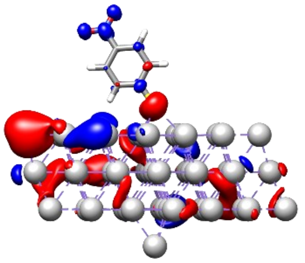

State 133
Energy: 2.624 eV
Osc.: 0.072

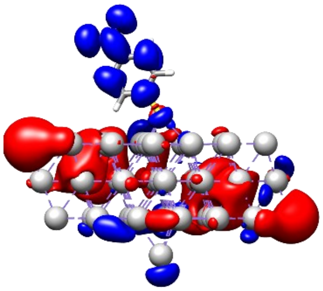

State 134
Energy: 2.628 eV
Osc.: 0.039

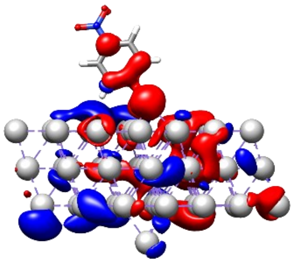

State 136
Energy: 2.638 eV
Osc.: 0.020

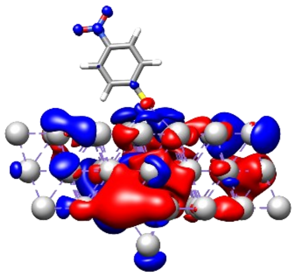

State 137
Energy: 2.651 eV
Osc.: 0.025

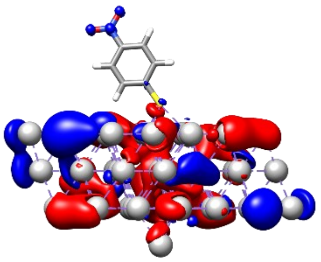

State 138
Energy: 2.654 eV
Osc.: 0.042

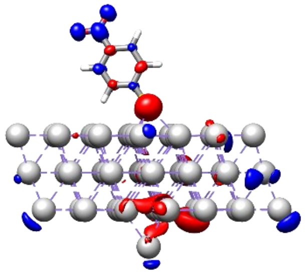

State 140
Energy: 2.667 eV
Osc.: 0.030

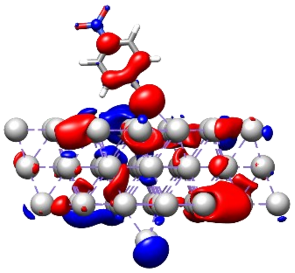

State 141
Energy: 2.671 eV
Osc.: 0.056

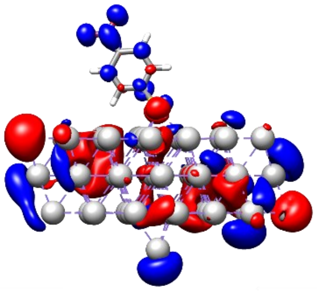

State 142
Energy: 2.686 eV
Osc.: 0.021

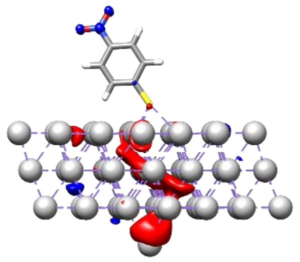

State 143
Energy: 2.696 eV
Osc.: 0.015

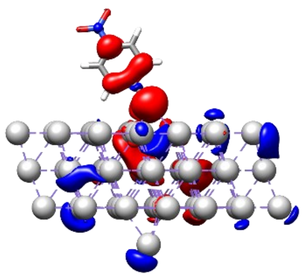

State 144
Energy: 2.701 eV
Osc.: 0.014

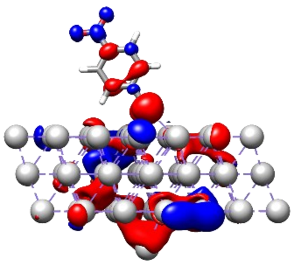

State 147
Energy: 2.735 eV
Osc.: 0.086

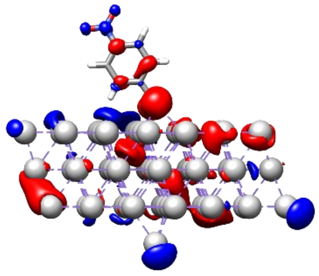

State 148
Energy: 2.742 eV
Osc.: 0.035

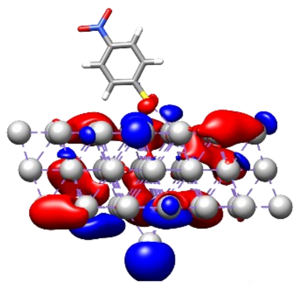

State 150
Energy: 2.751 eV
Osc.: 0.012

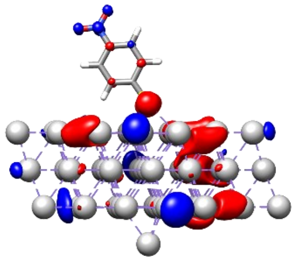

State 152
Energy: 2.776 eV
Osc.: 0.040

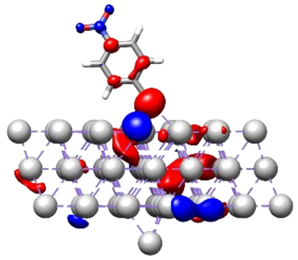

State 154
Energy: 2.795 eV
Osc.: 0.015

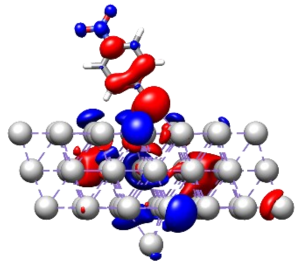

State 155
Energy: 2.802 eV
Osc.: 0.022

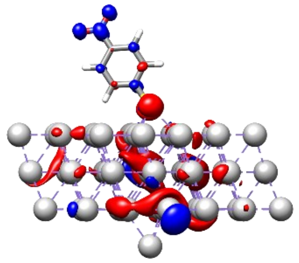

State 156
Energy: 2.811 eV
Osc.: 0.090

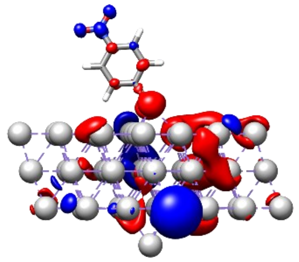

State 157
Energy: 2.817 eV
Osc.: 0.023

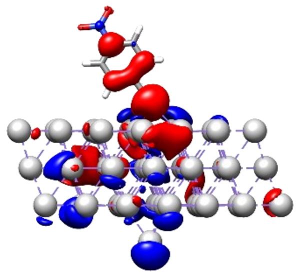

State 158
Energy: 2.830 eV
Osc.: 0.034

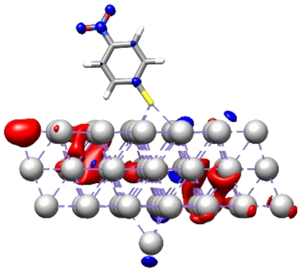

State 160
Energy: 2.840 eV
Osc.: 0.073

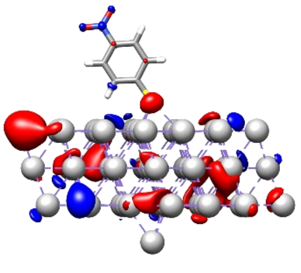

State 162
Energy: 2.855 eV
Osc.: 0.097

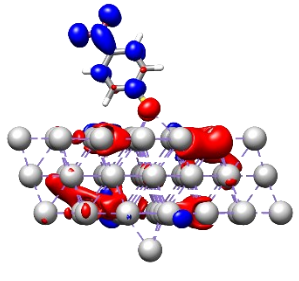

State 163
Energy: 2.863 eV
Osc.: 0.082

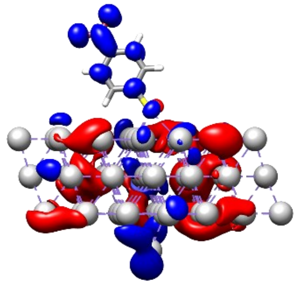

State 165
Energy: 2.885 eV
Osc.: 0.065

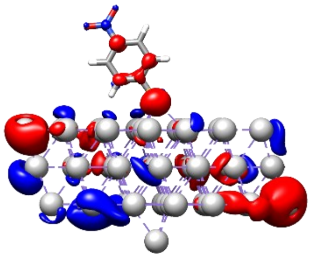

State 166
Energy: 2.894 eV
Osc.: 0.205

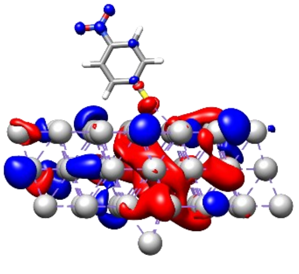

State 167
Energy: 2.903 eV
Osc.: 0.012

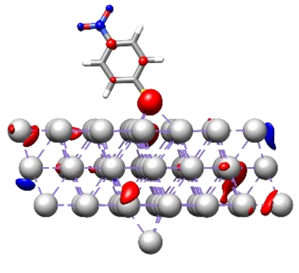

State 169
Energy: 2.917 eV
Osc.: 0.053

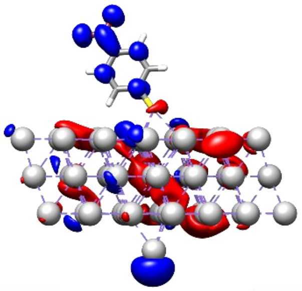

State 170
Energy: 2.923 eV
Osc.: 0.017

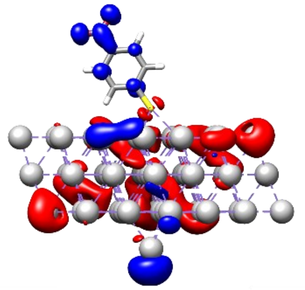

State 171
Energy: 2.928 eV
Osc.: 0.093

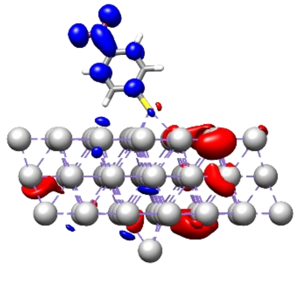

State 172
Energy: 2.939 eV
Osc.: 0.032

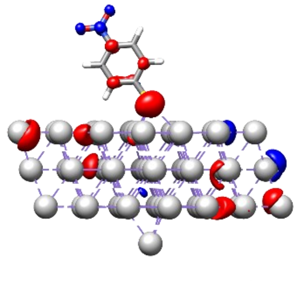

State 173
Energy: 2.947 eV
Osc.: 0.378

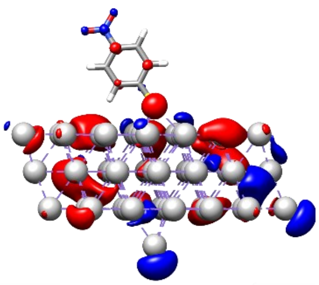
State 174
Energy: 2.952 eV
Osc.: 0.043

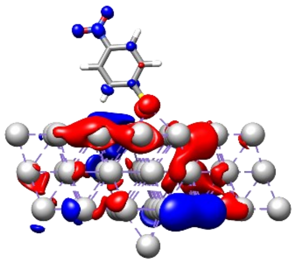
State 175
Energy: 2.955 eV
Osc.: 0.046

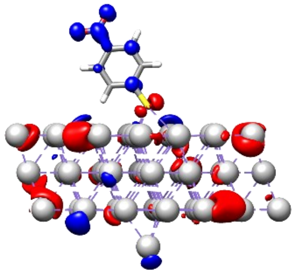
State 176
Energy: 2.965 eV
Osc.: 0.105

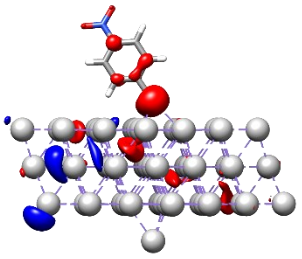
State 177
Energy: 2.973 eV
Osc.: 0.562

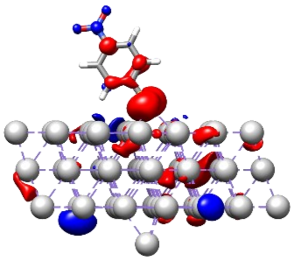
State 178
Energy: 2.985 eV
Osc.: 0.030

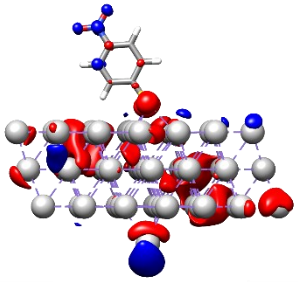
State 179
Energy: 2.992 eV
Osc.: 0.040

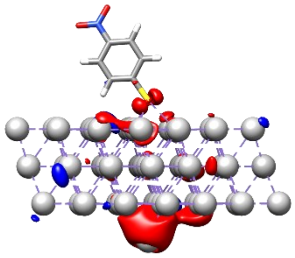
State 183
Energy: 3.021 eV
Osc.: 0.028

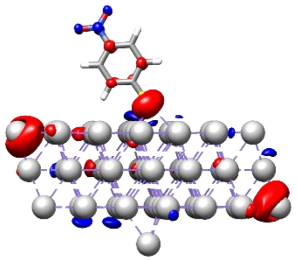
State 184
Energy: 3.032 eV
Osc.: 0.064

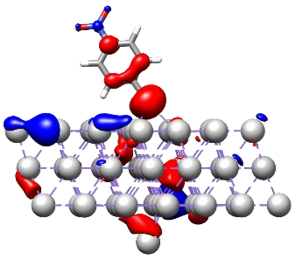
State 185
Energy: 3.036 eV
Osc.: 0.050

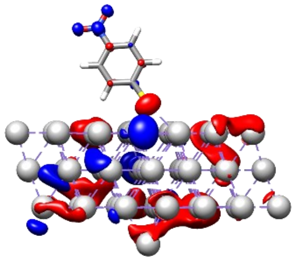
State 186
Energy: 3.043 eV
Osc.: 0.534

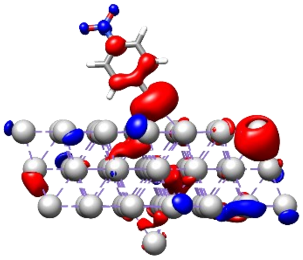
State 187
Energy: 3.045 eV
Osc.: 0.032

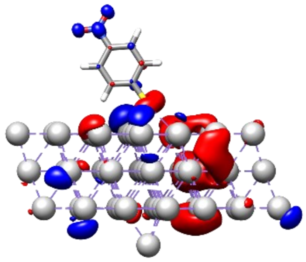
State 188
Energy: 3.060 eV
Osc.: 0.028

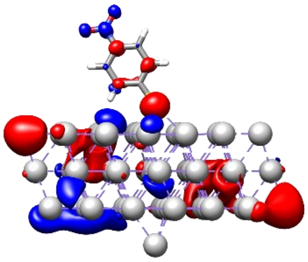
State 189
Energy: 3.063 eV
Osc.: 0.110

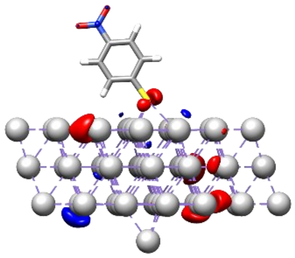
State 190
Energy: 3.071 eV
Osc.: 0.512

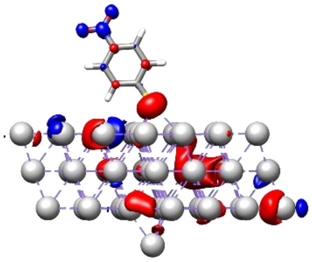
State 191
Energy: 3.080 eV
Osc.: 0.078

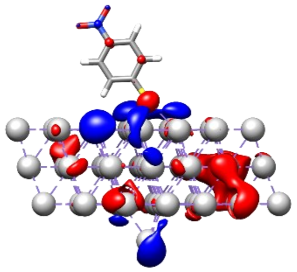
State 192
Energy: 3.088 eV
Osc.: 0.336

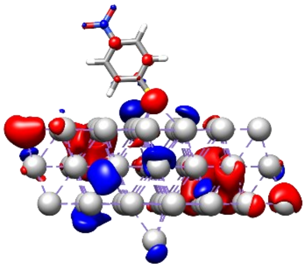

State 193
Energy: 3.101 eV
Osc.: 0.046

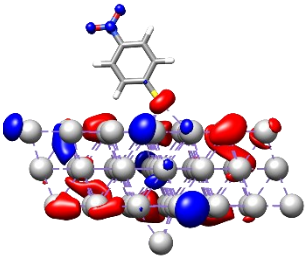

State 194
Energy: 3.106 eV
Osc.: 0.029

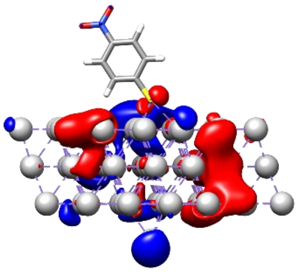

State 195
Energy: 3.114 eV
Osc.: 0.133

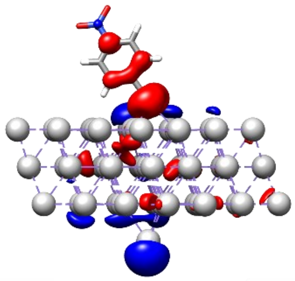

State 196
Energy: 3.118 eV
Osc.: 0.226

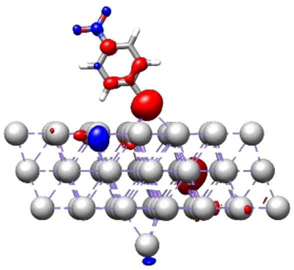

State 197
Energy: 3.124 eV
Osc.: 0.196

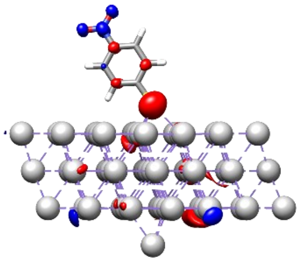

State 198
Energy: 3.126 eV
Osc.: 0.164

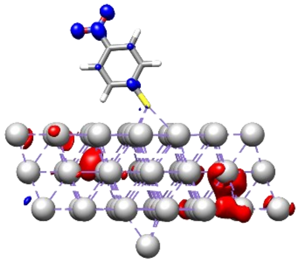

State 199
Energy: 3.137 eV
Osc.: 0.053

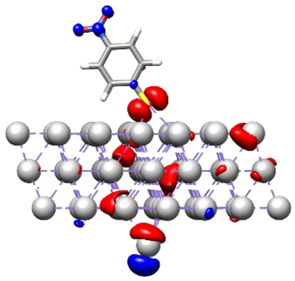

State 200
Energy: 3.138 eV
Osc.: 0.069

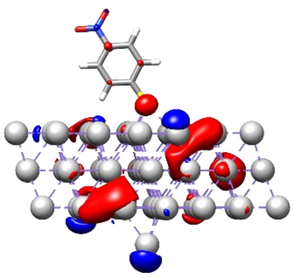

State 201
Energy: 3.148 eV
Osc.: 0.017

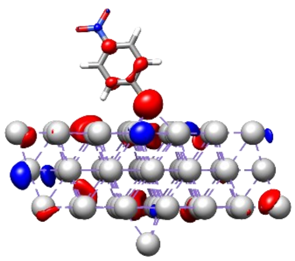

State 203
Energy: 3.162 eV
Osc.: 0.067

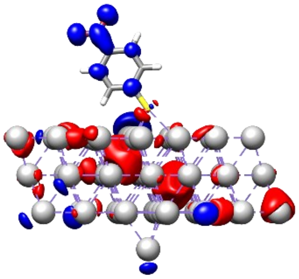

State 204
Energy: 3.170 eV
Osc.: 0.182

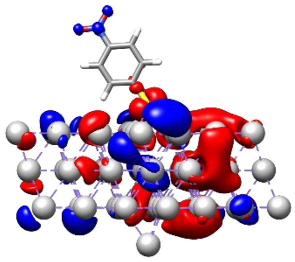

State 206
Energy: 3.182 eV
Osc.: 1.108

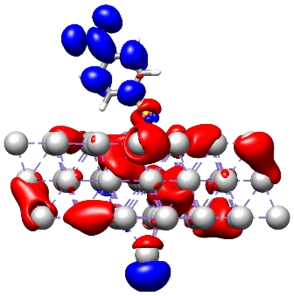

State 207
Energy: 3.185 eV
Osc.: 0.123

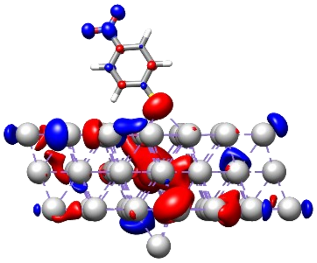

State 208
Energy: 3.193 eV
Osc.: 0.018

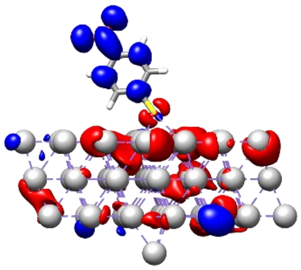

State 209
Energy: 3.202 eV
Osc.: 0.018

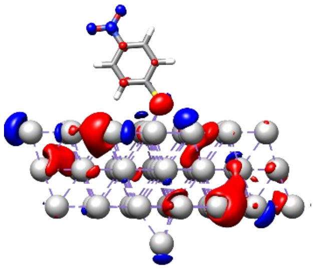

State 210
Energy: 3.207 eV
Osc.: 0.043

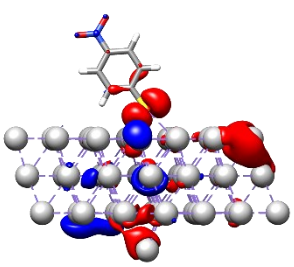<br>State 211<br>Energy: 3.216 eV<br>Osc.: 0.182	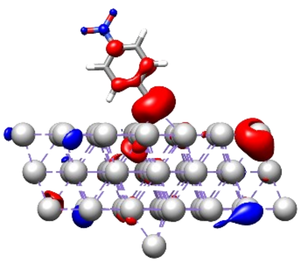<br>State 212<br>Energy: 3.219 eV<br>Osc.: 0.195	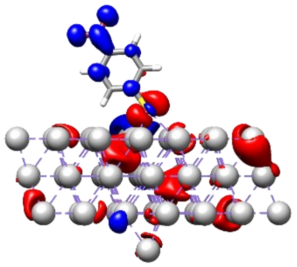<br>State 213<br>Energy: 3.226 eV<br>Osc.: 0.024	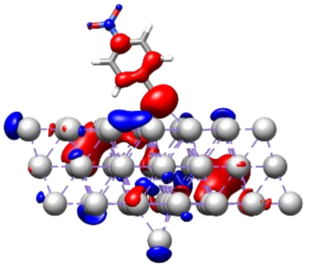<br>State 214<br>Energy: 3.232 eV<br>Osc.: 0.028
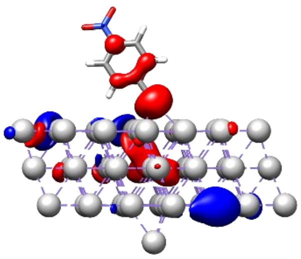<br>State 216<br>Energy: 3.248 eV<br>Osc.: 0.444	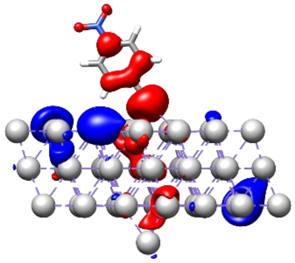<br>State 217<br>Energy: 3.255 eV<br>Osc.: 0.230	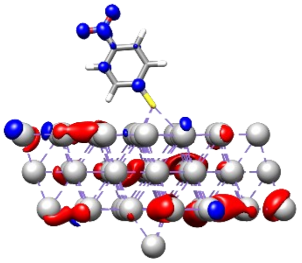<br>State 218<br>Energy: 3.265 eV<br>Osc.: 0.049	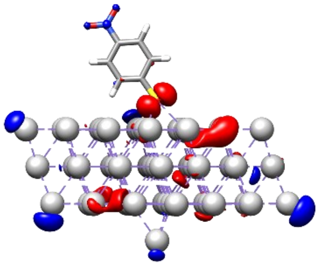<br>State 219<br>Energy: 3.277 eV<br>Osc.: 0.011
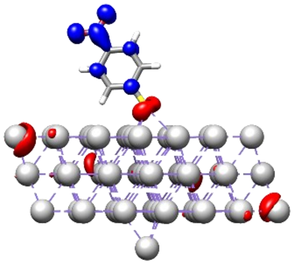<br>State 220<br>Energy: 3.290 eV<br>Osc.: 0.026	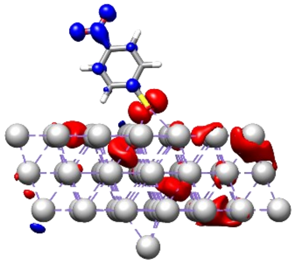<br>State 221<br>Energy: 3.291 eV<br>Osc.: 0.089	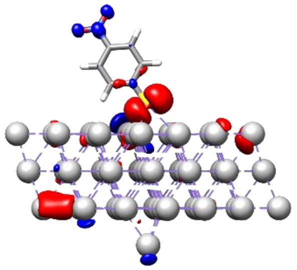<br>State 222<br>Energy: 3.304 eV<br>Osc.: 0.088	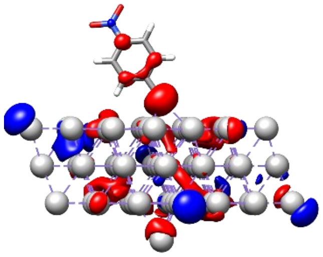<br>State 223<br>Energy: 3.309 eV<br>Osc.: 0.025

Table S3: Charge density differences (CDDs) illustrating the nature of the low-lying bright excitations of ATP. Charge transfer takes place from red to blue

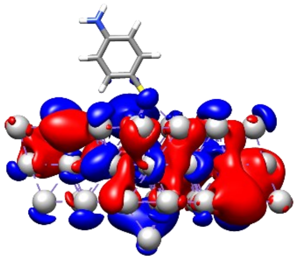<br>State 44<br>Energy: 1.502 eV<br>Osc.: 0.011	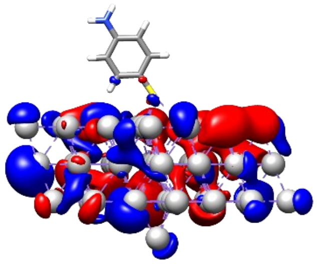<br>State 45<br>Energy: 1.528 eV<br>Osc.: 0.011	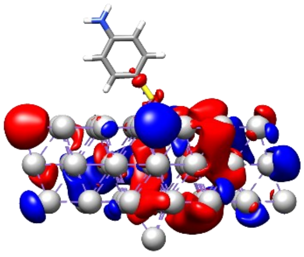<br>State 52<br>Energy: 1.635 eV<br>Osc.: 0.019	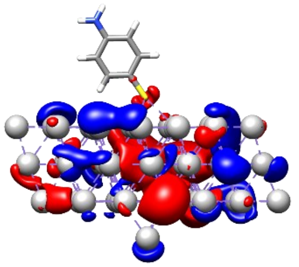<br>State 53<br>Energy: 1.636 eV<br>Osc.: 0.012
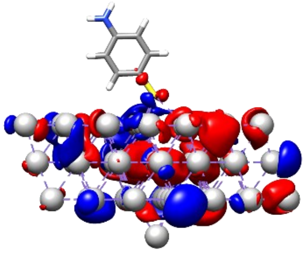<br>State 65<br>Energy: 1.852 eV<br>Osc.: 0.025	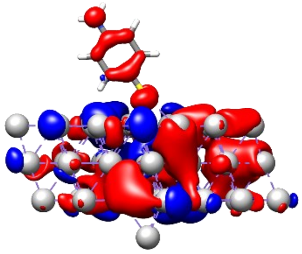<br>State 72<br>Energy: 1.926 eV<br>Osc.: 0.011	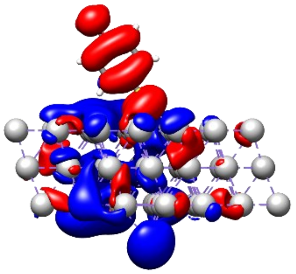<br>State 81<br>Energy: 2.053 eV<br>Osc.: 0.014	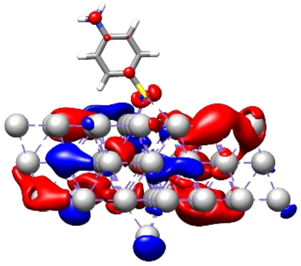<br>State 82<br>Energy: 2.055 eV<br>Osc.: 0.016
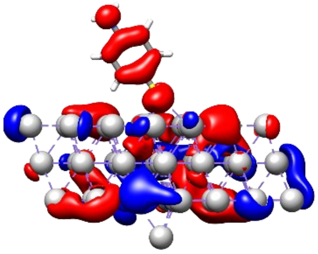<br>State 86<br>Energy: 2.104 eV<br>Osc.: 0.014	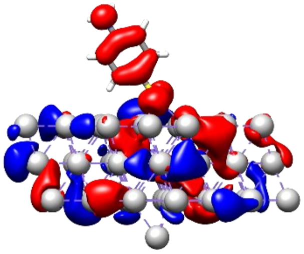<br>State 89<br>Energy: 2.146 eV<br>Osc.: 0.020	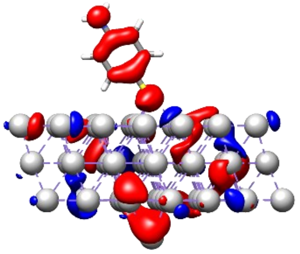<br>State 91<br>Energy: 2.168 eV<br>Osc.: 0.018	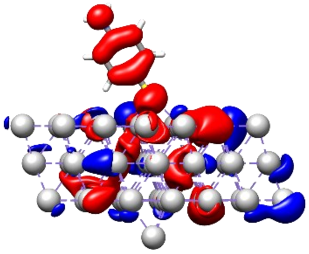<br>State 103<br>Energy: 2.289 eV<br>Osc.: 0.011

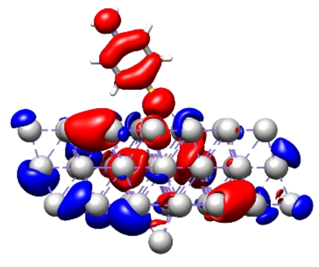<br>State 106<br>Energy: 2.310 eV<br>Osc.: 0.013	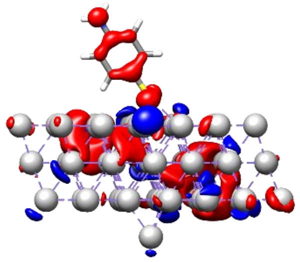<br>State 109<br>Energy: 2.356 eV<br>Osc.: 0.031	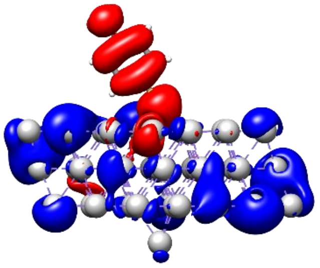<br>State 113<br>Energy: 2.402 eV<br>Osc.: 0.010	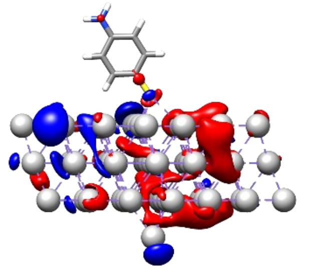<br>State 114<br>Energy: 2.405 eV<br>Osc.: 0.085
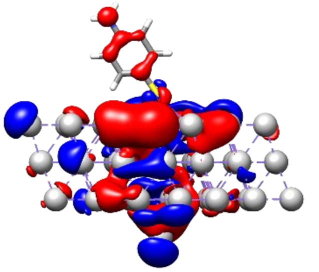<br>State 115<br>Energy: 2.412 eV<br>Osc.: 0.036	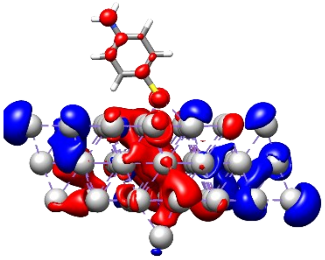<br>State 116<br>Energy: 2.435 eV<br>Osc.: 0.016	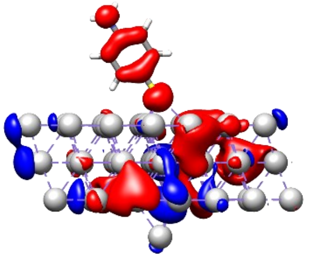<br>State 117<br>Energy: 2.444 eV<br>Osc.: 0.048	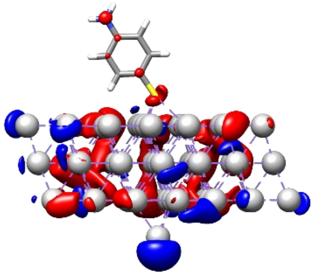<br>State 118<br>Energy: 2.452 eV<br>Osc.: 0.013
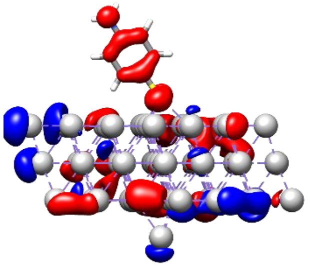<br>State 119<br>Energy: 2.453 eV<br>Osc.: 0.046	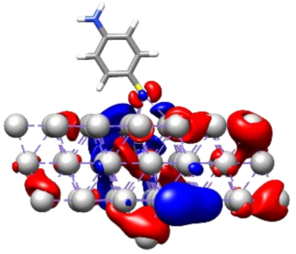<br>State 123<br>Energy: 2.493 eV<br>Osc.: 0.021	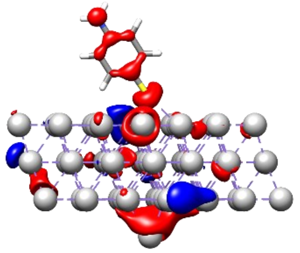<br>State 126<br>Energy: 2.517 eV<br>Osc.: 0.015	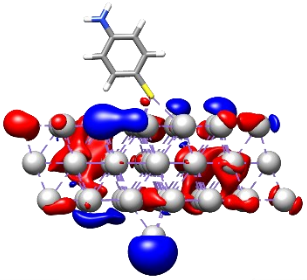<br>State 127<br>Energy: 2.526 eV<br>Osc.: 0.011
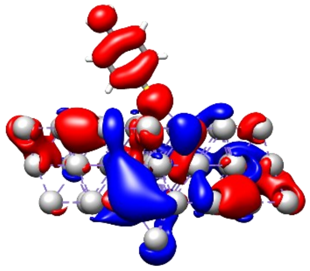<br>State 129<br>Energy: 2.560 eV<br>Osc.: 0.022	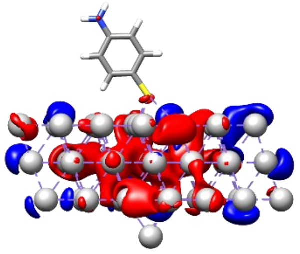<br>State 132<br>Energy: 2.582 eV<br>Osc.: 0.051	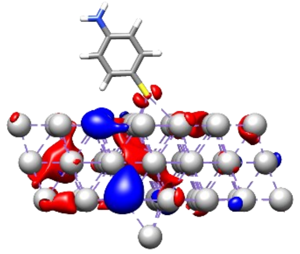<br>State 133<br>Energy: 2.589 eV<br>Osc.: 0.052	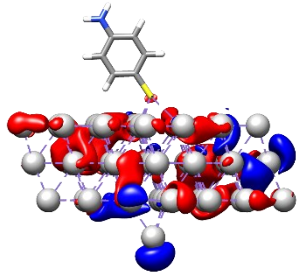<br>State 135<br>Energy: 2.606 eV<br>Osc.: 0.039

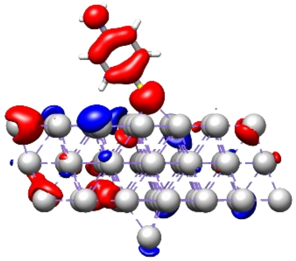<br>State 137<br>Energy: 2.625 eV<br>Osc.: 0.096	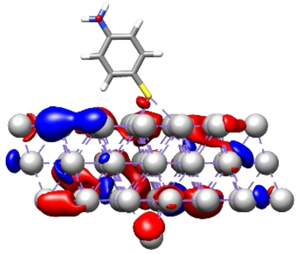<br>State 140<br>Energy: 2.648 eV<br>Osc.: 0.071	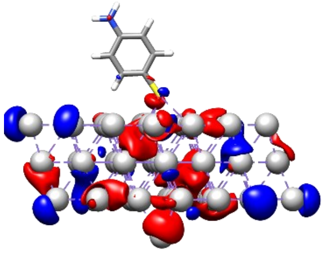<br>State 141<br>Energy: 2.652 eV<br>Osc.: 0.039	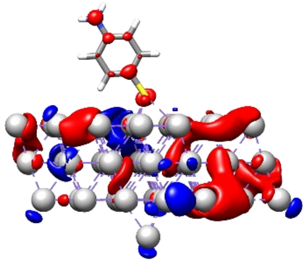<br>State 142<br>Energy: 2.657 eV<br>Osc.: 0.023
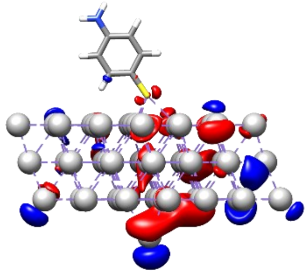<br>State 143<br>Energy: 2.661 eV<br>Osc.: 0.070	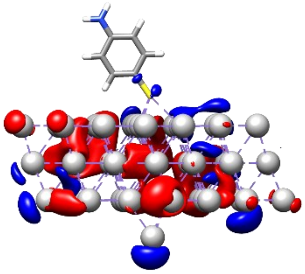<br>State 144<br>Energy: 2.676 eV<br>Osc.: 0.013	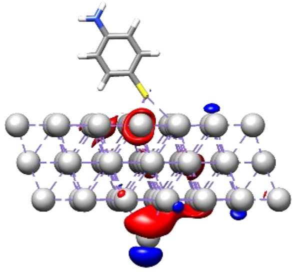<br>State 145<br>Energy: 2.692 eV<br>Osc.: 0.016	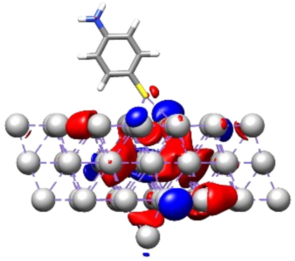<br>State 147<br>Energy: 2.708 eV<br>Osc.: 0.025
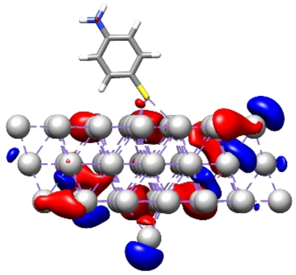<br>State 148<br>Energy: 2.722 eV<br>Osc.: 0.020	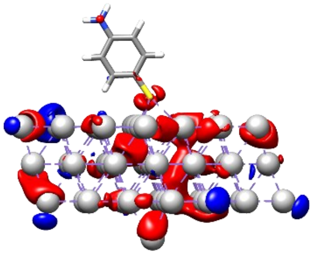<br>State 149<br>Energy: 2.729 eV<br>Osc.: 0.080	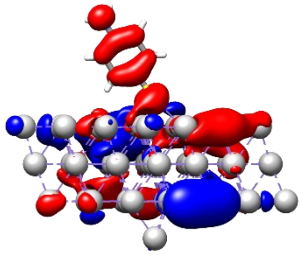<br>State 152<br>Energy: 2.763 eV<br>Osc.: 0.083	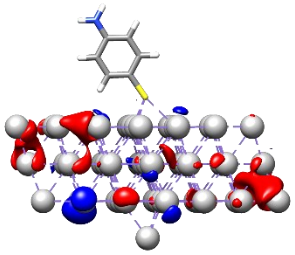<br>State 155<br>Energy: 2.780 eV<br>Osc.: 0.044
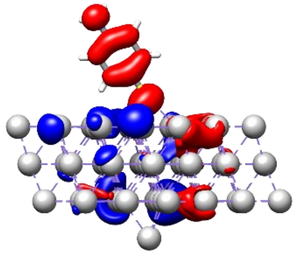<br>State 160<br>Energy: 2.829 eV<br>Osc.: 0.155	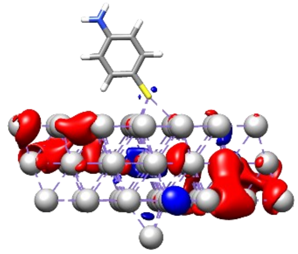<br>State 161<br>Energy: 2.836 eV<br>Osc.: 0.014	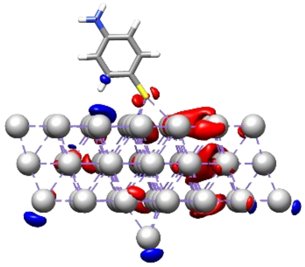<br>State 162<br>Energy: 2.844 eV<br>Osc.: 0.103	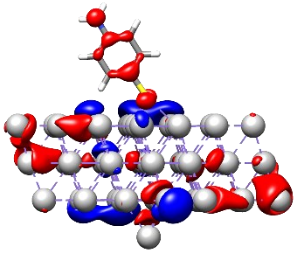<br>State 163<br>Energy: 2.855 eV<br>Osc.: 0.061

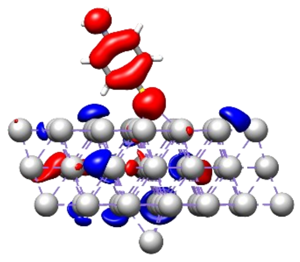

State 164
Energy: 2.863 eV
Osc.: 0.011

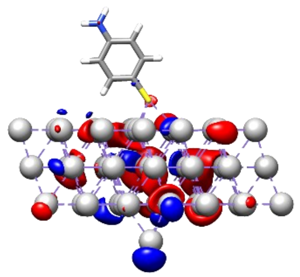

State 165
Energy: 2.882 eV
Osc.: 0.148

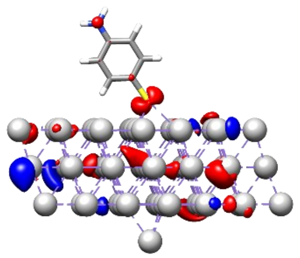

State 166
Energy: 2.886 eV
Osc.: 0.159

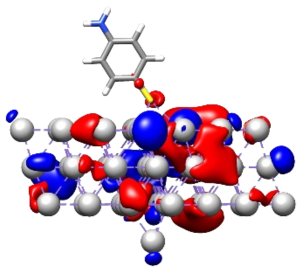

State 167
Energy: 2.895 eV
Osc.: 0.033

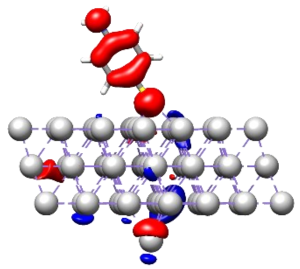

State 169
Energy: 2.908 eV
Osc.: 0.012

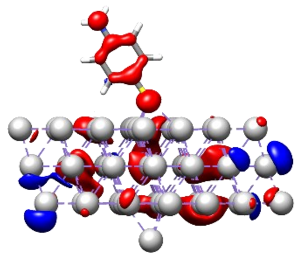

State 170
Energy: 2.915 eV
Osc.: 0.060

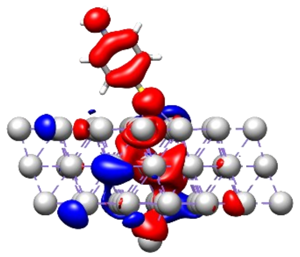

State 171
Energy: 2.920 eV
Osc.: 0.018

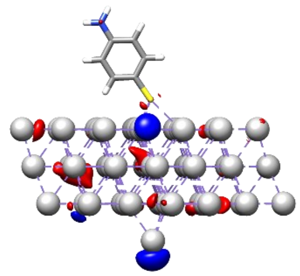

State 172
Energy: 2.927 eV
Osc.: 0.042

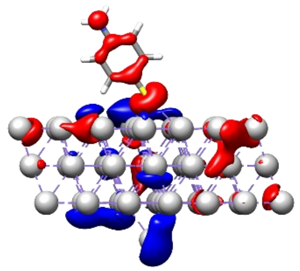

State 173
Energy: 2.931 eV
Osc.: 0.018

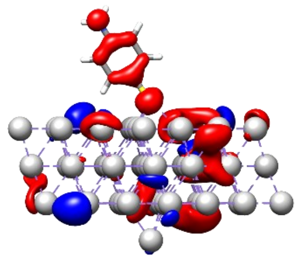

State 174
Energy: 2.940 eV
Osc.: 0.158

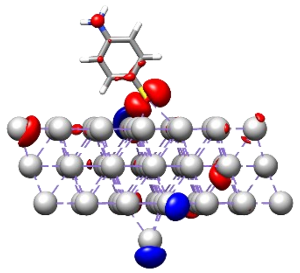

State 175
Energy: 2.942 eV
Osc.: 0.272

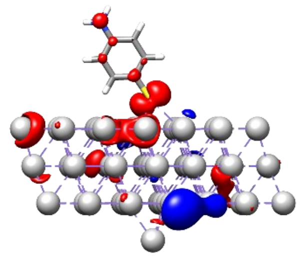

State 176
Energy: 2.952 eV
Osc.: 0.207

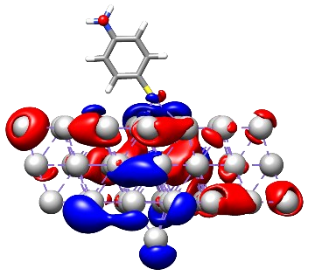

State 177
Energy: 2.965 eV
Osc.: 0.041

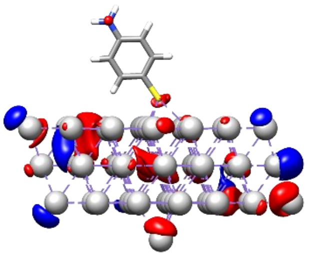

State 178
Energy: 2.972 eV
Osc.: 0.186

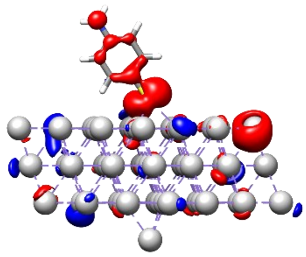

State 179
Energy: 2.974 eV
Osc.: 0.090

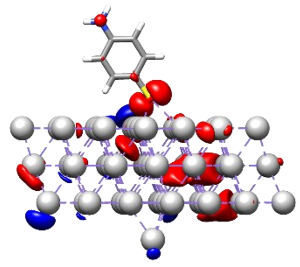

State 180
Energy: 2.992 eV
Osc.: 0.010

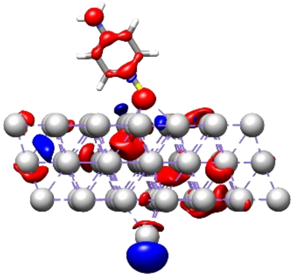	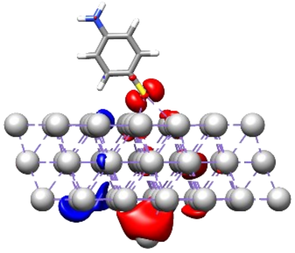	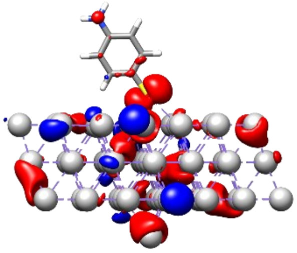	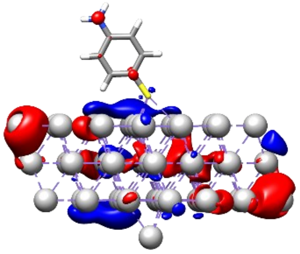
State 181<br>Energy: 2.994 eV<br>Osc.: 0.149	State 183<br>Energy: 3.007 eV<br>Osc.: 0.038	State 184<br>Energy: 3.017 eV<br>Osc.: 0.016	State 185<br>Energy: 3.033 eV<br>Osc.: 0.020
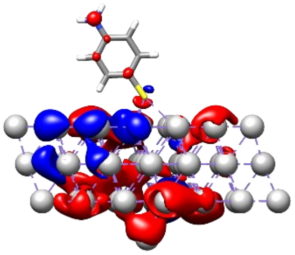	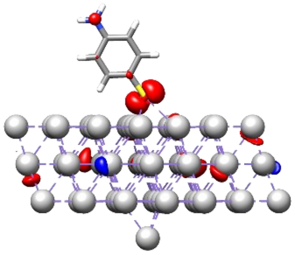	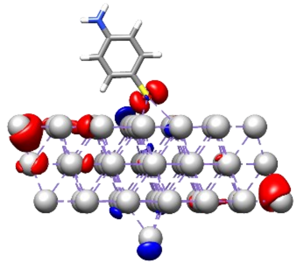	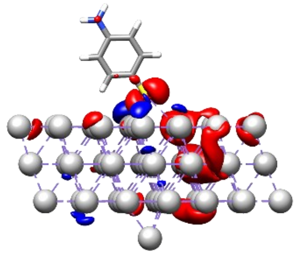
State 186<br>Energy: 3.039 eV<br>Osc.: 0.268	State 187<br>Energy: 3.044 eV<br>Osc.: 0.617	State 189<br>Energy: 3.063 eV<br>Osc.: 0.250	State 190<br>Energy: 3.067 eV<br>Osc.: 0.107
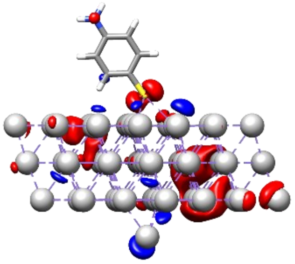	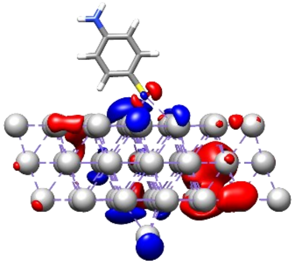	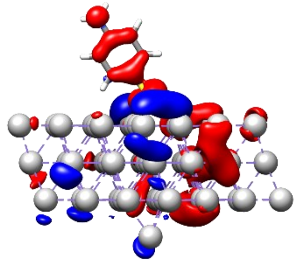	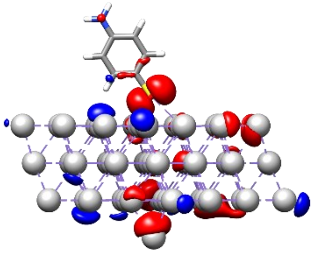
State 191<br>Energy: 3.073 eV<br>Osc.: 0.153	State 192<br>Energy: 3.080 eV<br>Osc.: 0.282	State 193<br>Energy: 3.085 eV<br>Osc.: 0.115	State 195<br>Energy: 3.098 eV<br>Osc.: 0.035
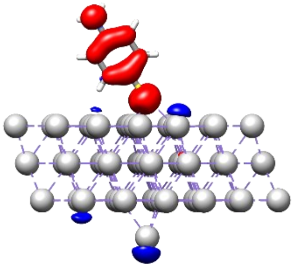	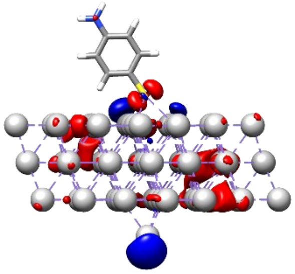	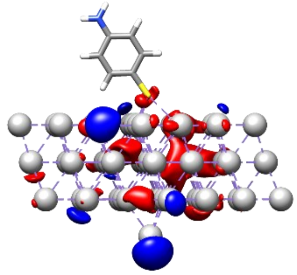	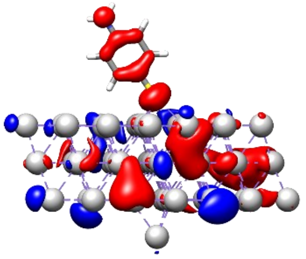
State 196<br>Energy: 3.103 eV<br>Osc.: 0.077	State 197<br>Energy: 3.111 eV<br>Osc.: 0.290	State 198<br>Energy: 3.113 eV<br>Osc.: 0.069	State 199<br>Energy: 3.124 eV<br>Osc.: 0.119

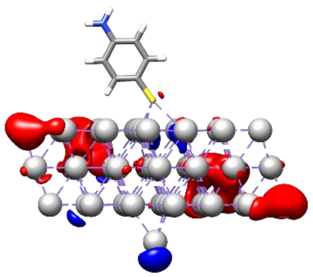<br>State 200<br>Energy: 3.130 eV<br>Osc.: 0.034	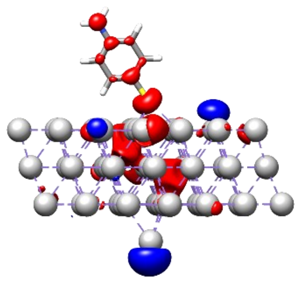<br>State 202<br>Energy: 3.151 eV<br>Osc.: 0.016	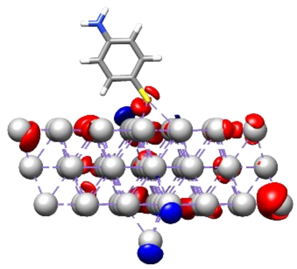<br>State 203<br>Energy: 3.154 eV<br>Osc.: 0.061	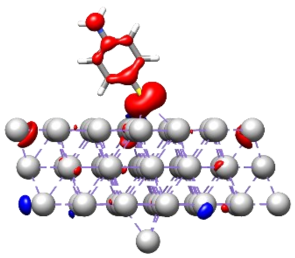<br>State 204<br>Energy: 3.161 eV<br>Osc.: 0.587
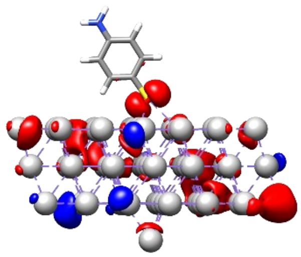<br>State 205<br>Energy: 3.168 eV<br>Osc.: 0.460	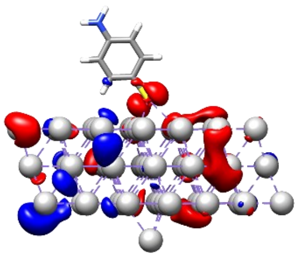<br>State 206<br>Energy: 3.177 eV<br>Osc.: 0.754	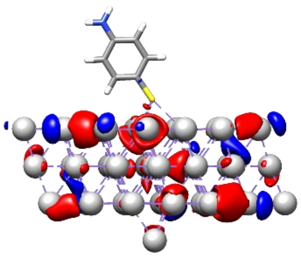<br>State 207<br>Energy: 3.179 eV<br>Osc.: 0.040	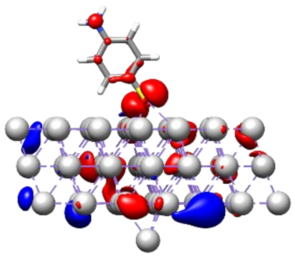<br>State 208<br>Energy: 3.185 eV<br>Osc.: 0.018
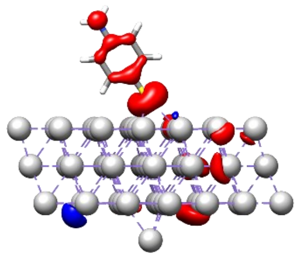<br>State 209<br>Energy: 3.196 eV<br>Osc.: 0.018	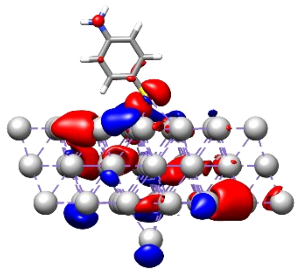<br>State 210<br>Energy: 3.202 eV<br>Osc.: 0.049	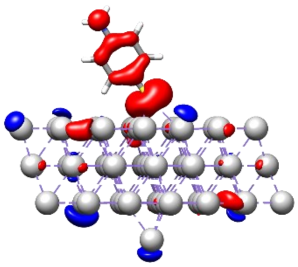<br>State 211<br>Energy: 3.208 eV<br>Osc.: 0.029	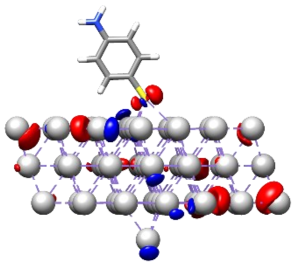<br>State 213<br>Energy: 3.214 eV<br>Osc.: 0.338
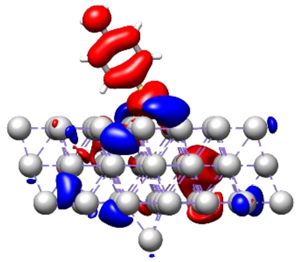<br>State 214<br>Energy: 3.229 eV<br>Osc.: 0.106	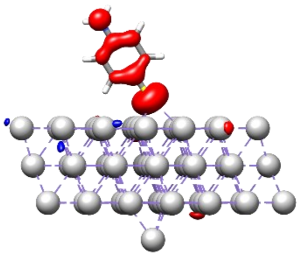<br>State 215<br>Energy: 3.240 eV<br>Osc.: 0.195	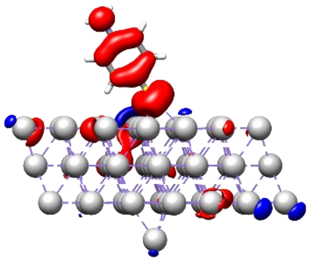<br>State 216<br>Energy: 3.244 eV<br>Osc.: 0.076	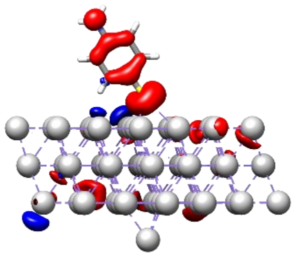<br>State 217<br>Energy: 3.249 eV<br>Osc.: 0.029

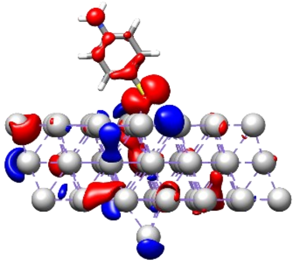<br>State 218<br>Energy: 3.257 eV<br>Osc.: 0.104	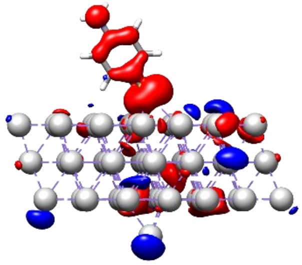<br>State 220<br>Energy: 3.282 eV<br>Osc.: 0.013	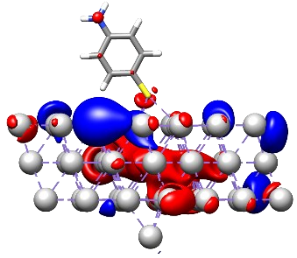<br>State 221<br>Energy: 3.286 eV<br>Osc.: 0.035	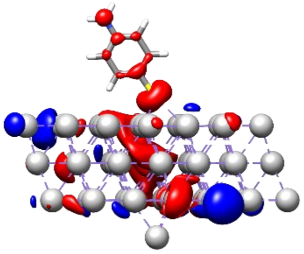<br>State 222<br>Energy: 3.293 eV<br>Osc.: 0.016
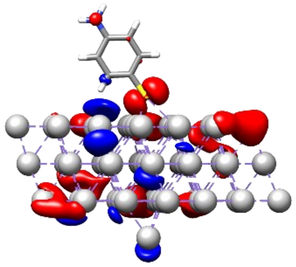<br>State 223<br>Energy: 3.298 eV<br>Osc.: 0.008	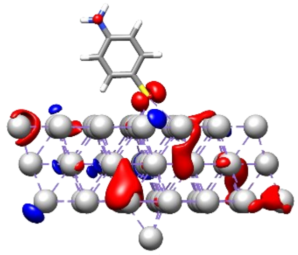<br>State 224<br>Energy: 3.301 eV<br>Osc.: 0.298	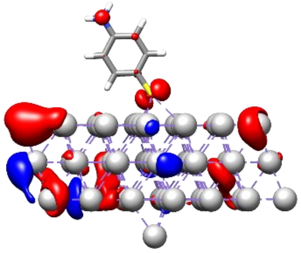<br>State 225<br>Energy: 3.307 eV<br>Osc.: 0.013	

Table S4: Charge density differences (CDDs) illustrating the nature of the low-lying bright excitations of DMAB. Charge transfer takes place from red to blue

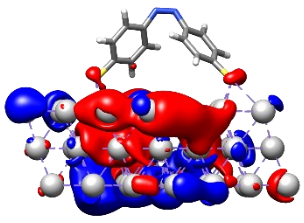<br>State 48<br>Energy: 1.652 eV<br>Osc.: 0.019	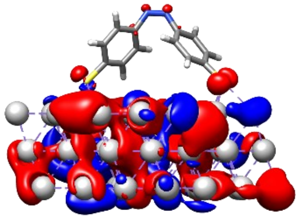<br>State 52<br>Energy: 1.705 eV<br>Osc.: 0.016	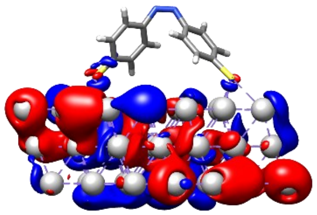<br>State 63<br>Energy: 1.887 eV<br>Osc.: 0.020	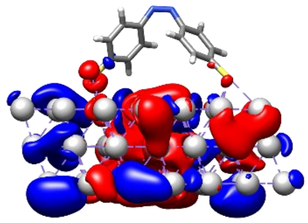<br>State 68<br>Energy: 1.947 eV<br>Osc.: 0.018

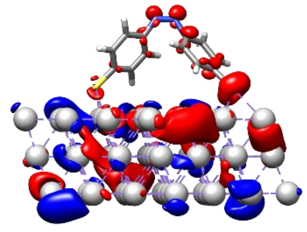

State 70
Energy: 1.975 eV
Osc.: 0.011

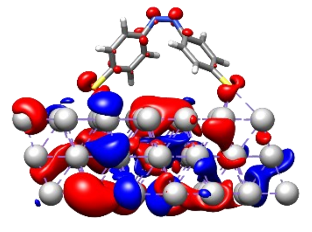

State 73
Energy: 2.012 eV
Osc.: 0.015

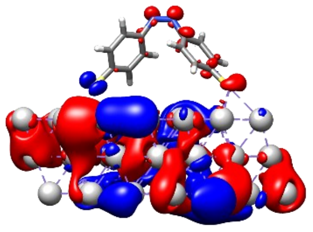

State 74
Energy: 2.026 eV
Osc.: 0.011

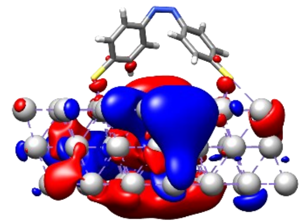

State 76
Energy: 2.053 eV
Osc.: 0.012

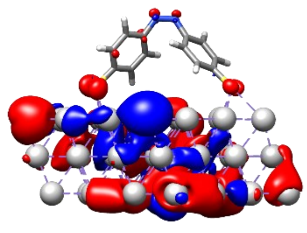

State 79
Energy: 2.074 eV
Osc.: 0.014

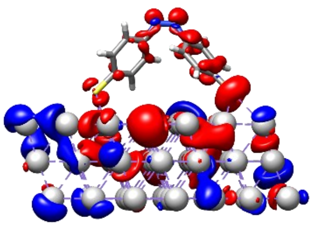

State 81
Energy: 2.110 eV
Osc.: 0.029

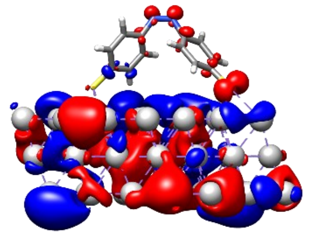

State 82
Energy: 2.126 eV
Osc.: 0.023

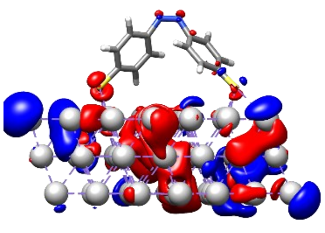

State 84
Energy: 2.159 eV
Osc.: 0.012

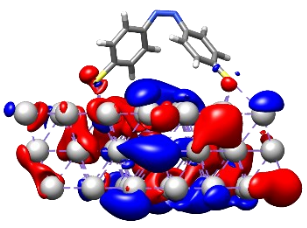

State 85
Energy: 2.171 eV
Osc.: 0.014

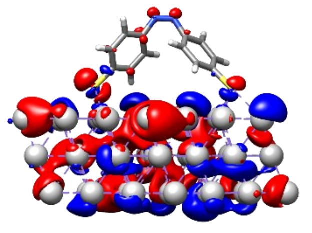

State 86
Energy: 2.193 eV
Osc.: 0.013

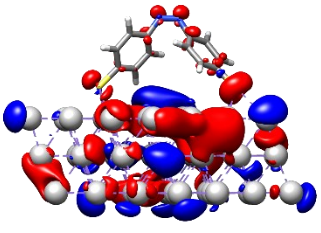

State 87
Energy: 2.200 eV
Osc.: 0.028

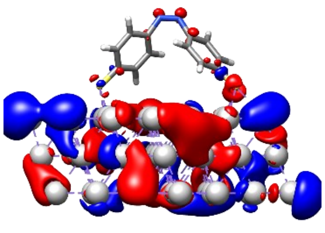

State 89
Energy: 2.216 eV
Osc.: 0.053

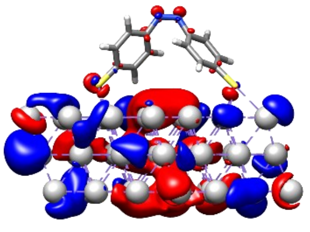

State 91
Energy: 2.234 eV
Osc.: 0.027

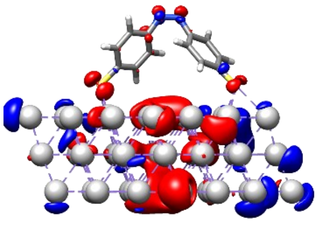

State 93
Energy: 2.281 eV
Osc.: 0.012

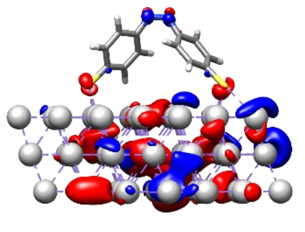

State 94
Energy: 2.298 eV
Osc.: 0.019

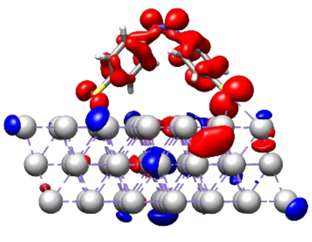

State 96
Energy: 2.311 eV
Osc.: 0.012

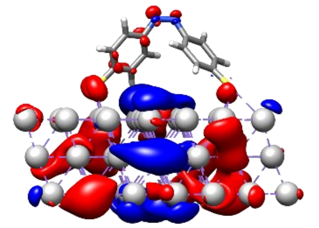

State 98
Energy: 2.326 eV
Osc.: 0.013

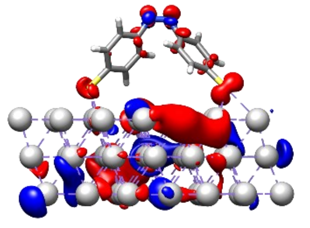

State 99
Energy: 2.332 eV
Osc.: 0.038

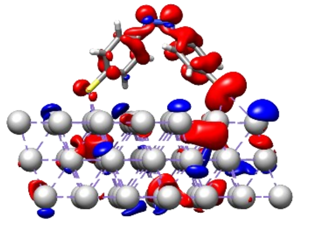

State 101
Energy: 2.355 eV
Osc.: 0.011

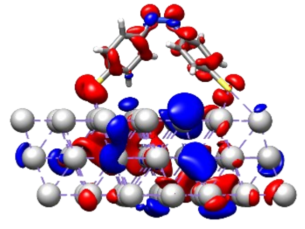

State 102
Energy: 2.361 eV
Osc.: 0.024

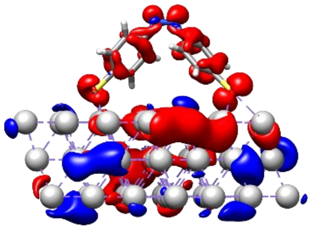

State 106
Energy: 2.405 eV
Osc.: 0.012

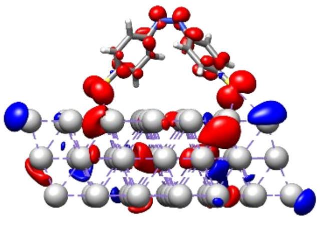

State 108
Energy: 2.421 eV
Osc.: 0.017

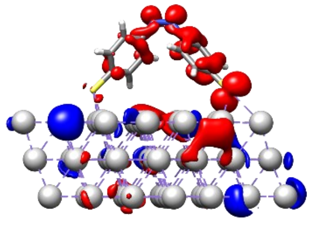

State 111
Energy: 2.454 eV
Osc.: 0.082

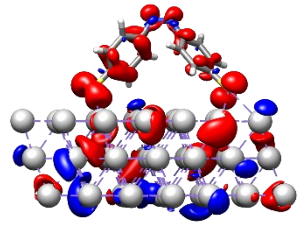

State 114
Energy: 2.476 eV
Osc.: 0.019

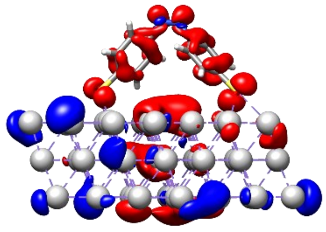

State 116
Energy: 2.493 eV
Osc.: 0.030

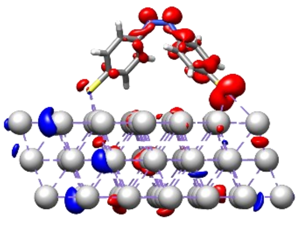

State 117
Energy: 2.496 eV
Osc.: 0.058

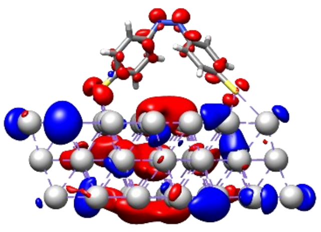

State 118
Energy: 2.501 eV
Osc.: 0.051

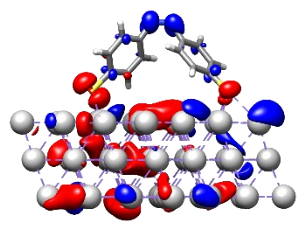

State 121
Energy: 2.539 eV
Osc.: 0.018

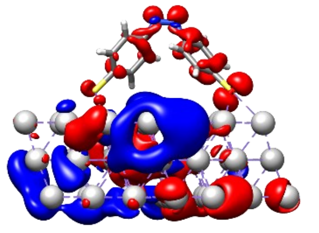

State 123
Energy: 2.569 eV
Osc.: 0.036

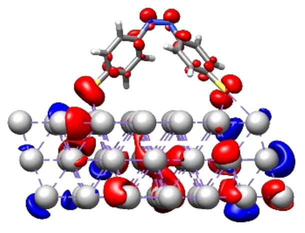

State 126
Energy: 2.592 eV
Osc.: 0.012

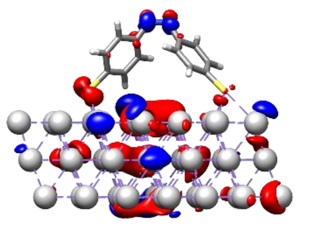

State 127
Energy: 2.597 eV
Osc.: 0.020

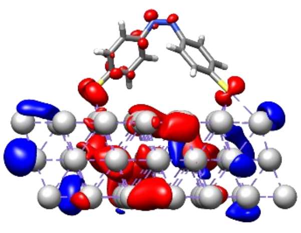

State 129
Energy: 2.621 eV
Osc.: 0.021

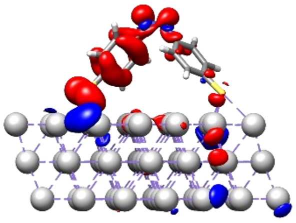

State 130
Energy: 2.631 eV
Osc.: 0.038

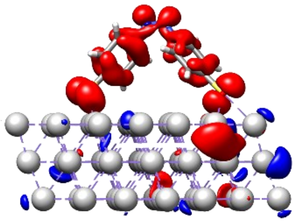

State 131
Energy: 2.636 eV
Osc.: 0.017

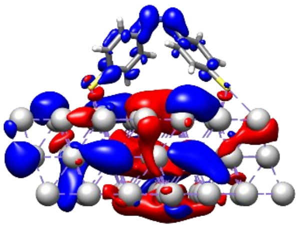

State 133
Energy: 2.649 eV
Osc.: 0.037

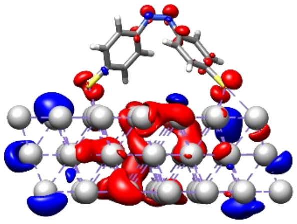

State 134
Energy: 2.662 eV
Osc.: 0.062

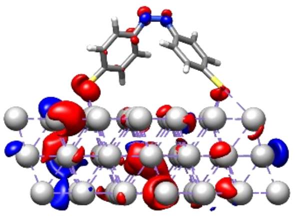

State 135
Energy: 2.663 eV
Osc.: 0.081

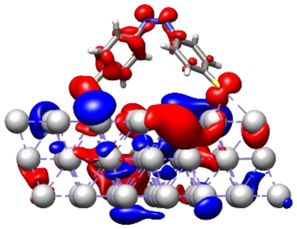

State 136
Energy: 2.676 eV
Osc.: 0.024

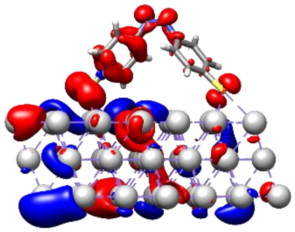

State 137
Energy: 2.688 eV
Osc.: 0.018

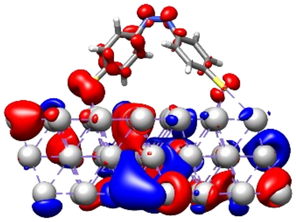

State 138
Energy: 2.694 eV
Osc.: 0.077

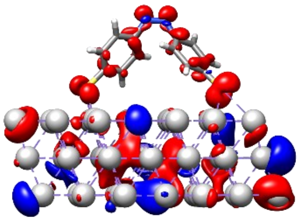

State 139
Energy: 2.701 eV
Osc.: 0.019

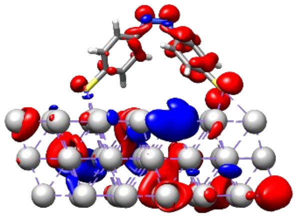

State 141
Energy: 2.726 eV
Osc.: 0.034

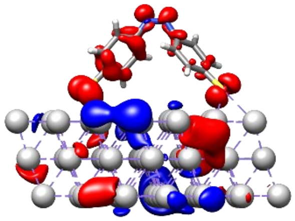

State 142
Energy: 2.744 eV
Osc.: 0.156

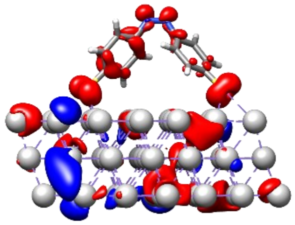

State 144
Energy: 2.756 eV
Osc.: 0.037

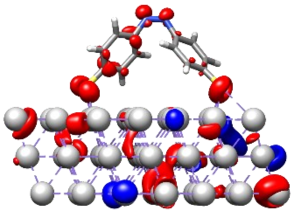

State 145
Energy: 2.760 eV
Osc.: 0.097

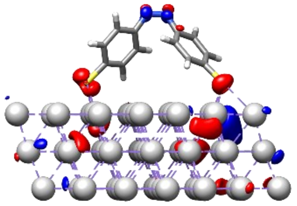

State 146
Energy: 2.776 eV
Osc.: 0.034

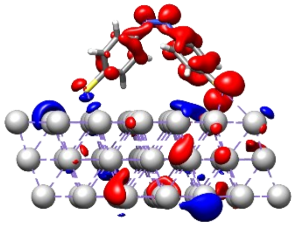

State 147
Energy: 2.785 eV
Osc.: 0.017

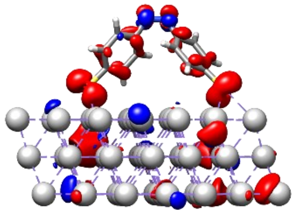

State 148
Energy: 2.789 eV
Osc.: 0.068

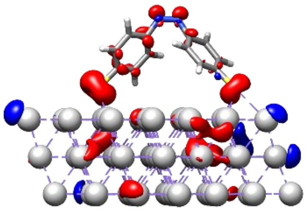

State 149
Energy: 2.796 eV
Osc.: 0.203

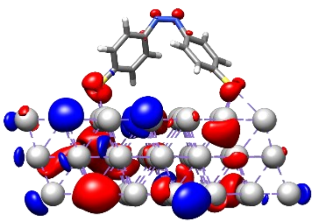

State 150
Energy: 2.800 eV
Osc.: 0.121

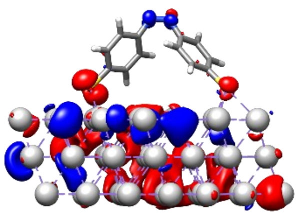

State 151
Energy: 2.803 eV
Osc.: 0.020

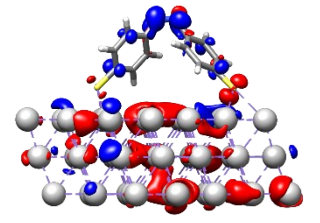

State 152
Energy: 2.817 eV
Osc.: 0.089

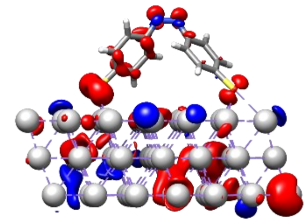

State 153
Energy: 2.835 eV
Osc.: 0.031

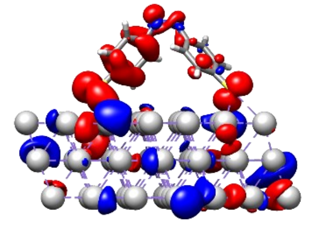

State 154
Energy: 2.839 eV
Osc.: 0.054

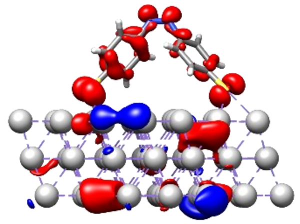

State 155
Energy: 2.852 eV
Osc.: 0.012

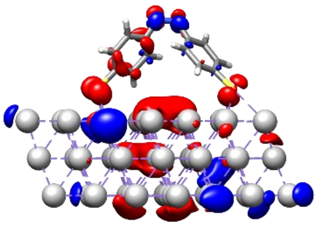

State 157
Energy: 2.868 eV
Osc.: 0.197

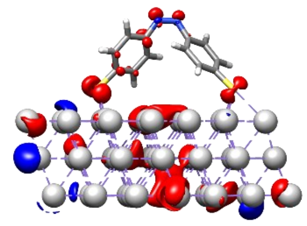

State 158
Energy: 2.876 eV
Osc.: 0.034

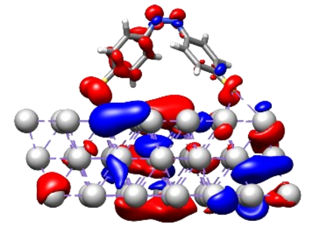

State 159
Energy: 2.888 eV
Osc.: 0.073

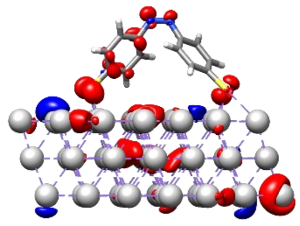

State 160
Energy: 2.896 eV
Osc.: 0.035

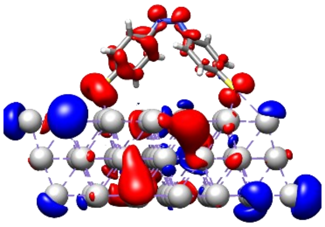

State 161
Energy: 2.899 eV
Osc.: 0.065

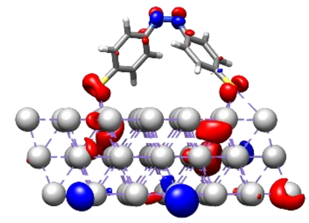

State 162
Energy: 2.910 eV
Osc.: 0.081

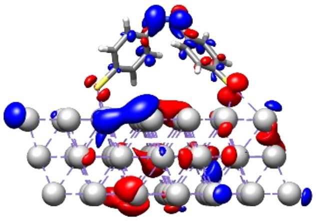

State 165
Energy: 2.933 eV
Osc.: 0.031

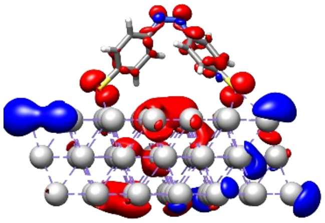

State 166
Energy: 2.936 eV
Osc.: 0.050

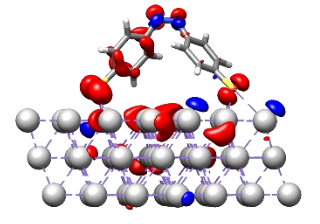

State 167
Energy: 2.945 eV
Osc.: 0.022

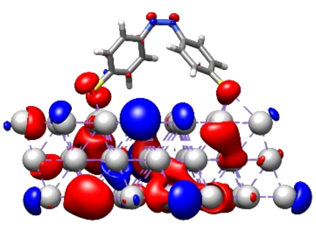

State 168
Energy: 2.952 eV
Osc.: 0.055

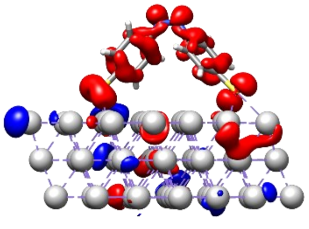

State 169
Energy: 2.960 eV
Osc.: 0.706

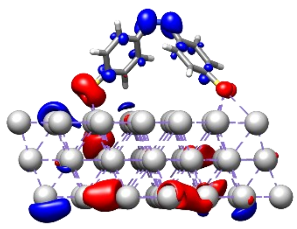

State 170
Energy: 2.973 eV
Osc.: 0.587

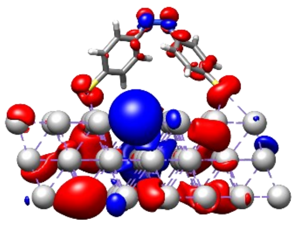

State 171
Energy: 2.978 eV
Osc.: 0.135

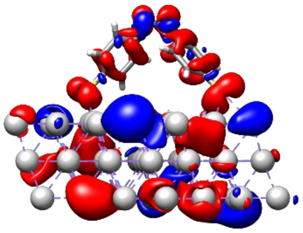

State 172
Energy: 2.982 eV
Osc.: 0.227

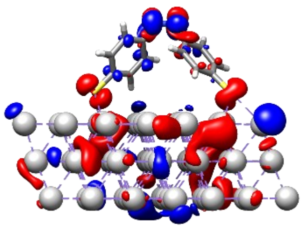

State 173
Energy: 2.990 eV
Osc.: 0.031

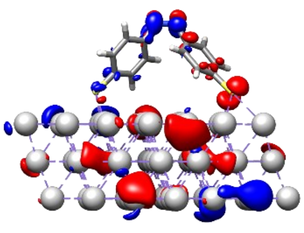

State 174
Energy: 2.999 eV
Osc.: 0.014

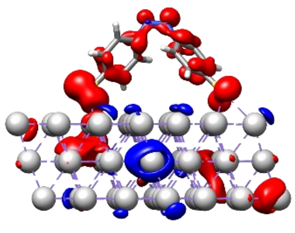

State 175
Energy: 3.012 eV
Osc.: 0.159

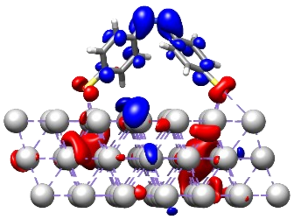

State 176
Energy: 3.016 eV
Osc.: 0.348

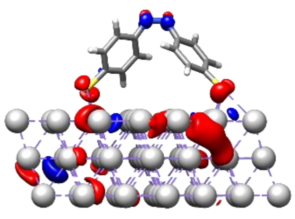

State 177
Energy: 3.026 eV
Osc.: 0.117

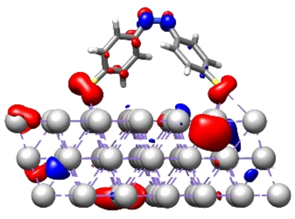

State 178
Energy: 3.030 eV
Osc.: 0.293

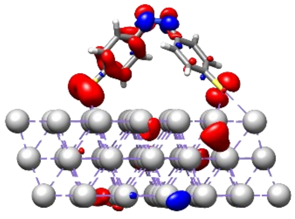

State 179
Energy: 3.039 eV
Osc.: 0.015

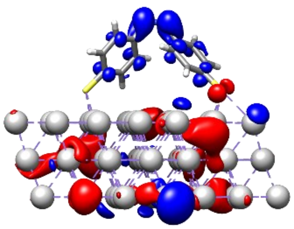

State 180
Energy: 3.049 eV
Osc.: 0.035

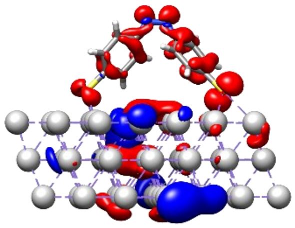

State 181
Energy: 3.057 eV
Osc.: 0.046

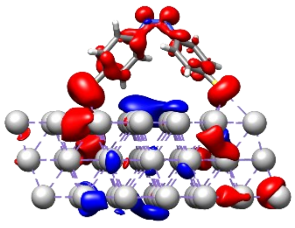

State 182
Energy: 3.058 eV
Osc.: 0.140

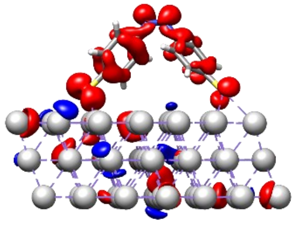

State 183
Energy: 3.062 eV
Osc.: 0.069

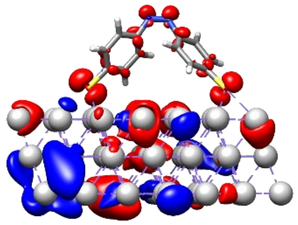<br>State 184<br>Energy: 3.071 eV<br>Osc.: 0.195	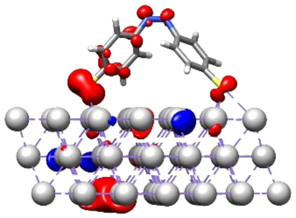<br>State 185<br>Energy: 3.079 eV<br>Osc.: 0.026	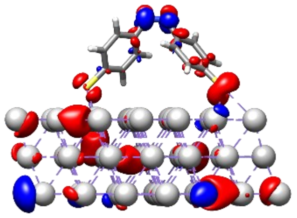<br>State 186<br>Energy: 3.083 eV<br>Osc.: 0.074	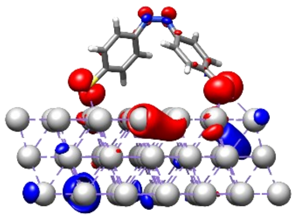<br>State 188<br>Energy: 3.106 eV<br>Osc.: 0.294
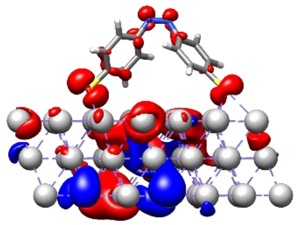<br>State 189<br>Energy: 3.120 eV<br>Osc.: 0.021	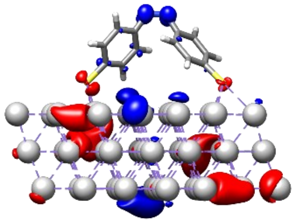<br>State 190<br>Energy: 3.124 eV<br>Osc.: 0.031	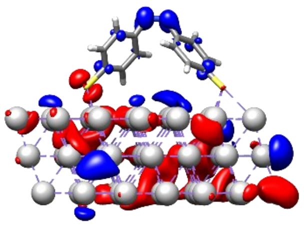<br>State 191<br>Energy: 3.132 eV<br>Osc.: 0.027	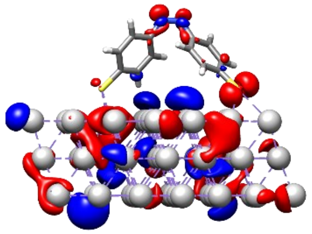<br>State 192<br>Energy: 3.141 eV<br>Osc.: 0.010
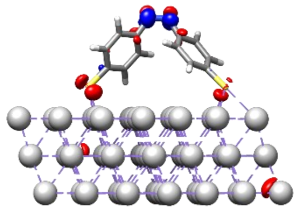<br>State 193<br>Energy: 3.144 eV<br>Osc.: 0.034	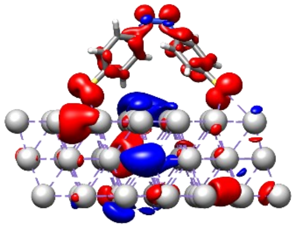<br>State 194<br>Energy: 3.149 eV<br>Osc.: 0.018	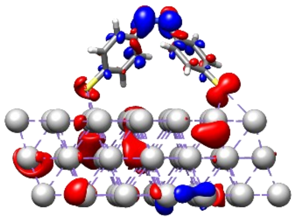<br>State 196<br>Energy: 3.167 eV<br>Osc.: 0.059	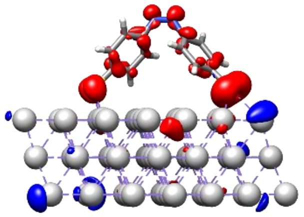<br>State 197<br>Energy: 3.177 eV<br>Osc.: 0.041
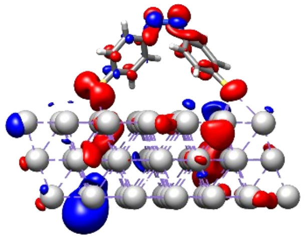<br>State 198<br>Energy: 3.187 eV<br>Osc.: 0.022	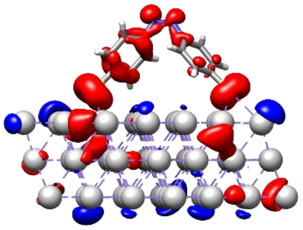<br>State 199<br>Energy: 3.190 eV<br>Osc.: 0.035	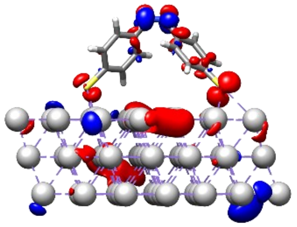<br>State 200<br>Energy: 3.192 eV<br>Osc.: 0.091	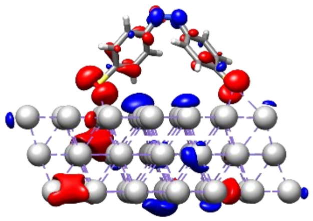<br>State 201<br>Energy: 3.201 eV<br>Osc.: 0.073

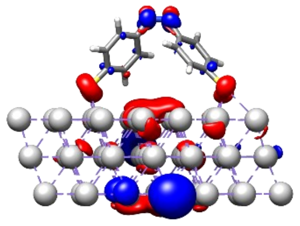<br>State 202<br>Energy: 3.216 eV<br>Osc.: 0.114	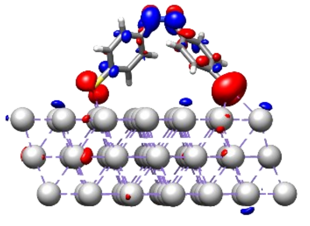<br>State 203<br>Energy: 3.222 eV<br>Osc.: 0.101	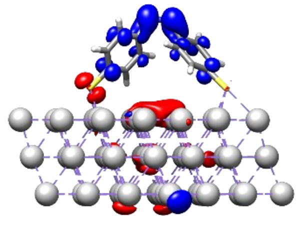<br>State 205<br>Energy: 3.230 eV<br>Osc.: 0.057	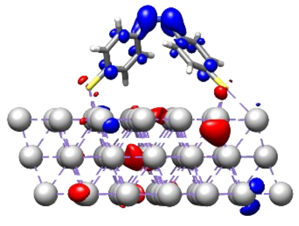<br>State 206<br>Energy: 3.236 eV<br>Osc.: 0.077
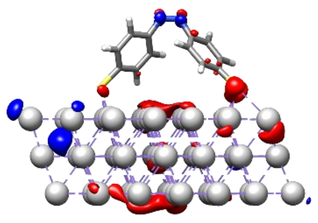<br>State 207<br>Energy: 3.243 eV<br>Osc.: 0.221	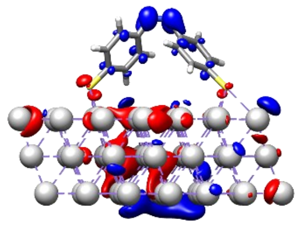<br>State 209<br>Energy: 3.256 eV<br>Osc.: 0.065	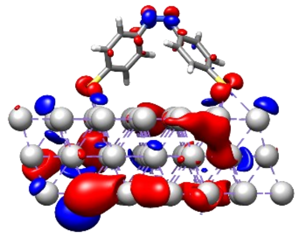<br>State 210<br>Energy: 3.264 eV<br>Osc.: 0.109	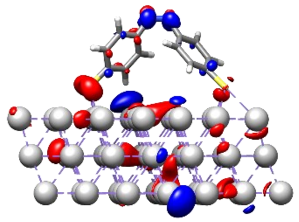<br>State 211<br>Energy: 3.271 eV<br>Osc.: 0.035
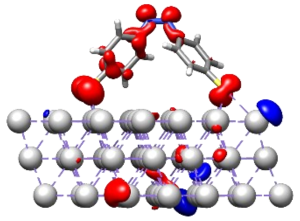<br>State 212<br>Energy: 3.278 eV<br>Osc.: 0.027	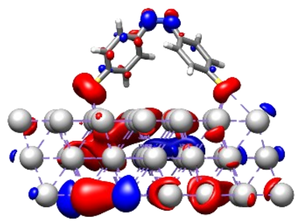<br>State 213<br>Energy: 3.282 eV<br>Osc.: 0.035	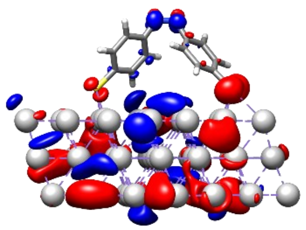<br>State 214<br>Energy: 3.287 eV<br>Osc.: 0.016	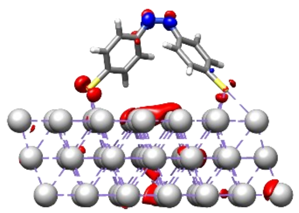<br>State 215<br>Energy: 3.292 eV<br>Osc.: 0.053
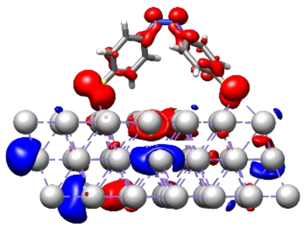<br>State 216<br>Energy: 3.297 eV<br>Osc.: 0.022	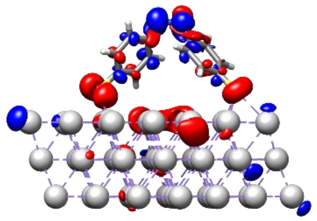<br>State 218<br>Energy: 3.311 eV<br>Osc.: 0.037	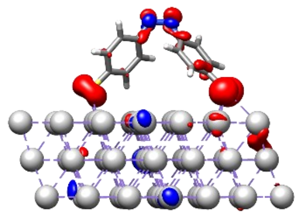<br>State 220<br>Energy: 3.324 eV<br>Osc.: 0.061	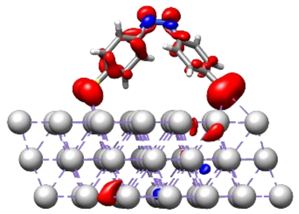<br>State 221<br>Energy: 3.335 eV<br>Osc.: 0.019

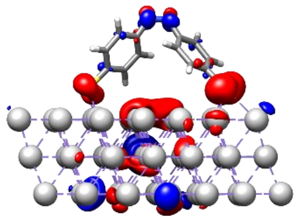<br>State 222<br>Energy: 3.340 eV<br>Osc.: 0.176	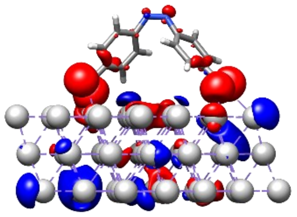<br>State 223<br>Energy: 3.350 eV<br>Osc.: 0.147	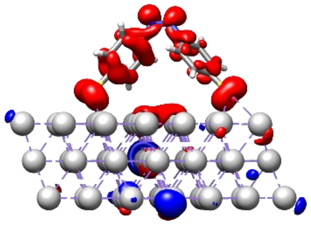<br>State 224<br>Energy: 3.358 eV<br>Osc.: 0.022	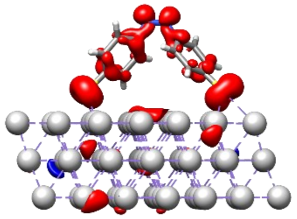<br>State 225<br>Energy: 3.367 eV<br>Osc.: 0.060
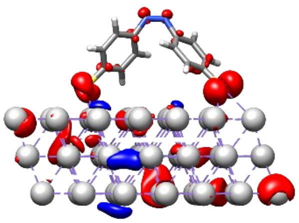<br>State 227<br>Energy: 3.380 eV<br>Osc.: 0.044	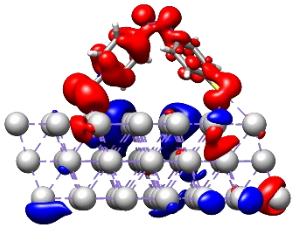<br>State 228<br>Energy: 3.386 eV<br>Osc.: 0.102	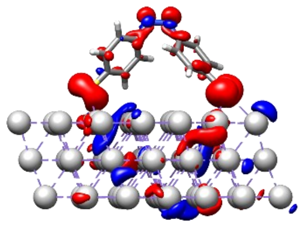<br>State 229<br>Energy: 3.391 eV<br>Osc.: 0.020	

Table S5: Charge density differences (CDDs) illustrating the nature of the low-lying bright excitations of intermediate A. Charge transfer takes place from red to blue

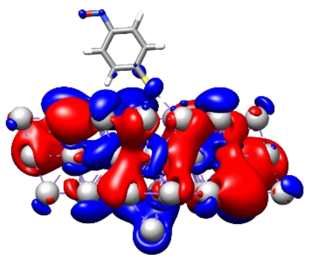<br>State 45<br>Energy: 1.492 eV<br>Osc.: 0.015	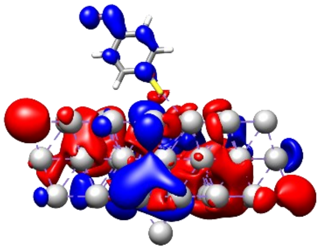<br>State 52<br>Energy: 1.586 eV<br>Osc.: 0.011	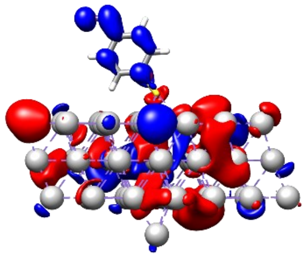<br>State 53<br>Energy: 1.626 eV<br>Osc.: 0.010	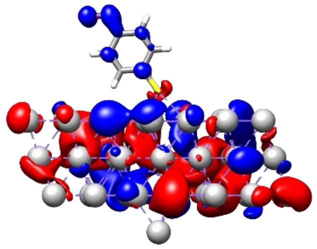<br>State 54<br>Energy: 1.637 eV<br>Osc.: 0.014

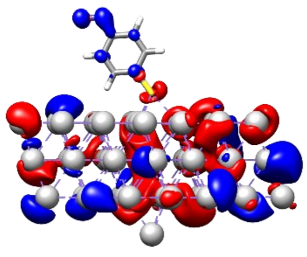

State 56
Energy: 1.688 eV
Osc.: 0.010

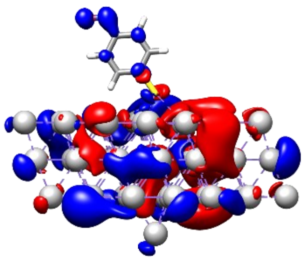

State 66
Energy: 1.846 eV
Osc.: 0.023

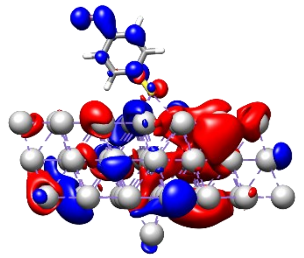

State 67
Energy: 1.863 eV
Osc.: 0.010

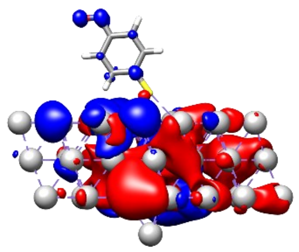

State 74
Energy: 1.934 eV
Osc.: 0.012

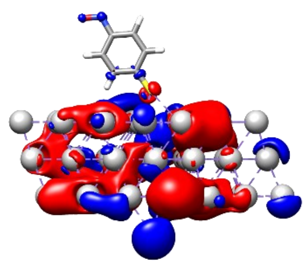

State 85
Energy: 2.083 eV
Osc.: 0.014

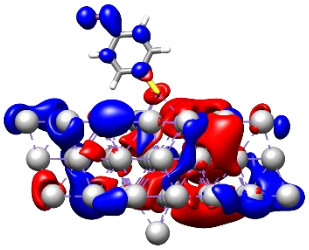

State 88
Energy: 2.131 eV
Osc.: 0.017

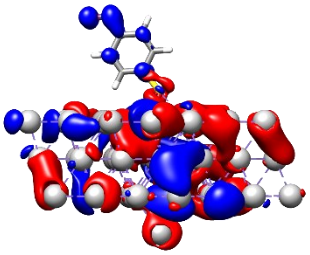

State 90
Energy: 2.159 eV
Osc.: 0.010

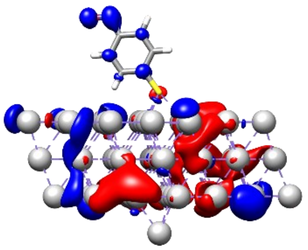

State 91
Energy: 2.170 eV
Osc.: 0.015

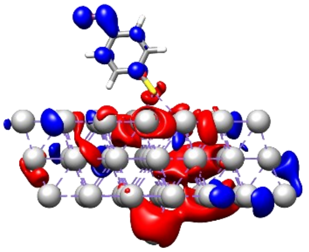

State 100
Energy: 2.276 eV
Osc.: 0.012

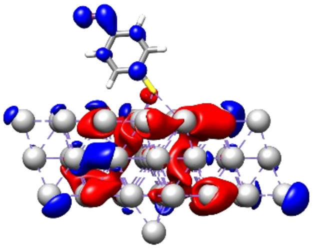

State 103
Energy: 2.300 eV
Osc.: 0.014

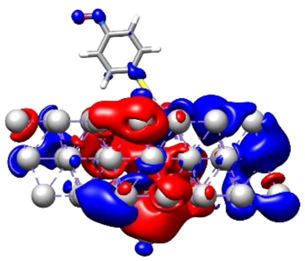

State 104
Energy: 2.311 eV
Osc.: 0.010

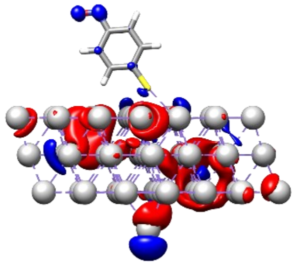

State 107
Energy: 2.354 eV
Osc.: 0.018

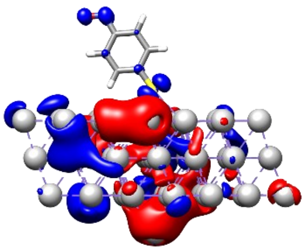

State 109
Energy: 2.369 eV
Osc.: 0.016

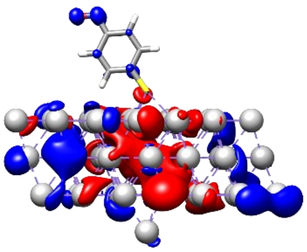

State 110
Energy: 2.382 eV
Osc.: 0.039

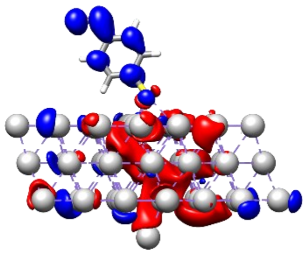

State 111
Energy: 2.394 eV
Osc.: 0.023

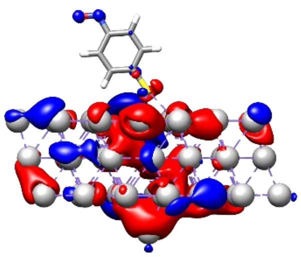

State 112
Energy: 2.403 eV
Osc.: 0.045

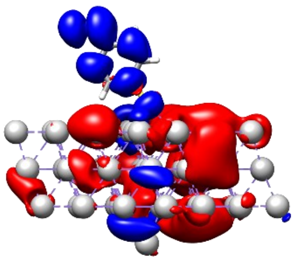

State 113
Energy: 2.413 eV
Osc.: 0.051

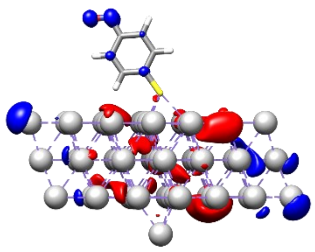

State 114
Energy: 2.430 eV
Osc.: 0.067

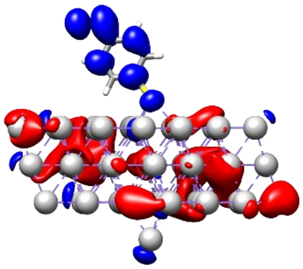

State 115
Energy: 2.442 eV
Osc.: 0.017

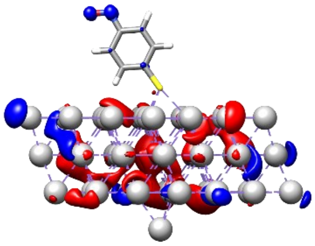

State 116
Energy: 2.448 eV
Osc.: 0.020

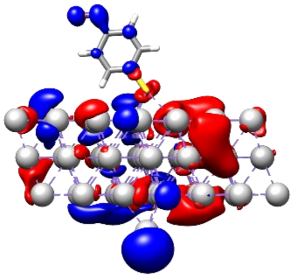

State 117
Energy: 2.457 eV
Osc.: 0.011

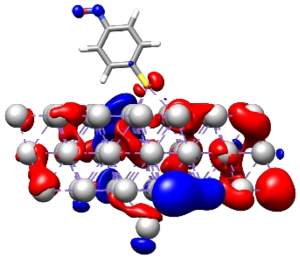

State 121
Energy: 2.489 eV
Osc.: 0.022

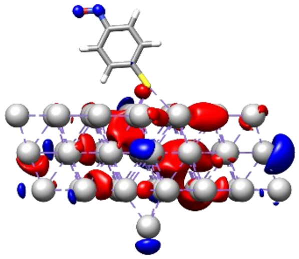

State 122
Energy: 2.500 eV
Osc.: 0.017

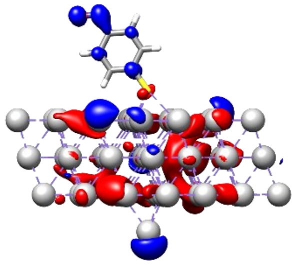

State 126
Energy: 2.538 eV
Osc.: 0.018

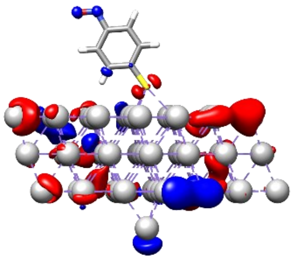

State 127
Energy: 2.542 eV
Osc.: 0.010

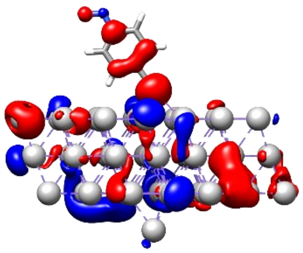

State 128
Energy: 2.558 eV
Osc.: 0.012

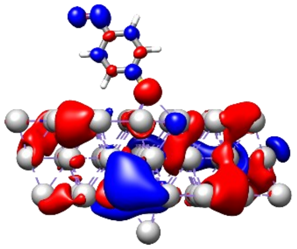

State 129
Energy: 2.565 eV
Osc.: 0.012

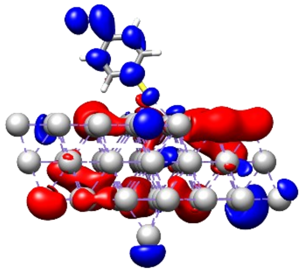

State 131
Energy: 2.585 eV
Osc.: 0.034

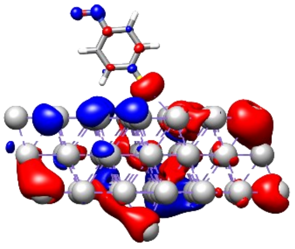

State 132
Energy: 2.590 eV
Osc.: 0.042

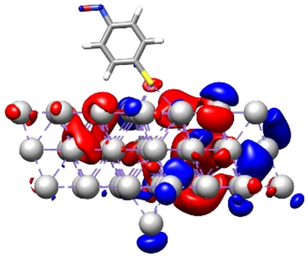

State 134
Energy: 2.608 eV
Osc.: 0.029

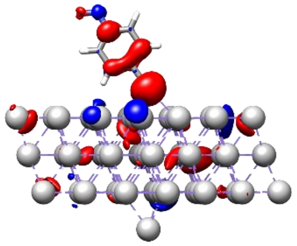

State 135
Energy: 2.619 eV
Osc.: 0.058

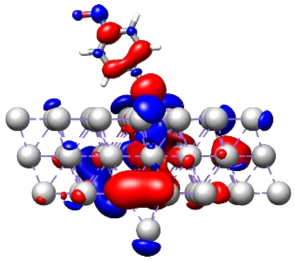

State 138
Energy: 2.639 eV
Osc.: 0.079

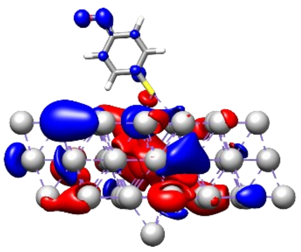

State 139
Energy: 2.653 eV
Osc.: 0.013

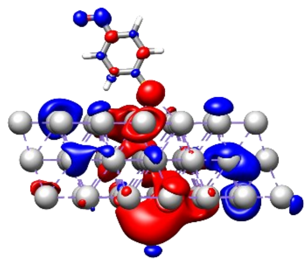

State 141
Energy: 2.658 eV
Osc.: 0.017

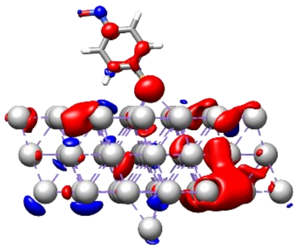

State 142
Energy: 2.664 eV
Osc.: 0.066

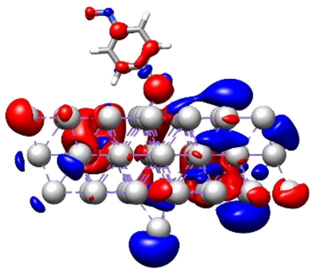

State 144
Energy: 2.680 eV
Osc.: 0.055

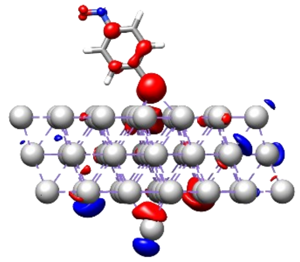

State 147
Energy: 2.701 eV
Osc.: 0.010

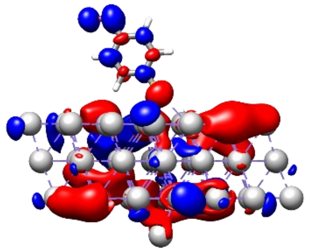

State 148
Energy: 2.724 eV
Osc.: 0.053

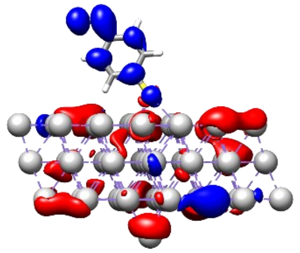

State 149
Energy: 2.734 eV
Osc.: 0.015

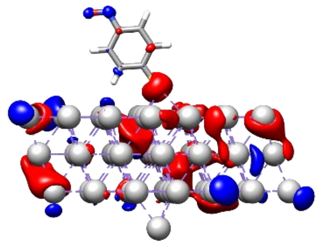

State 150
Energy: 2.738 eV
Osc.: 0.055

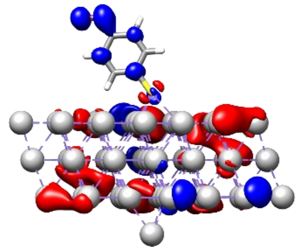

State 151
Energy: 2.748 eV
Osc.: 0.014

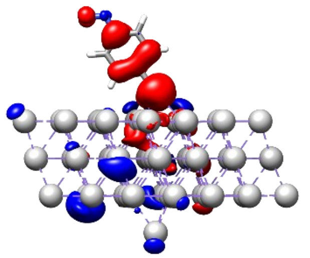

State 153
Energy: 2.767 eV
Osc.: 0.016

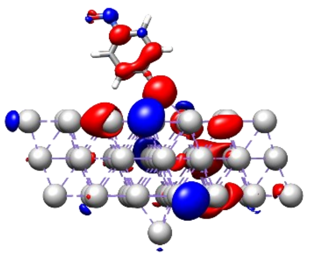

State 155
Energy: 2.784 eV
Osc.: 0.027

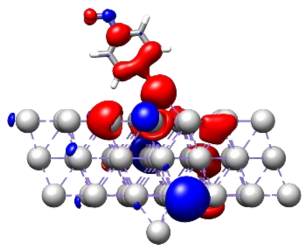

State 156
Energy: 2.787 eV
Osc.: 0.039

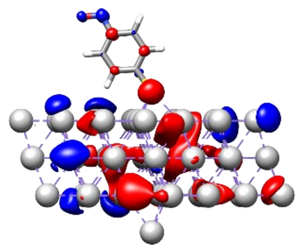

State 157
Energy: 2.795 eV
Osc.: 0.011

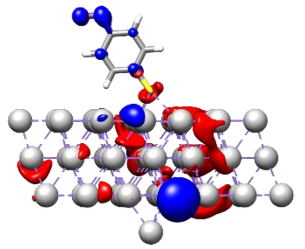

State 158
Energy: 2.807 eV
Osc.: 0.041

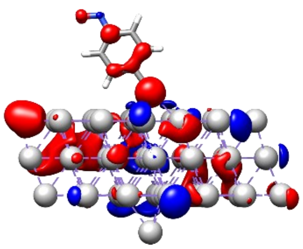

State 160
Energy: 2.823 eV
Osc.: 0.084

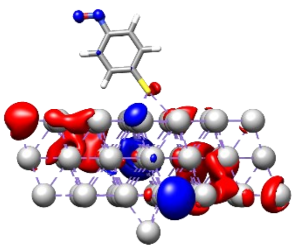

State 161
Energy: 2.834 eV
Osc.: 0.039

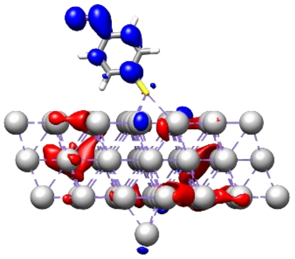<br>State 162<br>Energy: 2.840 eV<br>Osc.: 0.031	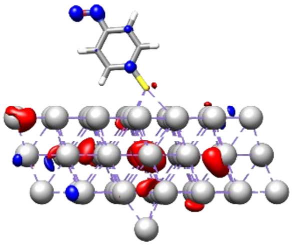<br>State 164<br>Energy: 2.853 eV<br>Osc.: 0.057	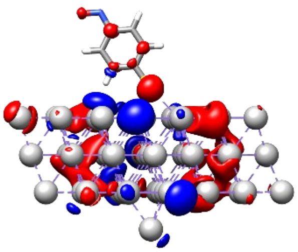<br>State 165<br>Energy: 2.868 eV<br>Osc.: 0.137	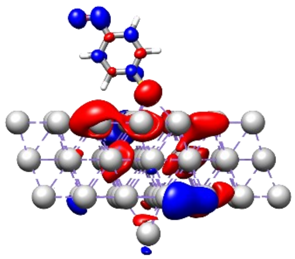<br>State 166<br>Energy: 2.879 eV<br>Osc.: 0.034
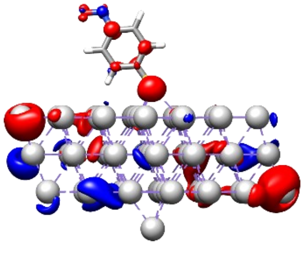<br>State 167<br>Energy: 2.890 eV<br>Osc.: 0.176	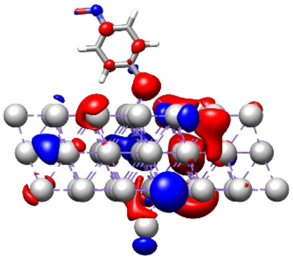<br>State 168<br>Energy: 2.891 eV<br>Osc.: 0.034	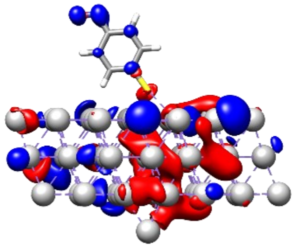<br>State 169<br>Energy: 2.900 eV<br>Osc.: 0.040	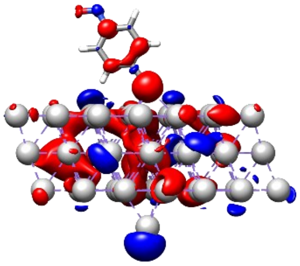<br>State 170<br>Energy: 2.907 eV<br>Osc.: 0.016
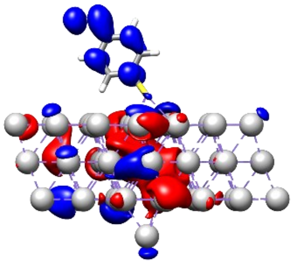<br>State 171<br>Energy: 2.912 eV<br>Osc.: 0.015	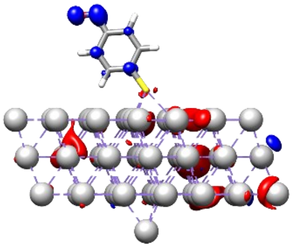<br>State 172<br>Energy: 2.920 eV<br>Osc.: 0.070	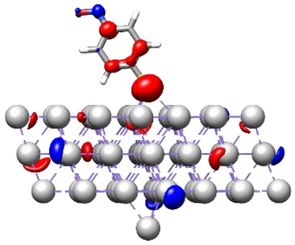<br>State 173<br>Energy: 2.933 eV<br>Osc.: 0.042	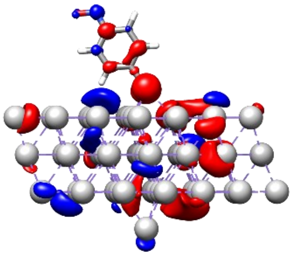<br>State 174<br>Energy: 2.937 eV<br>Osc.: 0.112
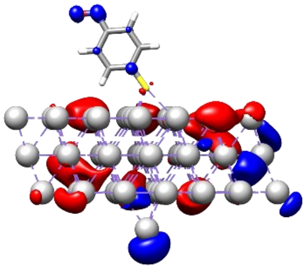<br>State 175<br>Energy: 2.946 eV<br>Osc.: 0.266	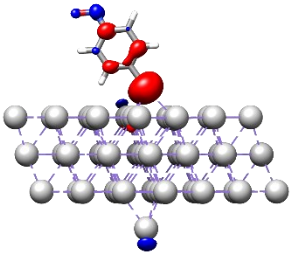<br>State 178<br>Energy: 2.964 eV<br>Osc.: 0.542	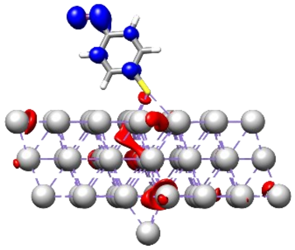<br>State 179<br>Energy: 2.968 eV<br>Osc.: 0.236	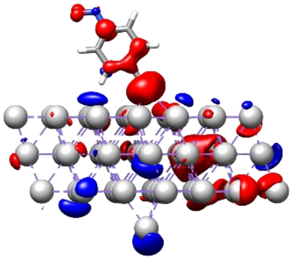<br>State 180<br>Energy: 2.986 eV<br>Osc.: 0.021

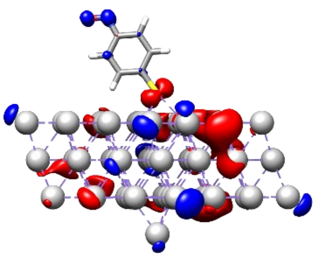<br>State 181<br>Energy: 2.995 eV<br>Osc.: 0.011	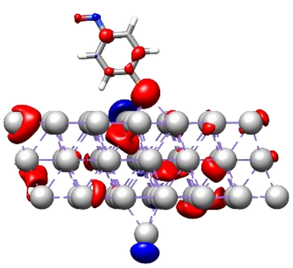<br>State 182<br>Energy: 2.996 eV<br>Osc.: 0.022	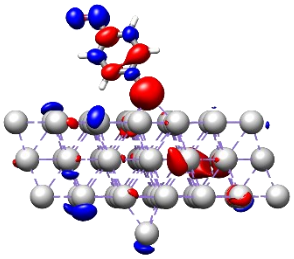<br>State 183<br>Energy: 3.007 eV<br>Osc.: 0.011	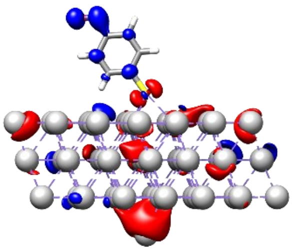<br>State 185<br>Energy: 3.018 eV<br>Osc.: 0.010
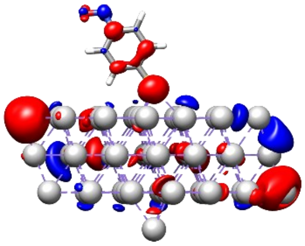<br>State 186<br>Energy: 3.028 eV<br>Osc.: 0.021	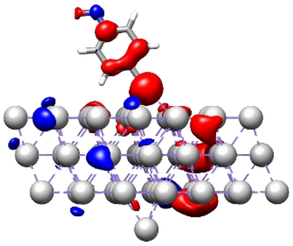<br>State 187<br>Energy: 3.035 eV<br>Osc.: 0.010	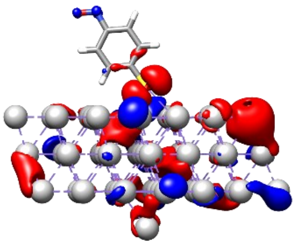<br>State 188<br>Energy: 3.040 eV<br>Osc.: 0.037	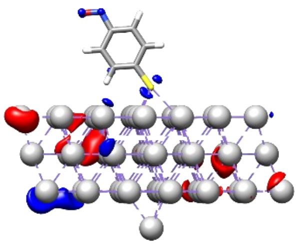<br>State 189<br>Energy: 3.046 eV<br>Osc.: 0.908
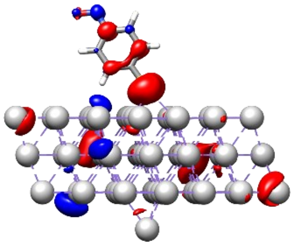<br>State 191<br>Energy: 3.063 eV<br>Osc.: 0.206	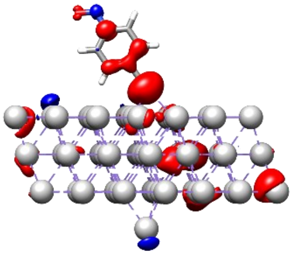<br>State 193<br>Energy: 3.080 eV<br>Osc.: 0.326	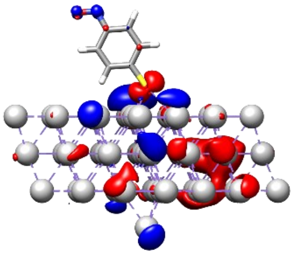<br>State 194<br>Energy: 3.088 eV<br>Osc.: 0.204	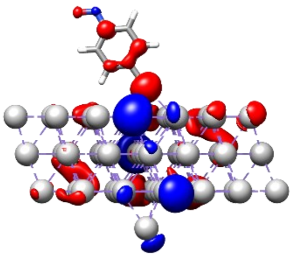<br>State 196<br>Energy: 3.103 eV<br>Osc.: 0.186
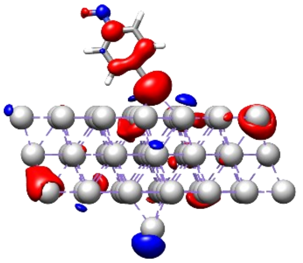<br>State 197<br>Energy: 3.108 eV<br>Osc.: 0.011	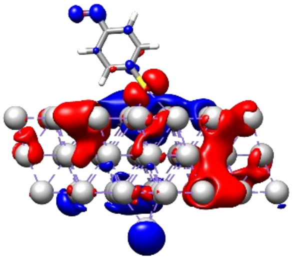<br>State 198<br>Energy: 3.116 eV<br>Osc.: 0.010	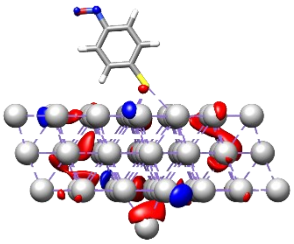<br>State 199<br>Energy: 3.119 eV<br>Osc.: 0.656	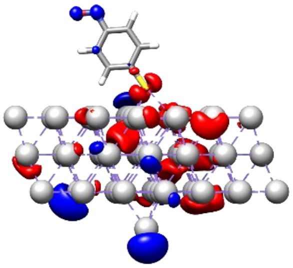<br>State 200<br>Energy: 3.123 eV<br>Osc.: 0.044

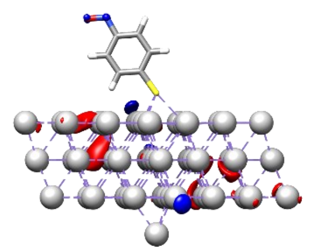

State 201
Energy: 3.133 eV
Osc.: 0.083

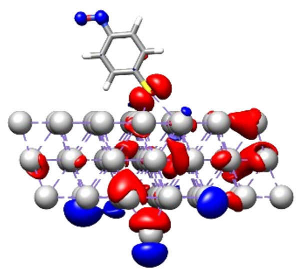

State 202
Energy: 3.139 eV
Osc.: 0.065

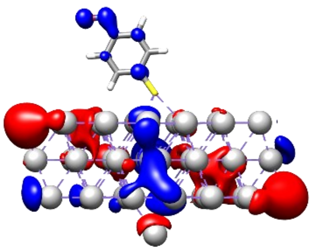

State 205
Energy: 3.161 eV
Osc.: 0.112

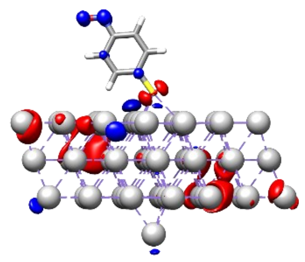

State 206
Energy: 3.170 eV
Osc.: 0.028

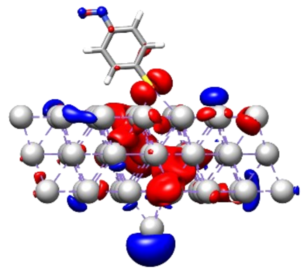

State 207
Energy: 3.175 eV
Osc.: 0.144

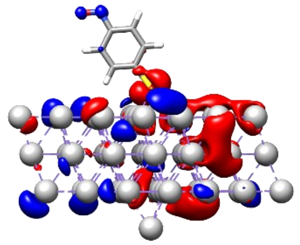

State 208
Energy: 3.181 eV
Osc.: 1.155

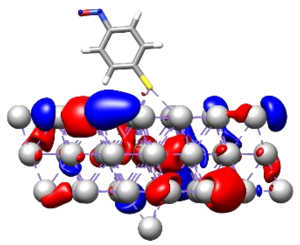

State 209
Energy: 3.188 eV
Osc.: 0.046

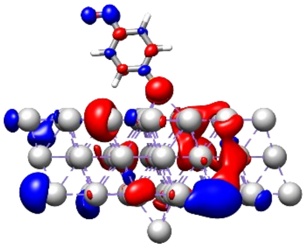

State 210
Energy: 3.193 eV
Osc.: 0.017

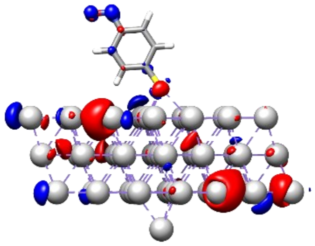

State 211
Energy: 3.205 eV
Osc.: 0.052

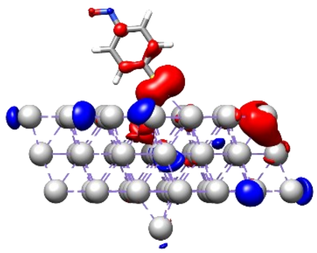

State 212
Energy: 3.208 eV
Osc.: 0.214

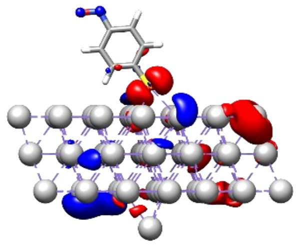

State 213
Energy: 3.213 eV
Osc.: 0.034

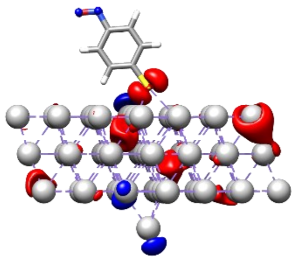

State 214
Energy: 3.220 eV
Osc.: 0.101

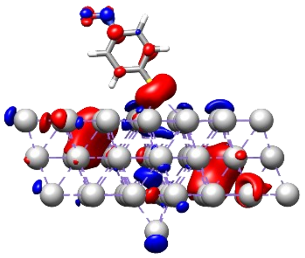

State 215
Energy: 3.224 eV
Osc.: 0.047

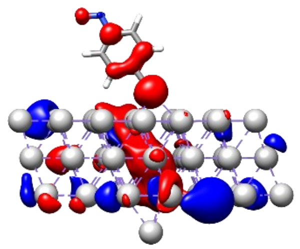

State 216
Energy: 3.237 eV
Osc.: 0.080

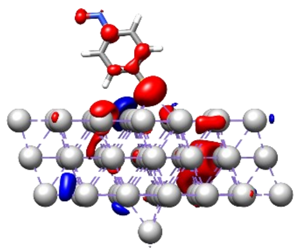

State 217
Energy: 3.243 eV
Osc.: 0.250

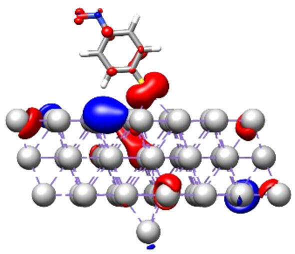

State 218
Energy: 3.247 eV
Osc.: 0.209

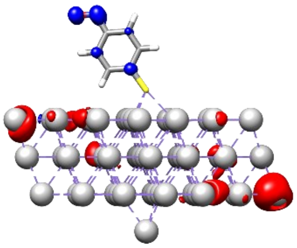

State 219
Energy: 3.259 eV
Osc.: 0.086

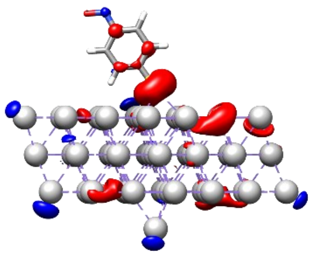

State 220
Energy: 3.270 eV
Osc.: 0.027

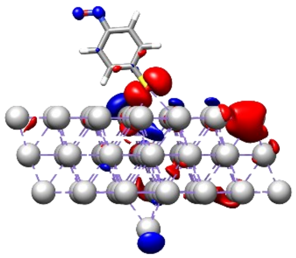

State 221
Energy: 3.286 eV
Osc.: 0.021

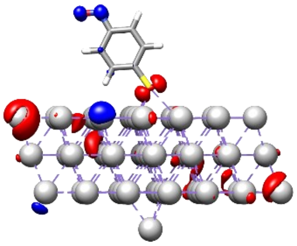

State 222
Energy: 3.289 eV
Osc.: 0.047

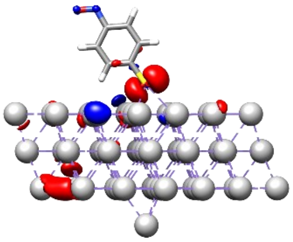

State 223
Energy: 3.299 eV
Osc.: 0.098

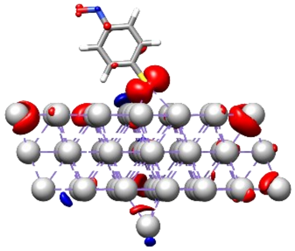

State 225
Energy: 3.306 eV
Osc.: 0.078

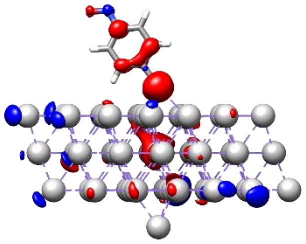

State 226
Energy: 3.310 eV
Osc.: 0.085

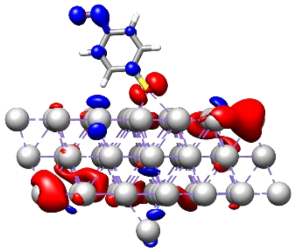

State 227
Energy: 3.315 eV
Osc.: 0.047

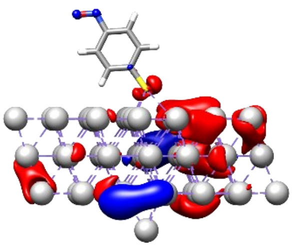

State 228
Energy: 3.322 eV
Osc.: 0.141

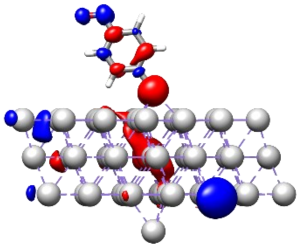

State 229
Energy: 3.328 eV
Osc.: 0.002

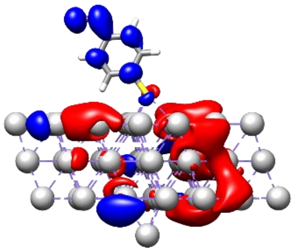

State 230
Energy: 3.334 eV
Osc.: 0.077

Table S6: Charge density differences (CDDs) illustrating the nature of the low-lying bright excitations of intermediate B. Charge transfer takes place from red to blue

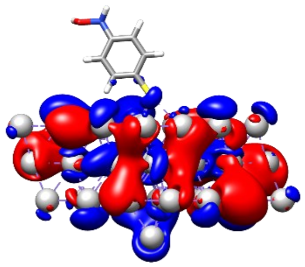<br>State 44<br>Energy: 1.494 eV<br>Osc.: 0.016	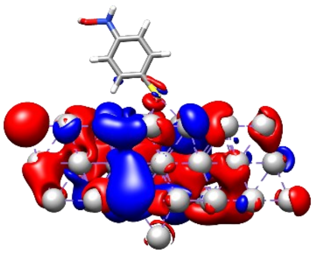<br>State 49<br>Energy: 1.569 eV<br>Osc.: 0.013	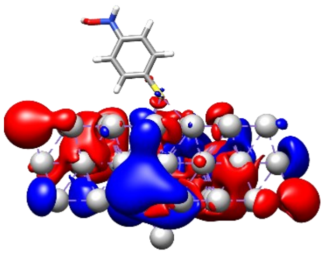<br>State 51<br>Energy: 1.588 eV<br>Osc.: 0.010	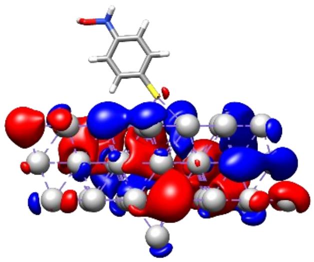<br>State 53<br>Energy: 1.642 eV<br>Osc.: 0.017
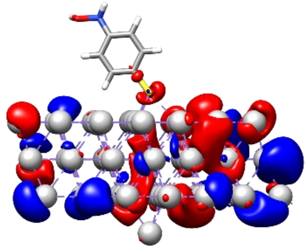<br>State 55<br>Energy: 1.693 eV<br>Osc.: 0.010	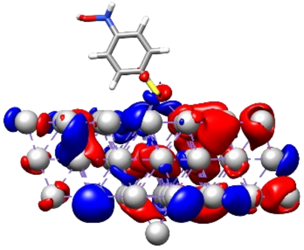<br>State 65<br>Energy: 1.844 eV<br>Osc.: 0.033	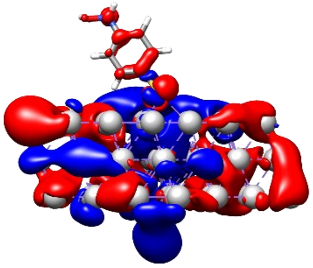<br>State 83<br>Energy: 2.063 eV<br>Osc.: 0.013	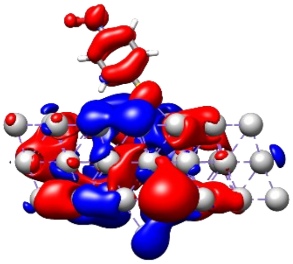<br>State 84<br>Energy: 2.071 eV<br>Osc.: 0.010
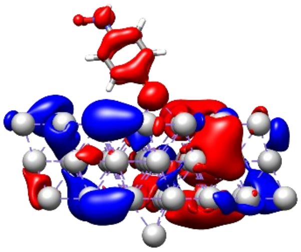<br>State 87<br>Energy: 2.120 eV<br>Osc.: 0.012	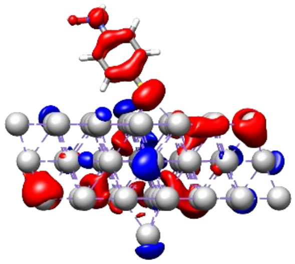<br>State 88<br>Energy: 2.141 eV<br>Osc.: 0.011	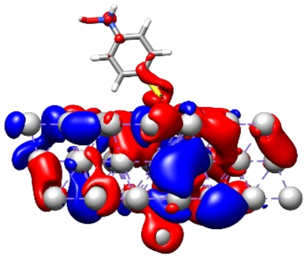<br>State 89<br>Energy: 2.153 eV<br>Osc.: 0.014	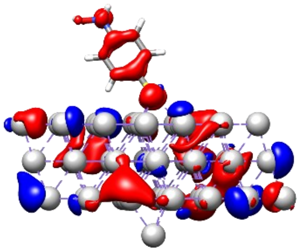<br>State 90<br>Energy: 2.172 eV<br>Osc.: 0.015

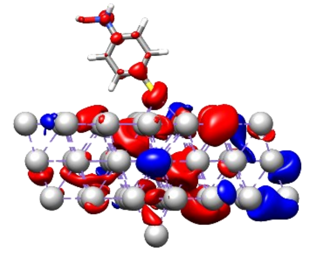

State 100
Energy: 2.276 eV
Osc.: 0.014

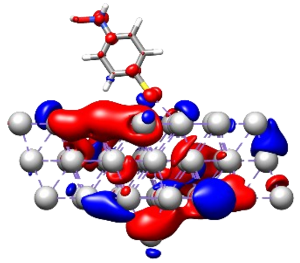

State 101
Energy: 2.288 eV
Osc.: 0.021

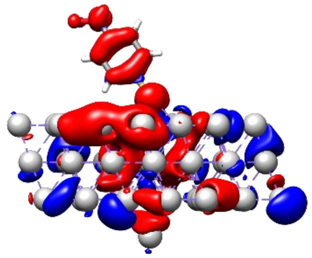

State 104
Energy: 2.312 eV
Osc.: 0.017

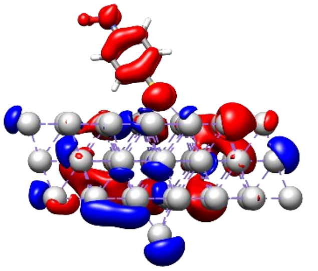

State 106
Energy: 2.335 eV
Osc.: 0.010

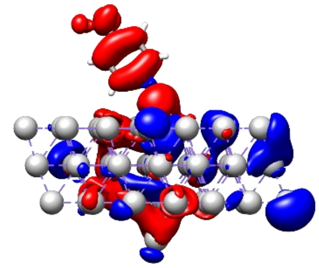

State 107
Energy: 2.342 eV
Osc.: 0.011

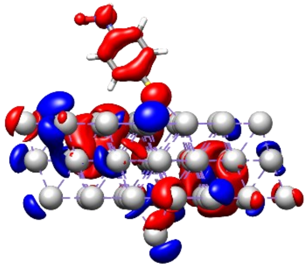

State 108
Energy: 2.357 eV
Osc.: 0.010

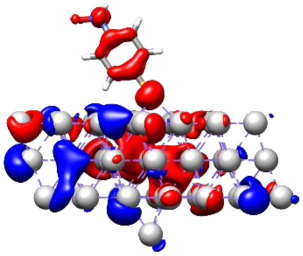

State 111
Energy: 2.378 eV
Osc.: 0.020

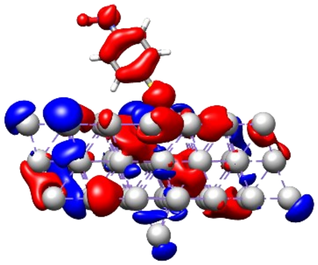

State 112
Energy: 2.387 eV
Osc.: 0.045

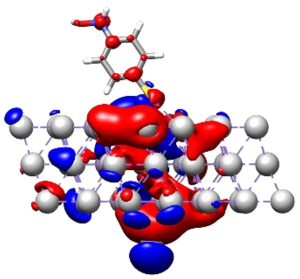

State 113
Energy: 2.401 eV
Osc.: 0.038

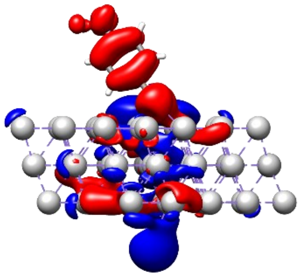

State 114
Energy: 2.427 eV
Osc.: 0.018

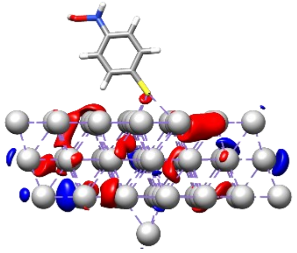

State 115
Energy: 2.436 eV
Osc.: 0.085

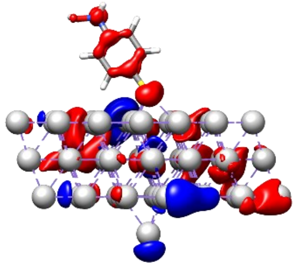

State 118
Energy: 2.457 eV
Osc.: 0.022

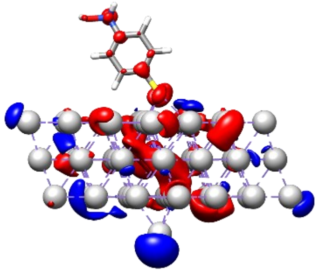

State 119
Energy: 2.461 eV
Osc.: 0.020

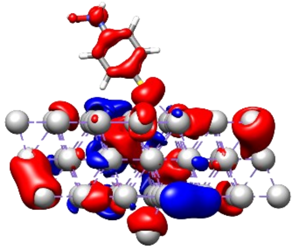

State 121
Energy: 2.484 eV
Osc.: 0.028

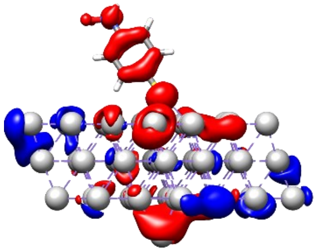

State 123
Energy: 2.495 eV
Osc.: 0.016

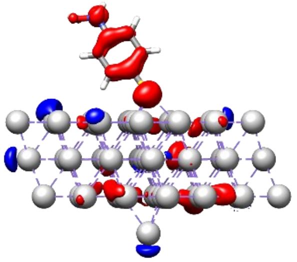

State 126
Energy: 2.534 eV
Osc.: 0.024

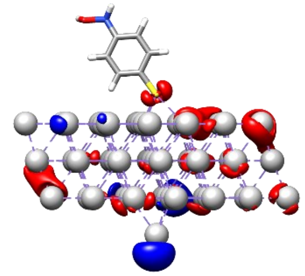

State 131
Energy: 2.584 eV
Osc.: 0.124

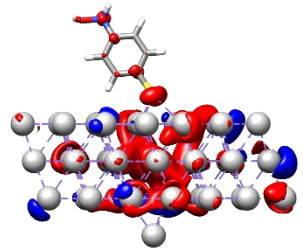

State 132
Energy: 2.589 eV
Osc.: 0.017

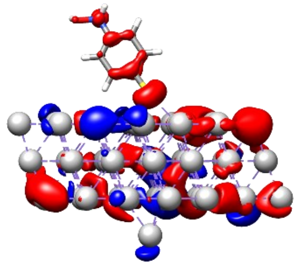

State 133
Energy: 2.601 eV
Osc.: 0.028

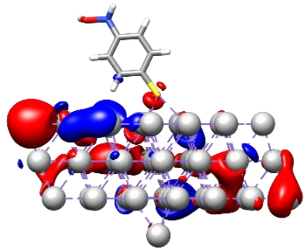

State 136
Energy: 2.627 eV
Osc.: 0.075

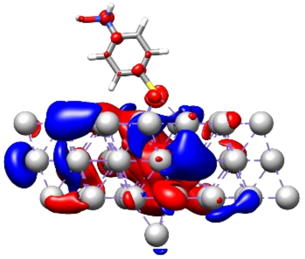

State 140
Energy: 2.651 eV
Osc.: 0.026

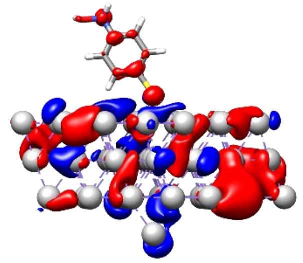

State 141
Energy: 2.662 eV
Osc.: 0.126

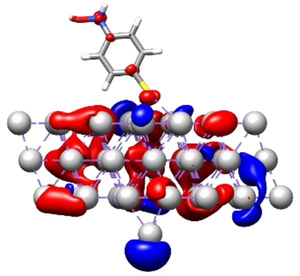

State 142
Energy: 2.664 eV
Osc.: 0.036

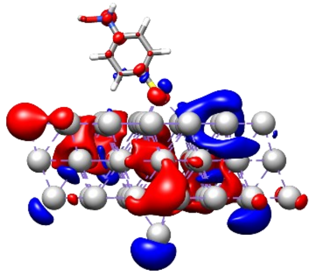

State 143
Energy: 2.679 eV
Osc.: 0.014

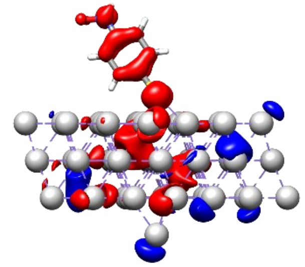

State 144
Energy: 2.685 eV
Osc.: 0.012

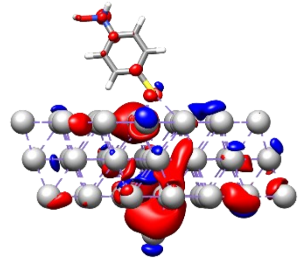

State 145
Energy: 2.692 eV
Osc.: 0.010

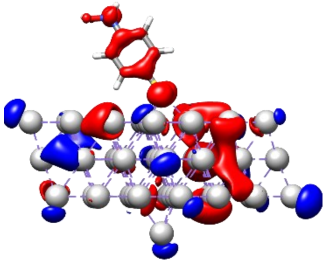

State 146
Energy: 2.709 eV
Osc.: 0.029

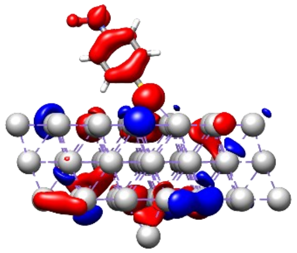

State 147
Energy: 2.718 eV
Osc.: 0.037

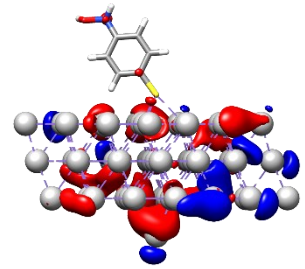

State 148
Energy: 2.725 eV
Osc.: 0.037

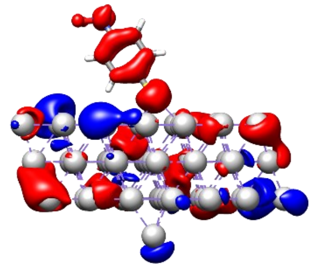

State 149
Energy: 2.741 eV
Osc.: 0.013

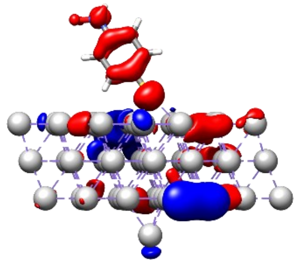

State 153
Energy: 2.770 eV
Osc.: 0.064

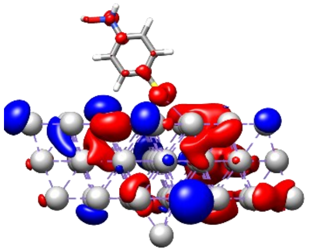

State 154
Energy: 2.778 eV
Osc.: 0.024

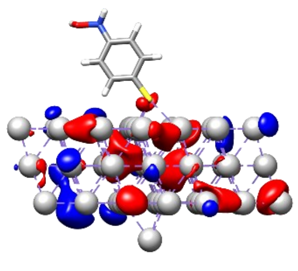

State 155
Energy: 2.783 eV
Osc.: 0.020

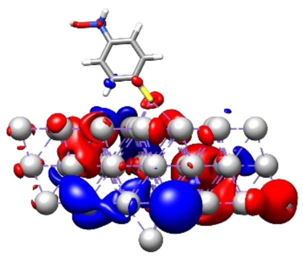

State 156
Energy: 2.798 eV
Osc.: 0.021

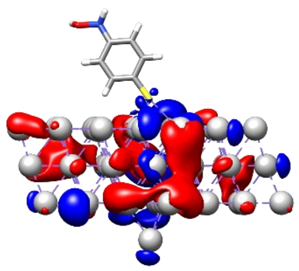

State 157
Energy: 2.812 eV
Osc.: 0.110

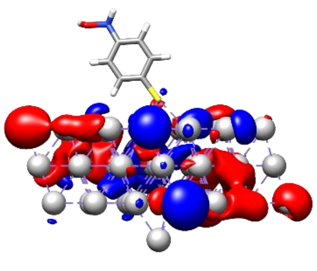

State 158
Energy: 2.815 eV
Osc.: 0.016

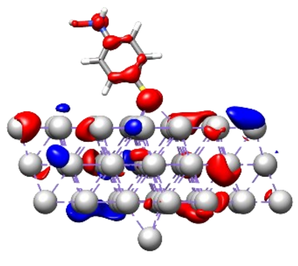

State 159
Energy: 2.831 eV
Osc.: 0.014

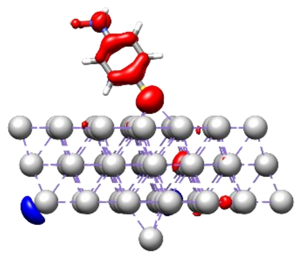

State 160
Energy: 2.837 eV
Osc.: 0.020

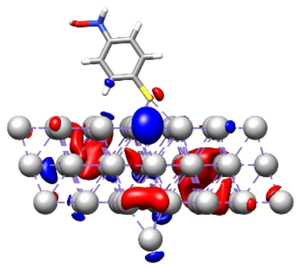

State 162
Energy: 2.848 eV
Osc.: 0.017

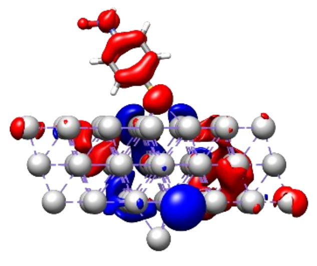

State 163
Energy: 2.858 eV
Osc.: 0.270

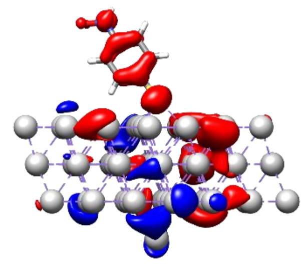

State 164
Energy: 2.871 eV
Osc.: 0.139

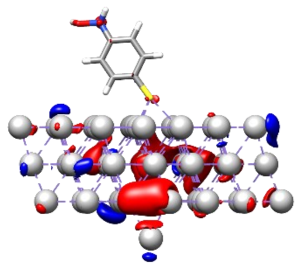

State 165
Energy: 2.884 eV
Osc.: 0.133

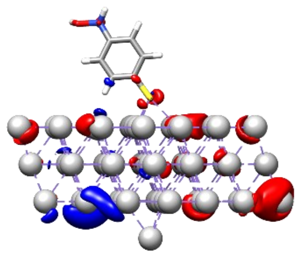

State 166
Energy: 2.887 eV
Osc.: 0.073

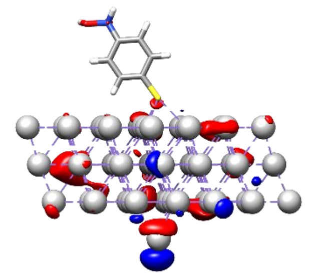

State 168
Energy: 2.908 eV
Osc.: 0.034

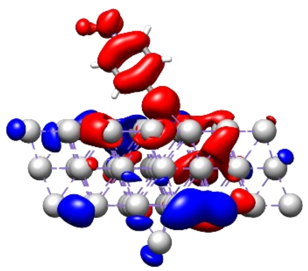

State 169
Energy: 2.909 eV
Osc.: 0.038

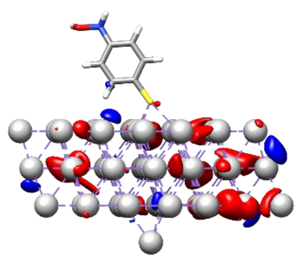

State 170
Energy: 2.914 eV
Osc.: 0.034

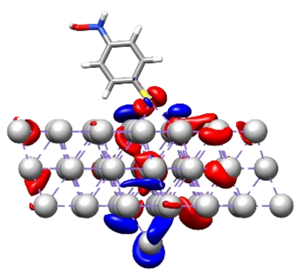

State 171
Energy: 2.929 eV
Osc.: 0.065

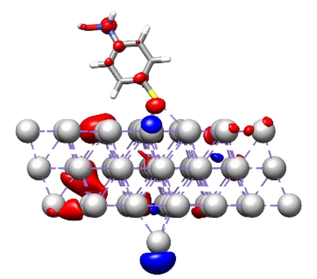

State 172
Energy: 2.935 eV
Osc.: 0.043

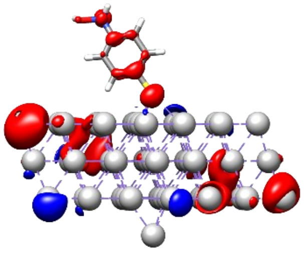

State 173
Energy: 2.942 eV
Osc.: 0.352

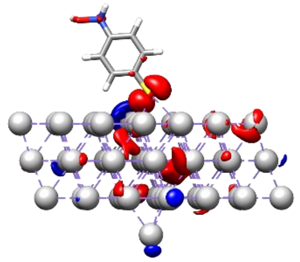

State 174
Energy: 2.947 eV
Osc.: 0.289

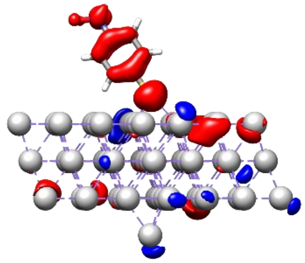

State 175
Energy: 2.956 eV
Osc.: 0.178

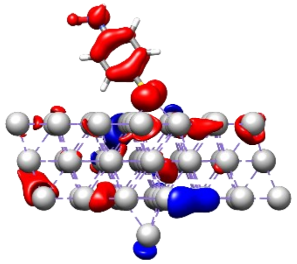

State 176
Energy: 2.963 eV
Osc.: 0.101

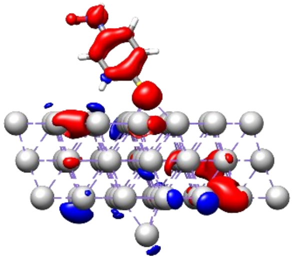

State 177
Energy: 2.970 eV
Osc.: 0.060

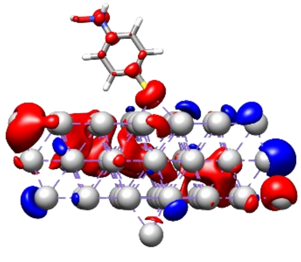

State 178
Energy: 2.978 eV
Osc.: 0.024

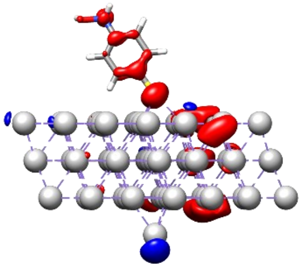

State 179
Energy: 2.987 eV
Osc.: 0.038

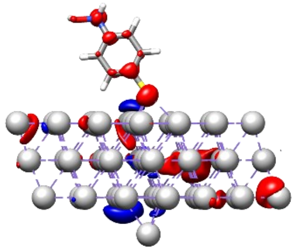

State 180
Energy: 2.994 eV
Osc.: 0.027

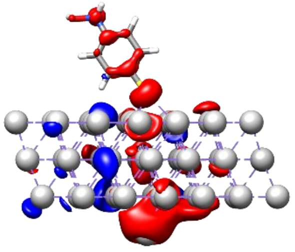

State 181
Energy: 3.003 eV
Osc.: 0.027

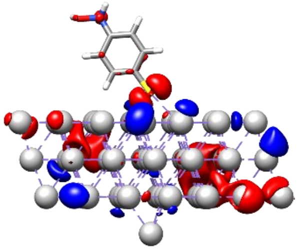

State 182
Energy: 3.011 eV
Osc.: 0.019

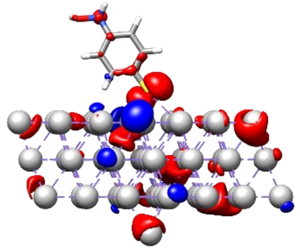

State 183
Energy: 3.023 eV
Osc.: 0.019

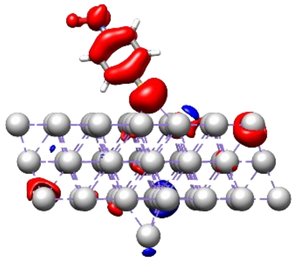

State 184
Energy: 3.025 eV
Osc.: 0.042

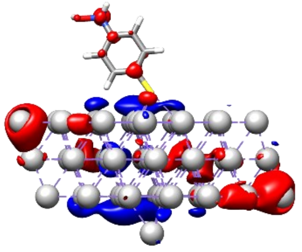

State 185
Energy: 3.029 eV
Osc.: 0.126

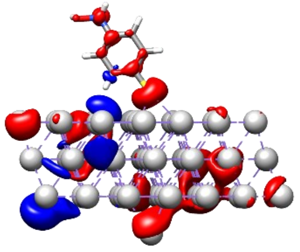

State 186
Energy: 3.044 eV
Osc.: 0.438

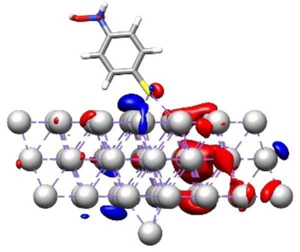

State 187
Energy: 3.050 eV
Osc.: 0.012

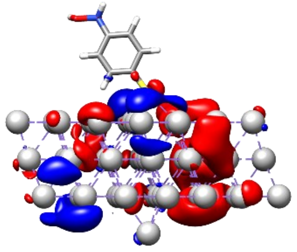

State 188
Energy: 3.059 eV
Osc.: 0.017

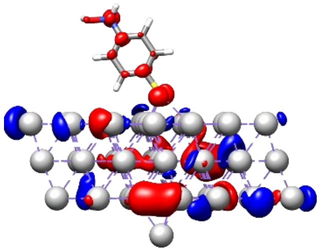

State 189
Energy: 3.068 eV
Osc.: 0.183

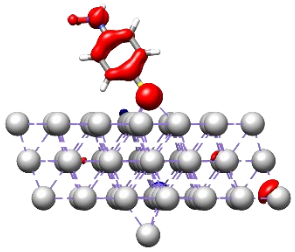

State 190
Energy: 3.072 eV
Osc.: 0.417

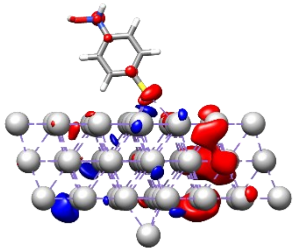

State 191
Energy: 3.076 eV
Osc.: 0.090

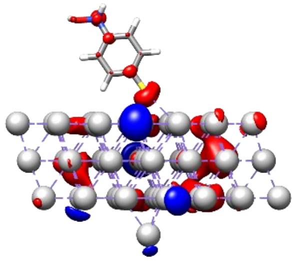

State 192
Energy: 3.087 eV
Osc.: 0.278

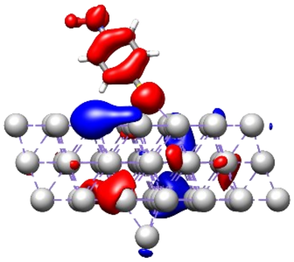

State 193
Energy: 3.093 eV
Osc.: 0.071

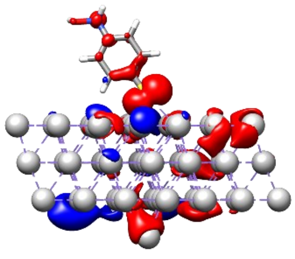

State 194
Energy: 3.099 eV
Osc.: 0.048

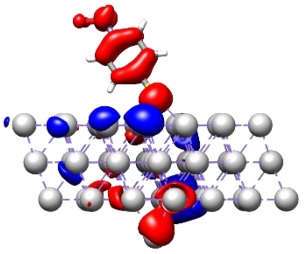

State 195
Energy: 3.102 eV
Osc.: 0.235

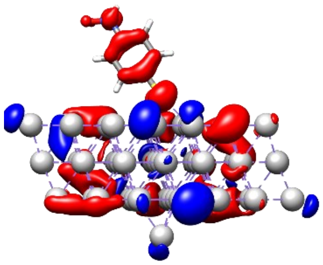

State 196
Energy: 3.106 eV
Osc.: 0.319

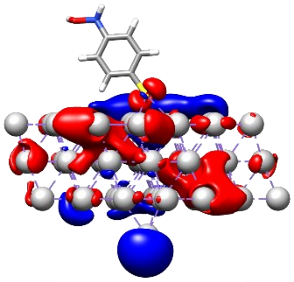

State 197
Energy: 3.112 eV
Osc.: 0.036

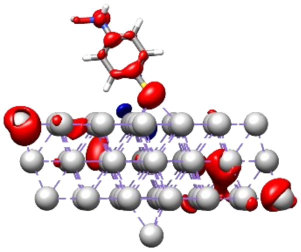

State 198
Energy: 3.127 eV
Osc.: 0.084

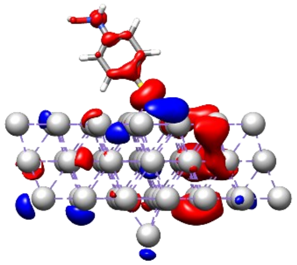

State 200
Energy: 3.135 eV
Osc.: 0.068

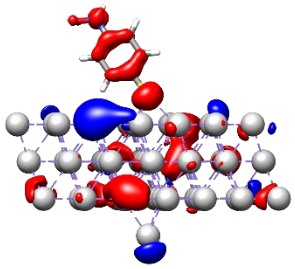

State 201
Energy: 3.144 eV
Osc.: 0.023

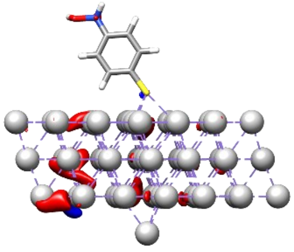

State 202
Energy: 3.155 eV
Osc.: 0.097

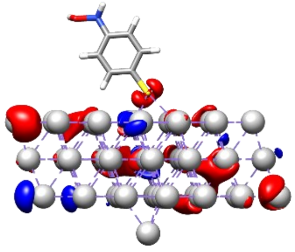

State 203
Energy: 3.156 eV
Osc.: 0.045

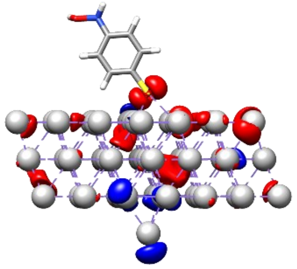

State 204
Energy: 3.164 eV
Osc.: 0.185

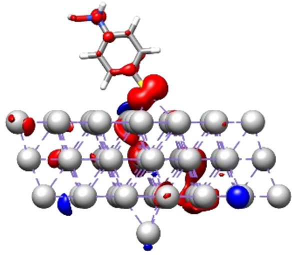

State 205
Energy: 3.172 eV
Osc.: 1.111

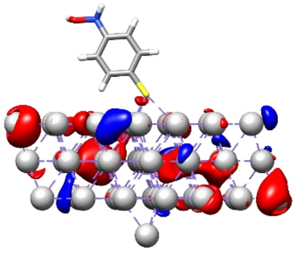

State 206
Energy: 3.176 eV
Osc.: 0.086

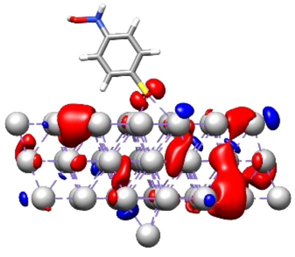

State 207
Energy: 3.182 eV
Osc.: 0.101

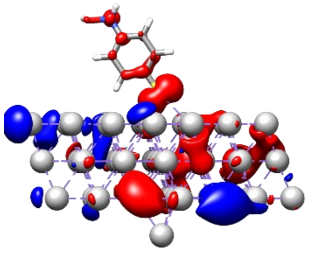

State 208
Energy: 3.192 eV
Osc.: 0.316

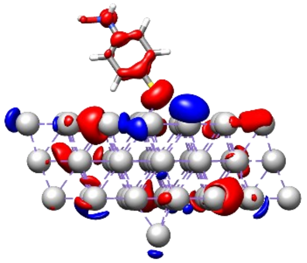

State 209
Energy: 3.200 eV
Osc.: 0.012

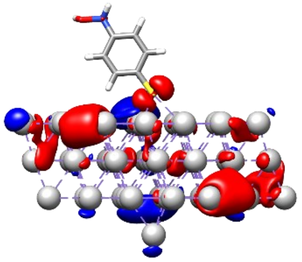

State 210
Energy: 3.203 eV
Osc.: 0.028

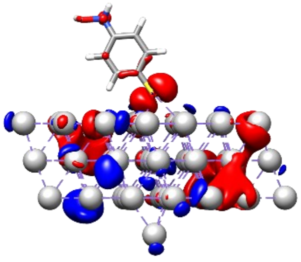

State 211
Energy: 3.209 eV
Osc.: 0.044

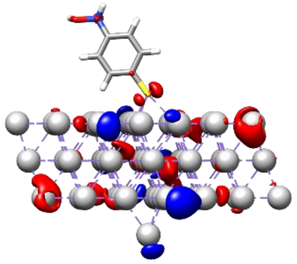

State 212
Energy: 3.215 eV
Osc.: 0.316

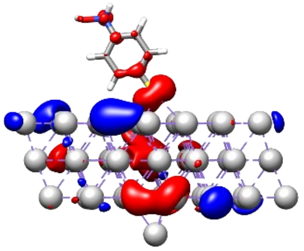

State 213
Energy: 3.228 eV
Osc.: 0.123

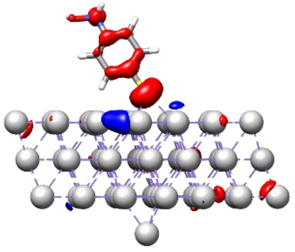

State 215
Energy: 3.250 eV
Osc.: 0.342

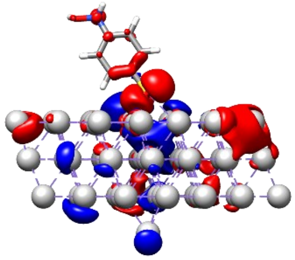

State 217
Energy: 3.266 eV
Osc.: 0.053

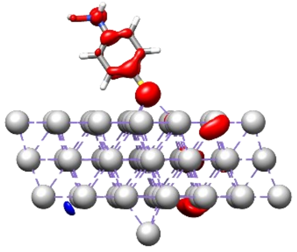

State 218
Energy: 3.271 eV
Osc.: 0.019

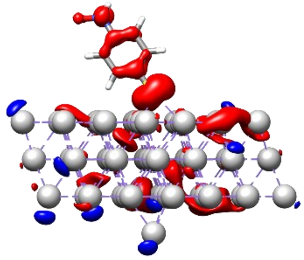

State 219
Energy: 3.283 eV
Osc.: 0.029

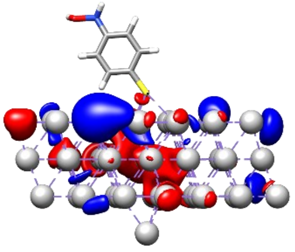

State 220
Energy: 3.287 eV
Osc.: 0.050

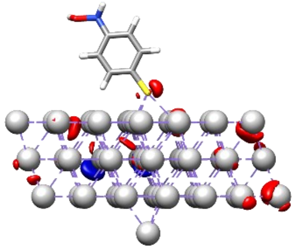

State 221
Energy: 3.296 eV
Osc.: 0.099

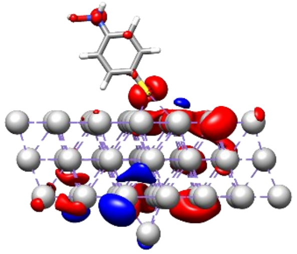

State 222
Energy: 3.300 eV
Osc.: 0.101

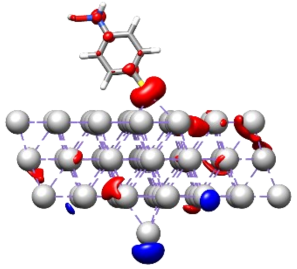

State 224
Energy: 3.313 eV
Osc.: 0.107

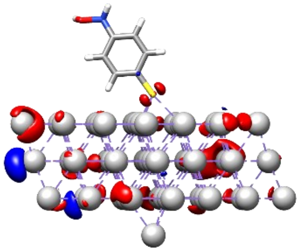<br>State 225<br>Energy: 3.318 eV<br>Osc.: 0.039	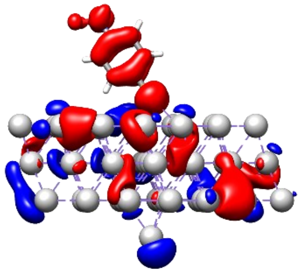<br>State 227<br>Energy: 3.331 eV<br>Osc.: 0.073		

Table S7: Charge density differences (CDDs) illustrating the nature of the low-lying bright excitations of intermediate C. Charge transfer takes place from red to blue

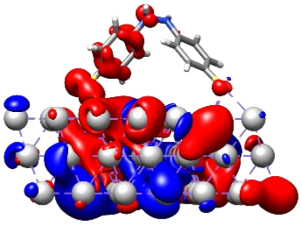<br>State 48<br>Energy: 1.652 eV<br>Osc.: 0.018	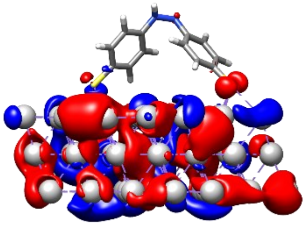<br>State 52<br>Energy: 1.702 eV<br>Osc.: 0.014	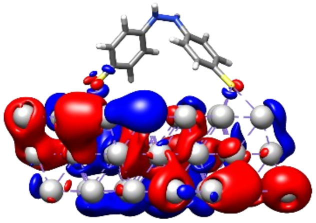<br>State 63<br>Energy: 1.885 eV<br>Osc.: 0.017	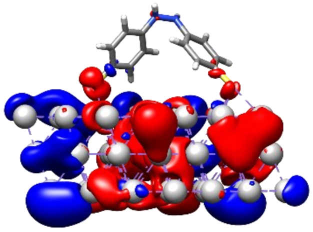<br>State 68<br>Energy: 1.939 eV<br>Osc.: 0.010
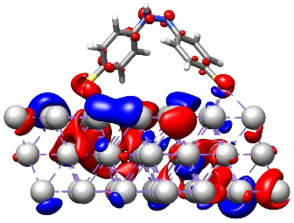<br>State 71<br>Energy: 1.975 eV<br>Osc.: 0.016	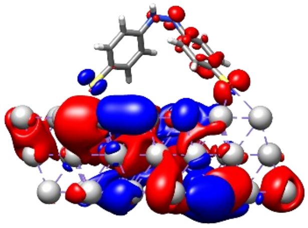<br>State 75<br>Energy: 2.026 eV<br>Osc.: 0.011	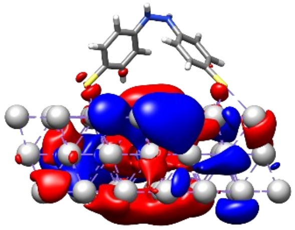<br>State 77<br>Energy: 2.048 eV<br>Osc.: 0.013	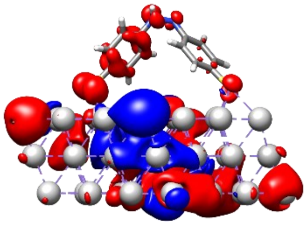<br>State 79<br>Energy: 2.071 eV<br>Osc.: 0.013

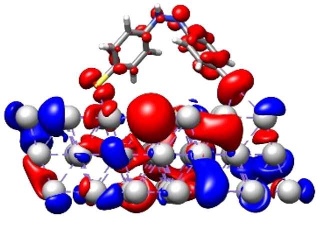<br>State 81<br>Energy: 2.107 eV<br>Osc.: 0.015	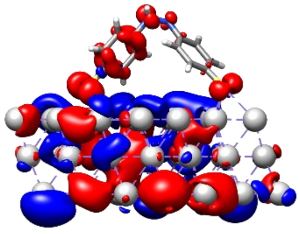<br>State 82<br>Energy: 2.117 eV<br>Osc.: 0.029	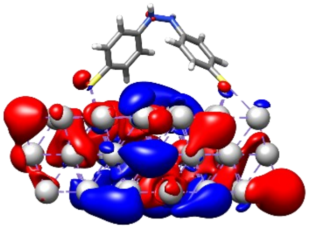<br>State 85<br>Energy: 2.164 eV<br>Osc.: 0.031	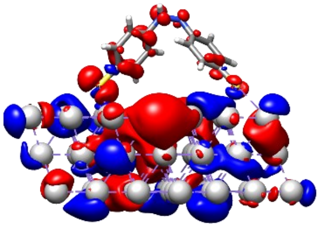<br>State 86<br>Energy: 2.187 eV<br>Osc.: 0.021
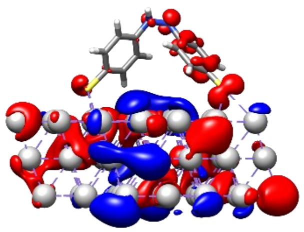<br>State 87<br>Energy: 2.188 eV<br>Osc.: 0.048	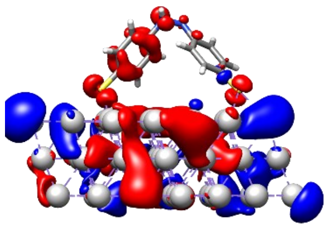<br>State 89<br>Energy: 2.219 eV<br>Osc.: 0.033	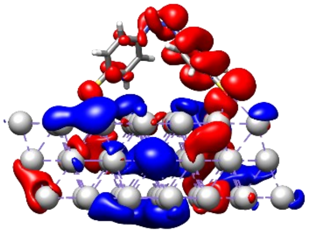<br>State 93<br>Energy: 2.274 eV<br>Osc.: 0.015	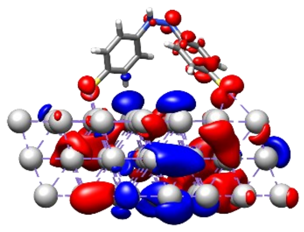<br>State 96<br>Energy: 2.295 eV<br>Osc.: 0.016
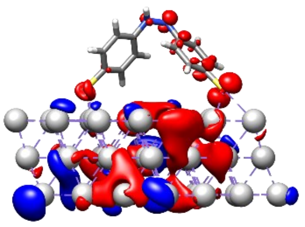<br>State 100<br>Energy: 2.329 eV<br>Osc.: 0.058	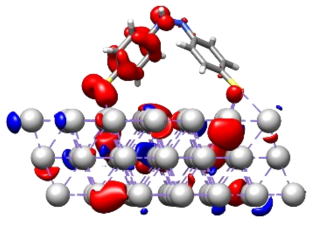<br>State 103<br>Energy: 2.361 eV<br>Osc.: 0.018	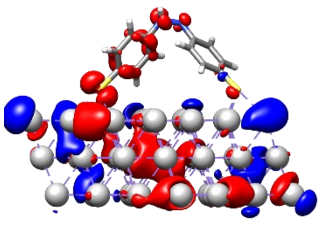<br>State 105<br>Energy: 2.376 eV<br>Osc.: 0.016	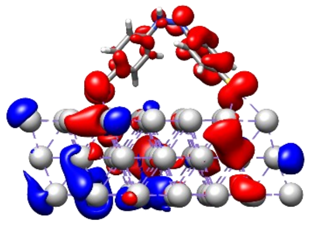<br>State 107<br>Energy: 2.401 eV<br>Osc.: 0.010
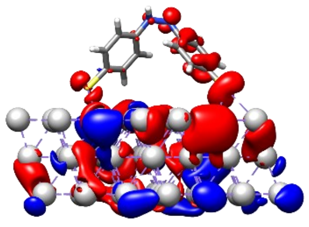<br>State 109<br>Energy: 2.423 eV<br>Osc.: 0.014	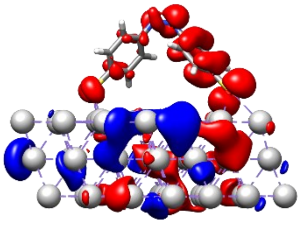<br>State 111<br>Energy: 2.441 eV<br>Osc.: 0.024	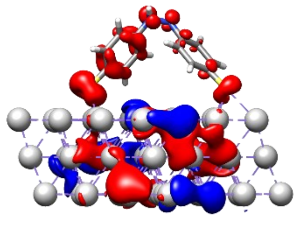<br>State 112<br>Energy: 2.453 eV<br>Osc.: 0.067	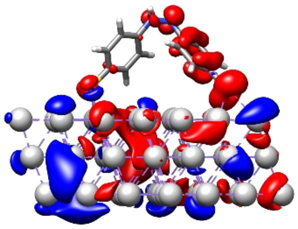<br>State 113<br>Energy: 2.458 eV<br>Osc.: 0.014

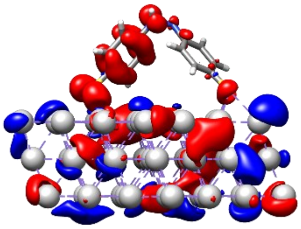

State 115
Energy: 2.482 eV
Osc.: 0.076

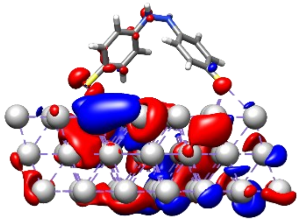

State 116
Energy: 2.486 eV
Osc.: 0.022

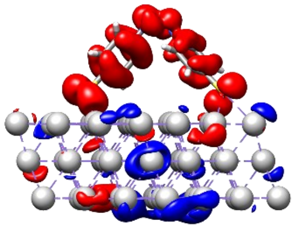

State 121
Energy: 2.542 eV
Osc.: 0.020

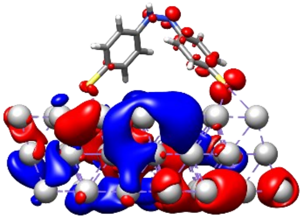

State 124
Energy: 2.566 eV
Osc.: 0.099

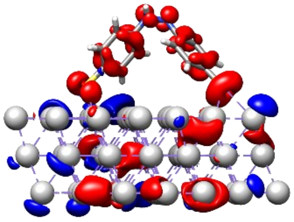

State 126
Energy: 2.583 eV
Osc.: 0.023

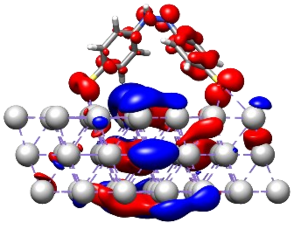

State 127
Energy: 2.588 eV
Osc.: 0.010

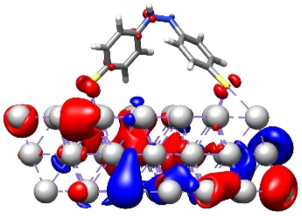

State 128
Energy: 2.596 eV
Osc.: 0.067

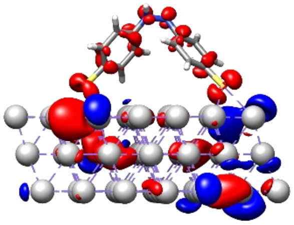

State 130
Energy: 2.617 eV
Osc.: 0.037

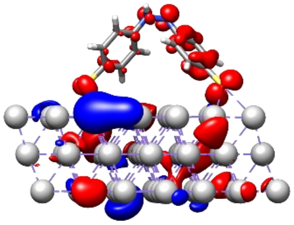

State 132
Energy: 2.629 eV
Osc.: 0.123

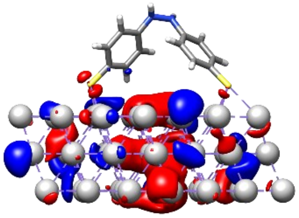

State 133
Energy: 2.647 eV
Osc.: 0.013

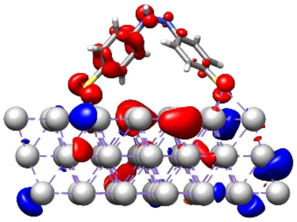

State 134
Energy: 2.651 eV
Osc.: 0.340

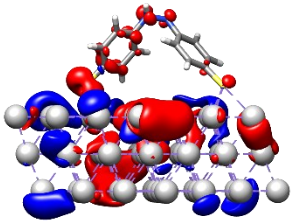

State 136
Energy: 2.669 eV
Osc.: 0.010

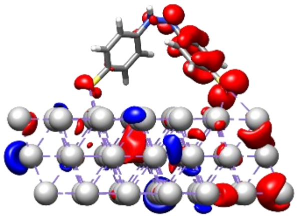

State 137
Energy: 2.674 eV
Osc.: 0.013

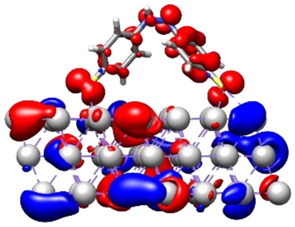

State 138
Energy: 2.686 eV
Osc.: 0.037

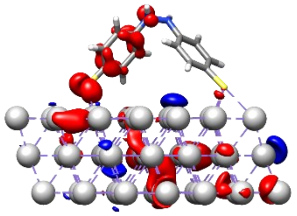

State 139
Energy: 2.692 eV
Osc.: 0.039

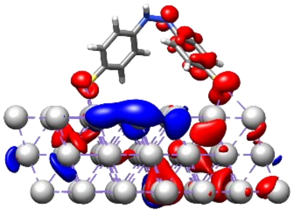

State 140
Energy: 2.699 eV
Osc.: 0.036

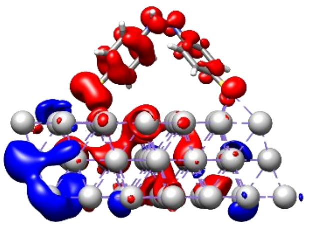

State 141
Energy: 2.715 eV
Osc.: 0.022

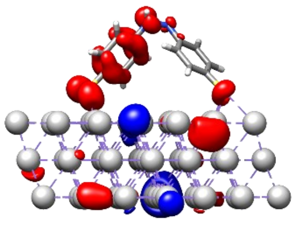

State 142
Energy: 2.726 eV
Osc.: 0.108

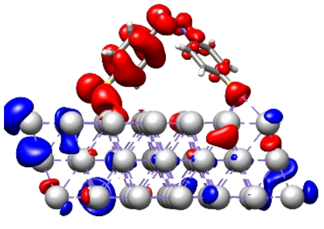

State 143
Energy: 2.736 eV
Osc.: 0.023

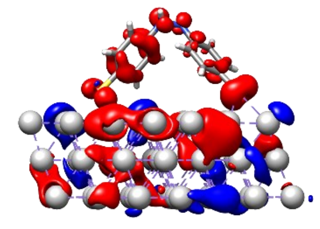

State 145
Energy: 2.752 eV
Osc.: 0.057

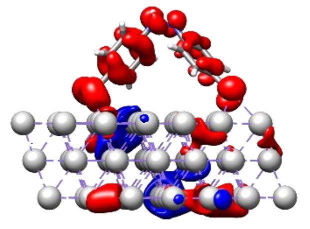

State 146
Energy: 2.757 eV
Osc.: 0.021

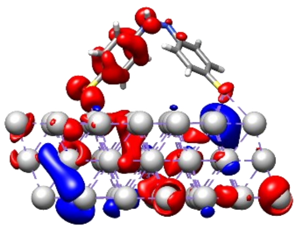

State 147
Energy: 2.772 eV
Osc.: 0.012

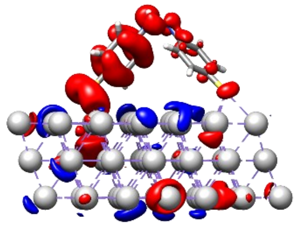

State 149
Energy: 2.786 eV
Osc.: 0.015

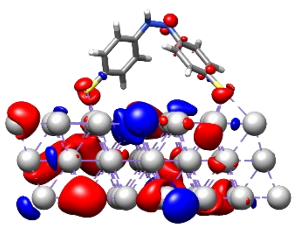

State 150
Energy: 2.791 eV
Osc.: 0.250

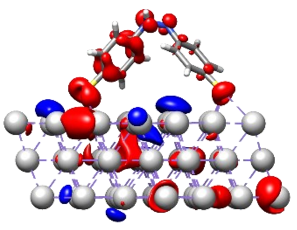

State 151
Energy: 2.795 eV
Osc.: 0.014

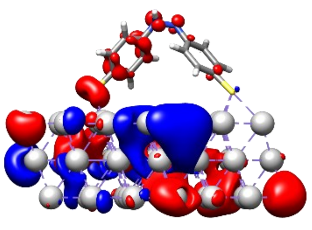

State 152
Energy: 2.799 eV
Osc.: 0.107

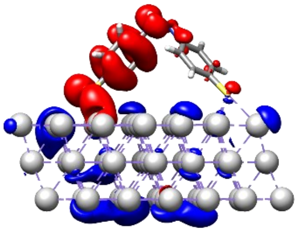

State 153
Energy: 2.807 eV
Osc.: 0.034

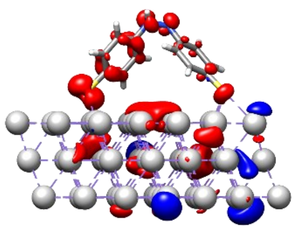

State 154
Energy: 2.817 eV
Osc.: 0.036

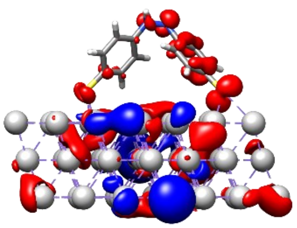

State 156
Energy: 2.848 eV
Osc.: 0.084

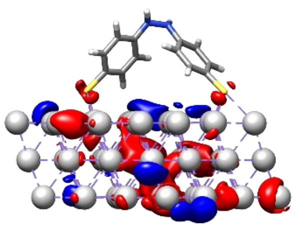

State 157
Energy: 2.856 eV
Osc.: 0.072

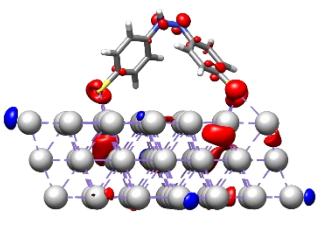

State 159
Energy: 2.874 eV
Osc.: 0.032

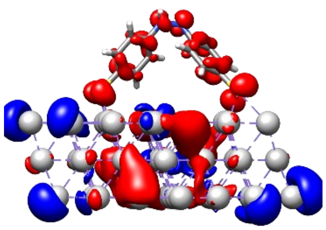

State 160
Energy: 2.888 eV
Osc.: 0.014

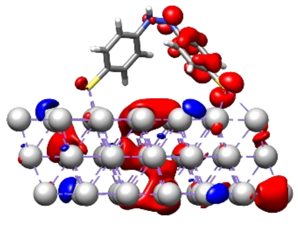

State 161
Energy: 2.894 eV
Osc.: 0.145

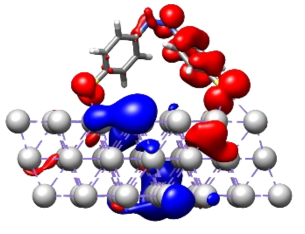

State 162
Energy: 2.898 eV
Osc.: 0.017

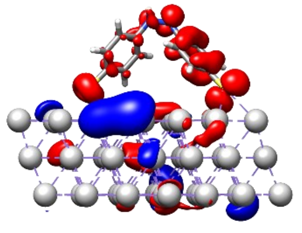

State 163
Energy: 2.900 eV
Osc.: 0.025

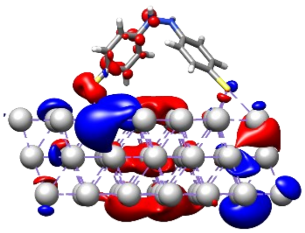

State 164
Energy: 2.913 eV
Osc.: 0.040

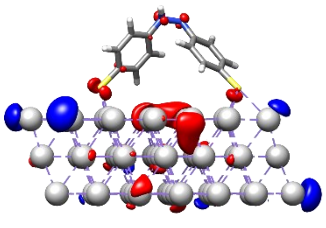

State 165
Energy: 2.916 eV
Osc.: 0.060

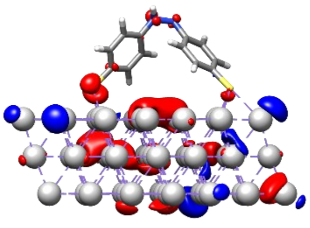

State 166
Energy: 2.936 eV
Osc.: 0.017

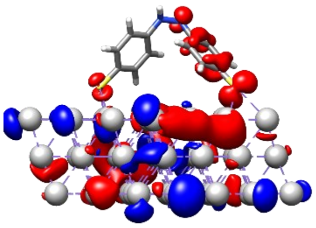

State 167
Energy: 2.943 eV
Osc.: 0.068

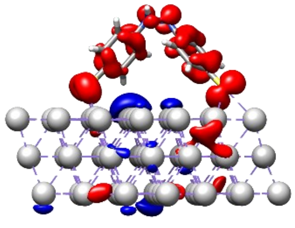

State 168
Energy: 2.949 eV
Osc.: 0.616

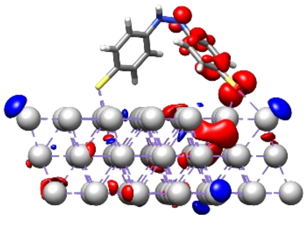

State 169
Energy: 2.954 eV
Osc.: 0.065

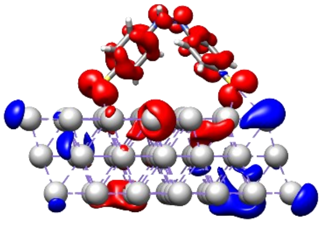

State 170
Energy: 2.954 eV
Osc.: 0.486

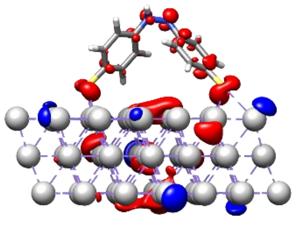

State 171
Energy: 2.970 eV
Osc.: 0.061

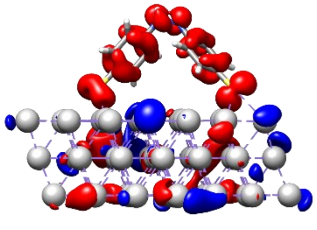

State 172
Energy: 2.981 eV
Osc.: 0.113

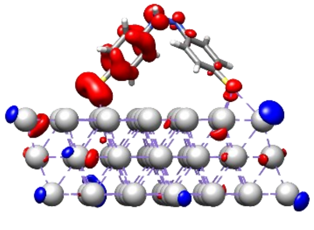

State 173
Energy: 2.984 eV
Osc.: 0.451

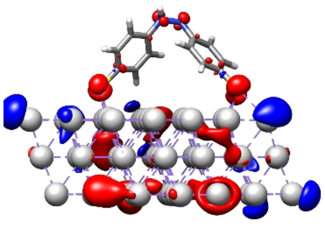

State 174
Energy: 2.993 eV
Osc.: 0.049

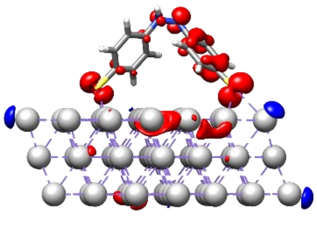

State 175
Energy: 2.995 eV
Osc.: 0.012

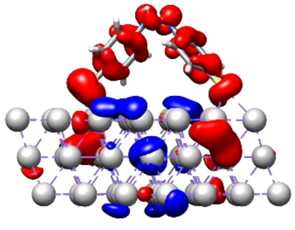

State 176
Energy: 3.004 eV
Osc.: 0.015

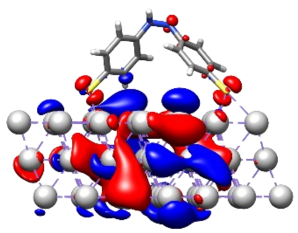

State 177
Energy: 3.016 eV
Osc.: 0.070

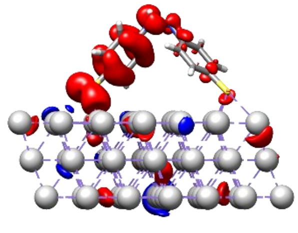

State 178
Energy: 3.022 eV
Osc.: 0.408

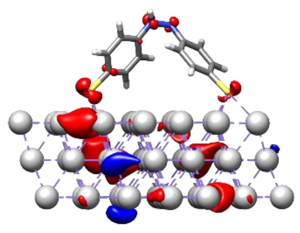

State 179
Energy: 3.035 eV
Osc.: 0.044

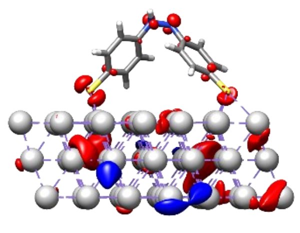

State 180
Energy: 3.045 eV
Osc.: 0.300

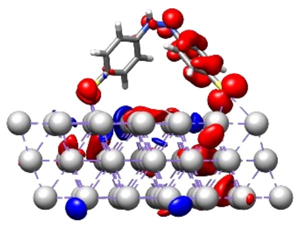

State 181
Energy: 3.051 eV
Osc.: 0.110

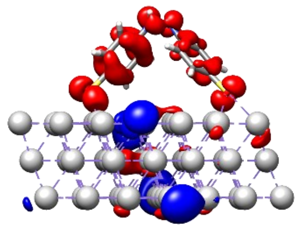

State 182
Energy: 3.054 eV
Osc.: 0.056

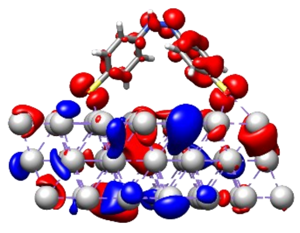

State 183
Energy: 3.066 eV
Osc.: 0.033

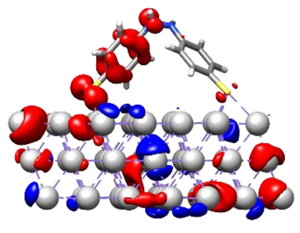

State 184
Energy: 3.066 eV
Osc.: 0.187

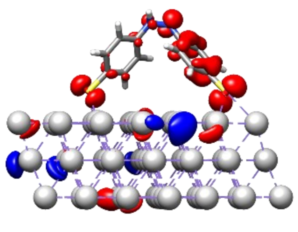

State 185
Energy: 3.067 eV
Osc.: 0.211

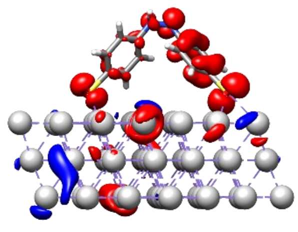

State 186
Energy: 3.088 eV
Osc.: 0.321

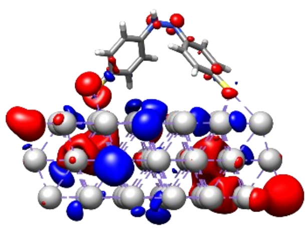

State 187
Energy: 3.093 eV
Osc.: 0.021

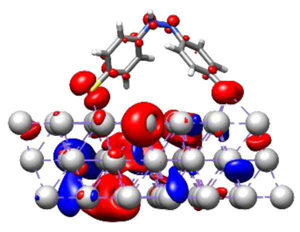

State 188
Energy: 3.103 eV
Osc.: 0.053

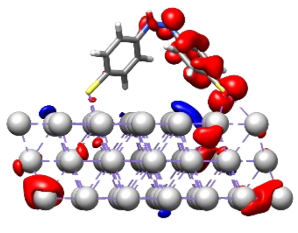

State 189
Energy: 3.110 eV
Osc.: 0.031

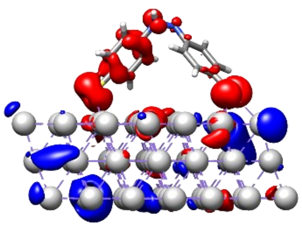

State 190
Energy: 3.118 eV
Osc.: 0.070

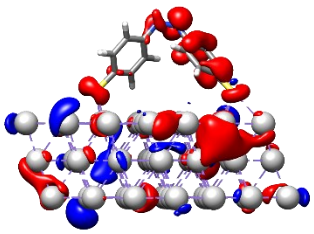

State 192
Energy: 3.136 eV
Osc.: 0.049

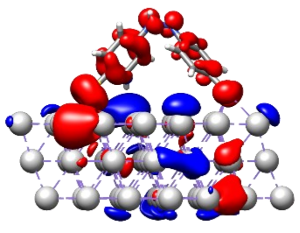

State 193
Energy: 3.143 eV
Osc.: 0.021

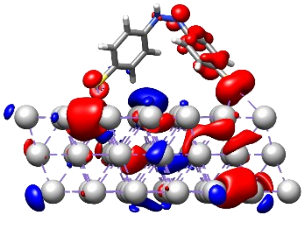

State 194
Energy: 3.148 eV
Osc.: 0.038

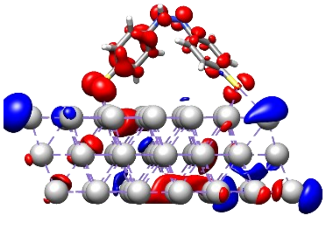

State 195
Energy: 3.151 eV
Osc.: 0.049

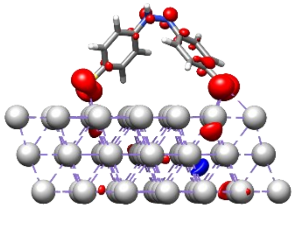

State 197
Energy: 3.166 eV
Osc.: 0.025

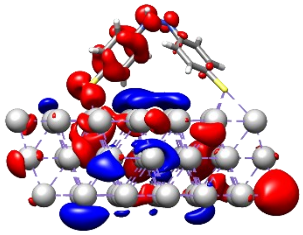

State 198
Energy: 3.168 eV
Osc.: 0.010

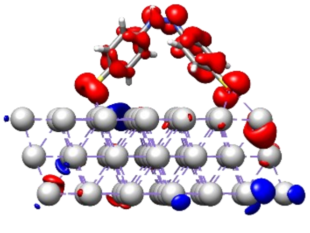

State 199
Energy: 3.184 eV
Osc.: 0.013

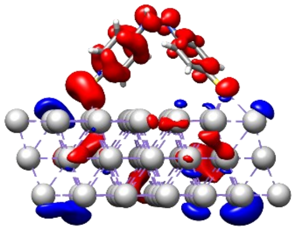

State 200
Energy: 3.186 eV
Osc.: 0.015

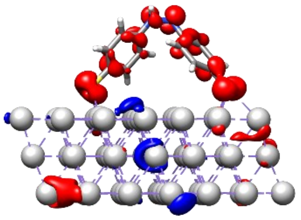

State 201
Energy: 3.193 eV
Osc.: 0.026

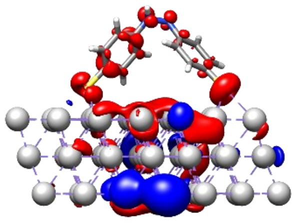

State 202
Energy: 3.203 eV
Osc.: 0.296

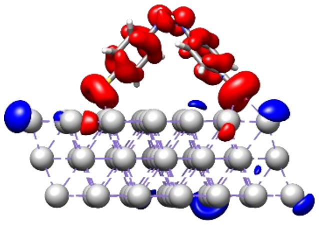

State 205
Energy: 3.222 eV
Osc.: 0.088

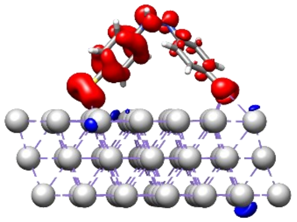

State 206
Energy: 3.235 eV
Osc.: 0.063

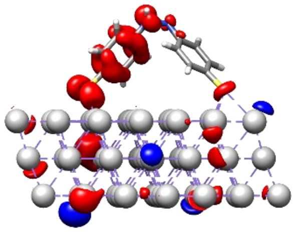

State 208
Energy: 3.252 eV
Osc.: 0.321

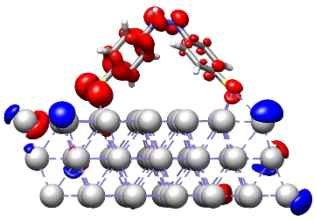

State 209
Energy: 3.257 eV
Osc.: 0.018

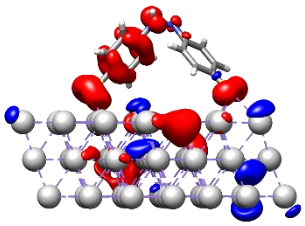

State 210
Energy: 3.259 eV
Osc.: 0.041

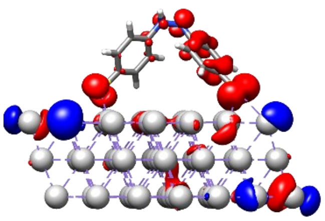

State 212
Energy: 3.268 eV
Osc.: 0.036

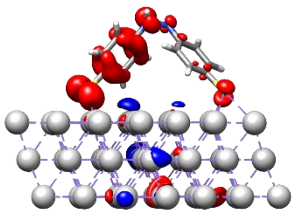

State 213
Energy: 3.277 eV
Osc.: 0.012

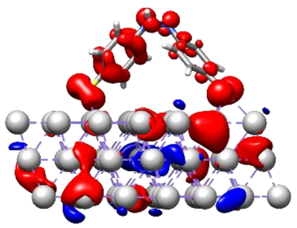

State 214
Energy: 3.281 eV
Osc.: 0.157

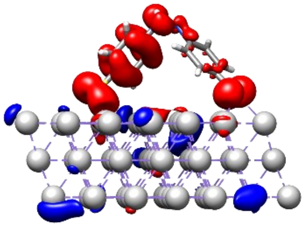<br>State 215<br>Energy: 3.284 eV<br>Osc.: 0.085	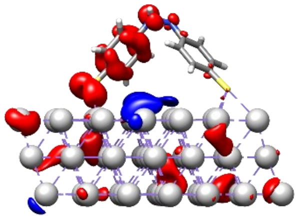<br>State 217<br>Energy: 3.301 eV<br>Osc.: 0.039	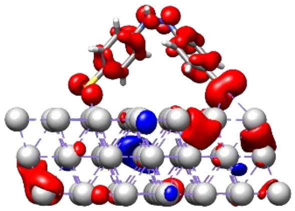<br>State 218<br>Energy: 3.311 eV<br>Osc.: 0.044	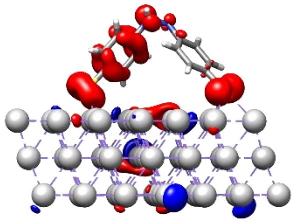<br>State 219<br>Energy: 3.321 eV<br>Osc.: 0.171
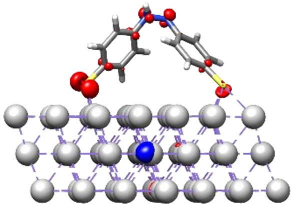<br>State 220<br>Energy: 3.324 eV<br>Osc.: 0.146	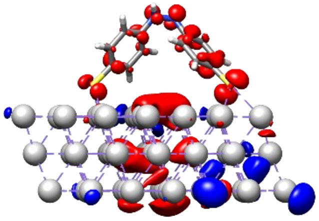<br>State 221<br>Energy: 3.331 eV<br>Osc.: 0.037	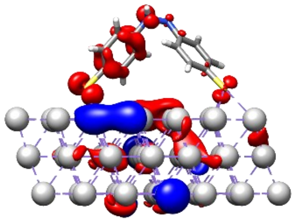<br>State 222<br>Energy: 3.335 eV<br>Osc.: 0.053	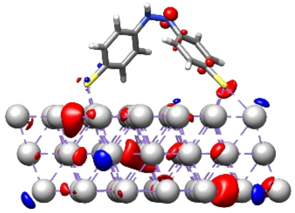<br>State 223<br>Energy: 3.338 eV<br>Osc.: 0.125

Table S8: Charge density differences (CDDs) illustrating the nature of the low-lying bright excitations of intermediate D. Charge transfer takes place from red to blue

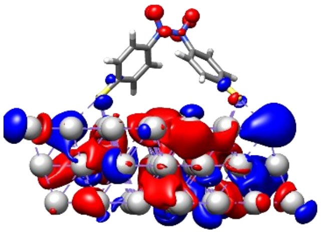<br>State 44<br>Energy: 1.590 eV<br>Osc.: 0.010	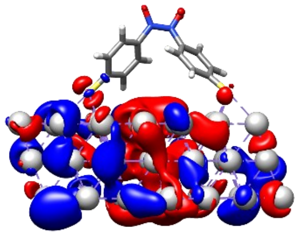<br>State 47<br>Energy: 1.637 eV<br>Osc.: 0.018	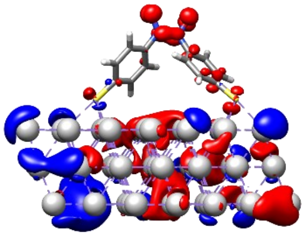<br>State 57<br>Energy: 1.796 eV<br>Osc.: 0.013	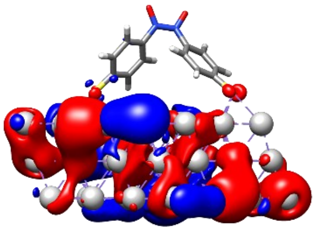<br>State 63<br>Energy: 1.893 eV<br>Osc.: 0.010

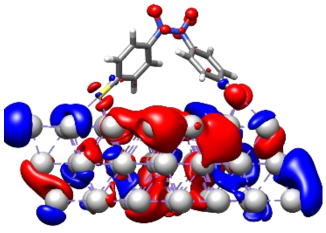

State 66
Energy: 1.927 eV
Osc.: 0.016

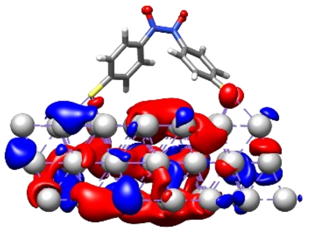

State 68
Energy: 1.941 eV
Osc.: 0.013

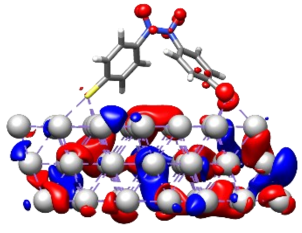

State 73
Energy: 2.019 eV
Osc.: 0.026

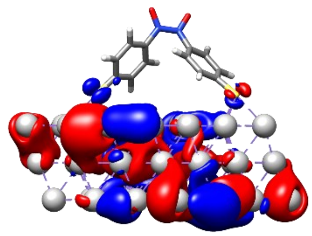

State 74
Energy: 2.026 eV
Osc.: 0.012

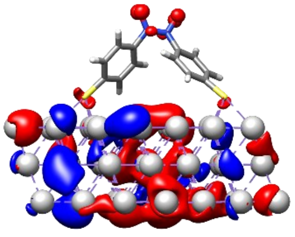

State 80
Energy: 2.091 eV
Osc.: 0.014

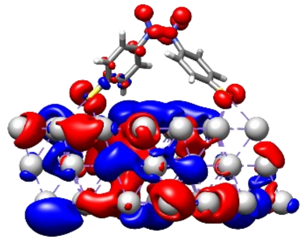

State 82
Energy: 2.131 eV
Osc.: 0.021

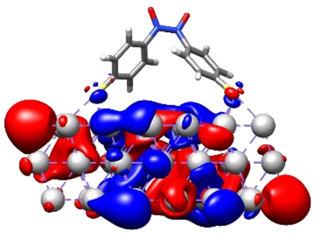

State 85
Energy: 2.172 eV
Osc.: 0.038

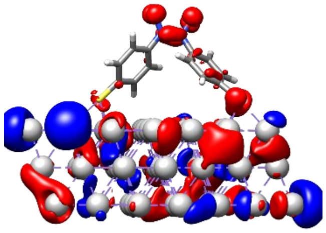

State 86
Energy: 2.189 eV
Osc.: 0.025

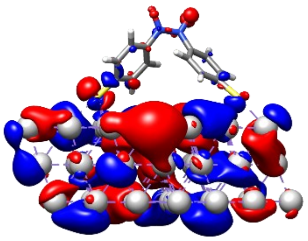

State 88
Energy: 2.223 eV
Osc.: 0.015

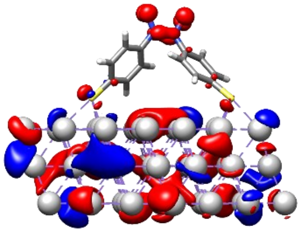

State 89
Energy: 2.238 eV
Osc.: 0.035

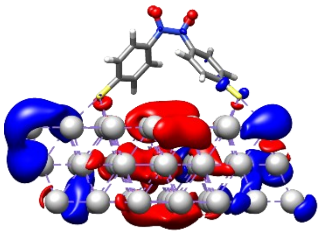

State 91
Energy: 2.247 eV
Osc.: 0.040

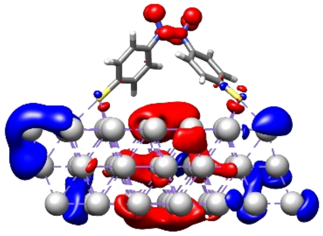

State 92
Energy: 2.264 eV
Osc.: 0.011

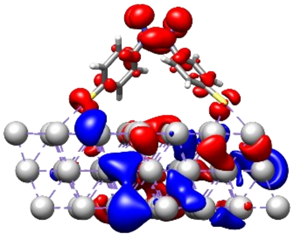

State 93
Energy: 2.287 eV
Osc.: 0.018

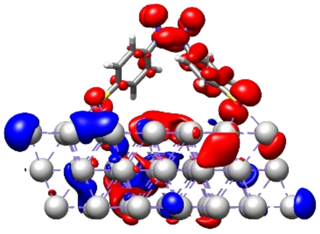

State 100
Energy: 2.338 eV
Osc.: 0.037

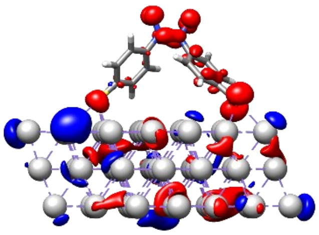

State 101
Energy: 2.353 eV
Osc.: 0.014

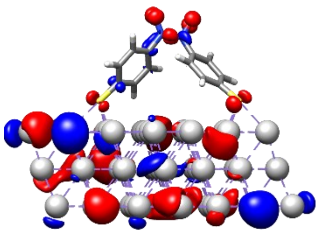

State 102
Energy: 2.362 eV
Osc.: 0.018

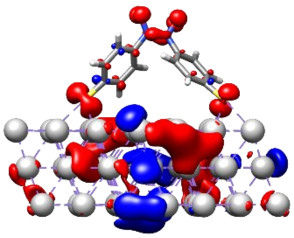

State 105
Energy: 2.399 eV
Osc.: 0.023

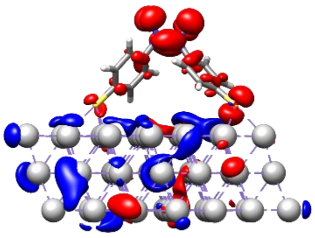

State 108
Energy: 2.427 eV
Osc.: 0.016

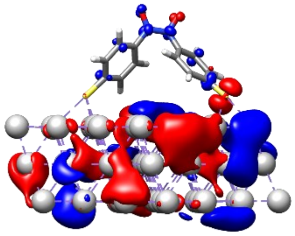

State 110
Energy: 2.445 eV
Osc.: 0.014

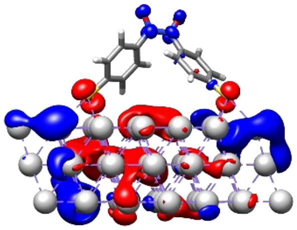

State 112
Energy: 2.461 eV
Osc.: 0.092

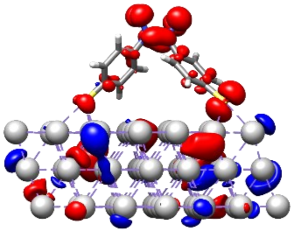

State 113
Energy: 2.478 eV
Osc.: 0.015

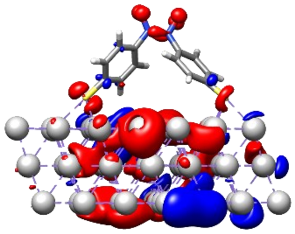

State 114
Energy: 2.482 eV
Osc.: 0.015

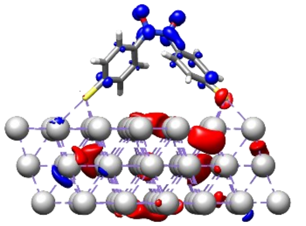

State 116
Energy: 2.503 eV
Osc.: 0.121

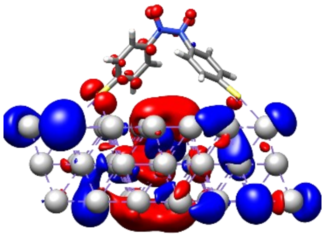

State 117
Energy: 2.510 eV
Osc.: 0.023

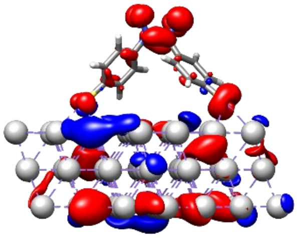

State 119
Energy: 2.538 eV
Osc.: 0.019

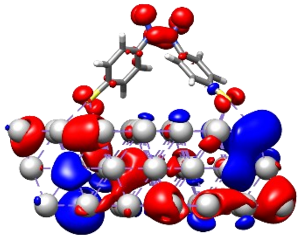

State 120
Energy: 2.547 eV
Osc.: 0.034

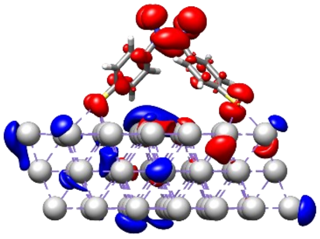

State 121
Energy: 2.560 eV
Osc.: 0.058

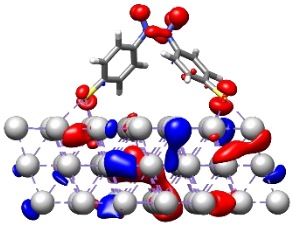

State 122
Energy: 2.573 eV
Osc.: 0.016

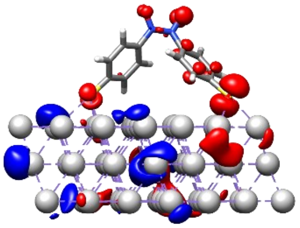

State 123
Energy: 2.581 eV
Osc.: 0.010

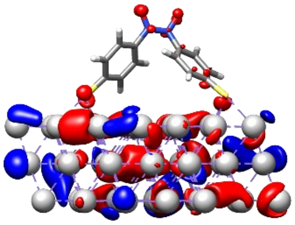

State 126
Energy: 2.603 eV
Osc.: 0.011

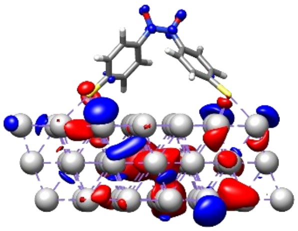

State 129
Energy: 2.615 eV
Osc.: 0.016

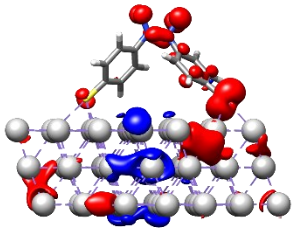

State 130
Energy: 2.629 eV
Osc.: 0.064

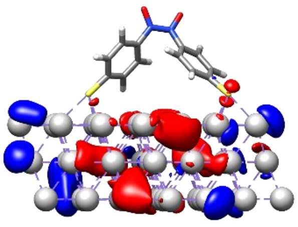

State 131
Energy: 2.633 eV
Osc.: 0.027

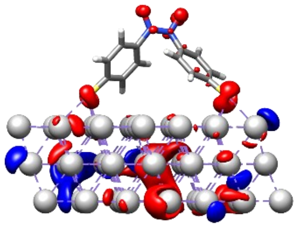

State 132
Energy: 2.650 eV
Osc.: 0.056

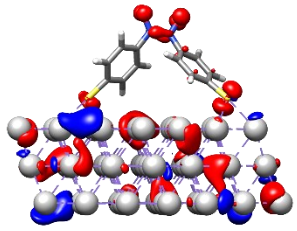

State 133
Energy: 2.653 eV
Osc.: 0.058

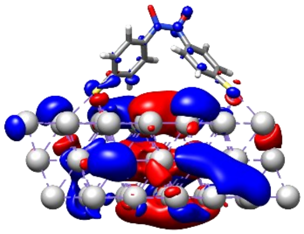

State 134
Energy: 2.662 eV
Osc.: 0.022

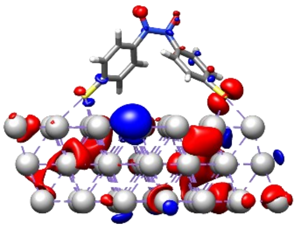

State 135
Energy: 2.670 eV
Osc.: 0.025

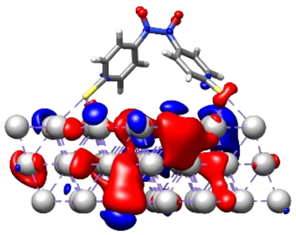

State 136
Energy: 2.686 eV
Osc.: 0.045

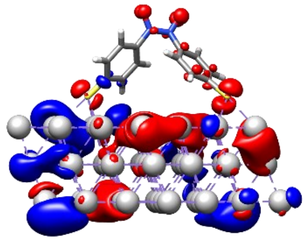

State 137
Energy: 2.693 eV
Osc.: 0.073

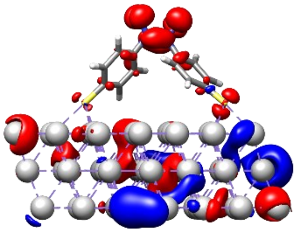

State 138
Energy: 2.708 eV
Osc.: 0.016

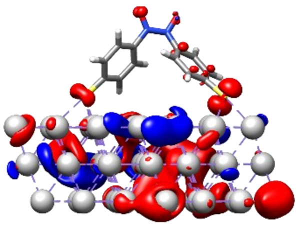

State 140
Energy: 2.730 eV
Osc.: 0.069

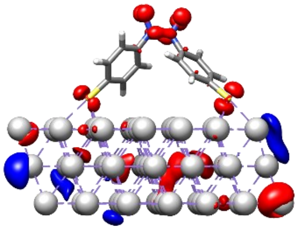

State 141
Energy: 2.734 eV
Osc.: 0.081

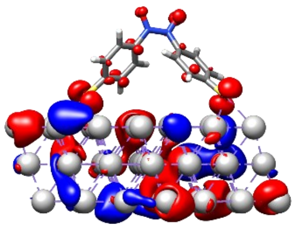

State 142
Energy: 2.741 eV
Osc.: 0.019

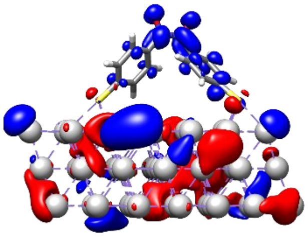

State 143
Energy: 2.749 eV
Osc.: 0.095

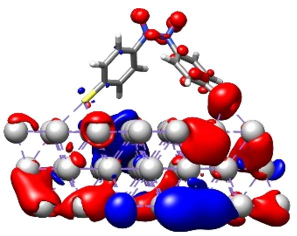

State 144
Energy: 2.758 eV
Osc.: 0.127

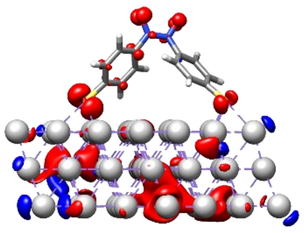

State 145
Energy: 2.770 eV
Osc.: 0.046

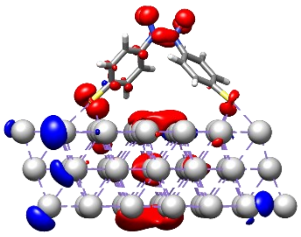

State 146
Energy: 2.777 eV
Osc.: 0.055

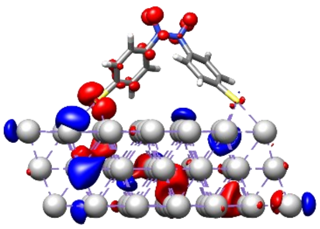

State 147
Energy: 2.782 eV
Osc.: 0.030

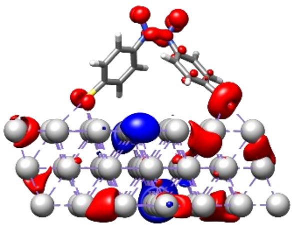<br>State 149<br>Energy: 2.797 eV<br>Osc.: 0.119	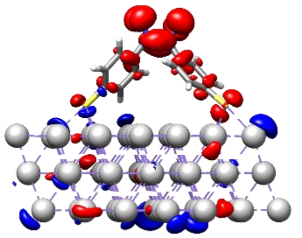<br>State 150<br>Energy: 2.803 eV<br>Osc.: 0.084	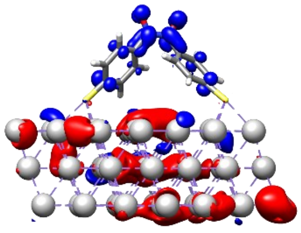<br>State 153<br>Energy: 2.839 eV<br>Osc.: 0.015	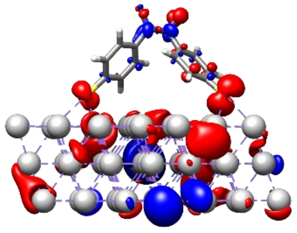<br>State 156<br>Energy: 2.858 eV<br>Osc.: 0.019
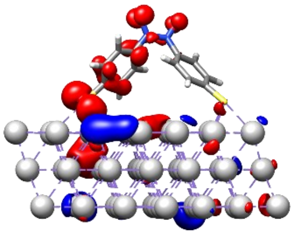<br>State 157<br>Energy: 2.866 eV<br>Osc.: 0.267	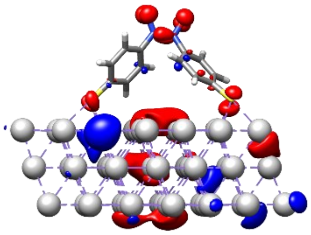<br>State 158<br>Energy: 2.881 eV<br>Osc.: 0.084	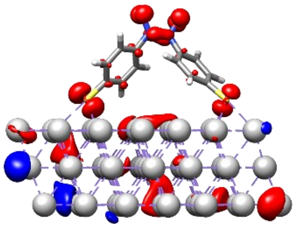<br>State 159<br>Energy: 2.883 eV<br>Osc.: 0.254	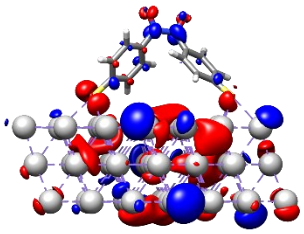<br>State 161<br>Energy: 2.908 eV<br>Osc.: 0.046
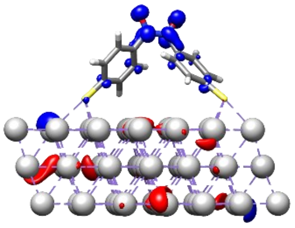<br>State 163<br>Energy: 2.914 eV<br>Osc.: 0.011	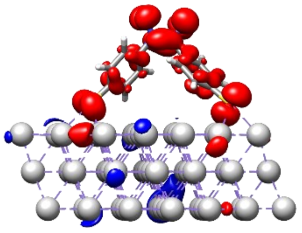<br>State 164<br>Energy: 2.920 eV<br>Osc.: 0.138	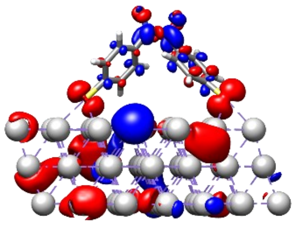<br>State 165<br>Energy: 2.923 eV<br>Osc.: 0.178	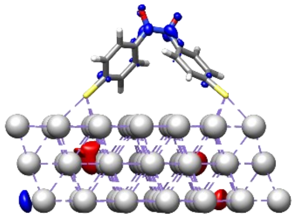<br>State 166<br>Energy: 2.925 eV<br>Osc.: 0.274
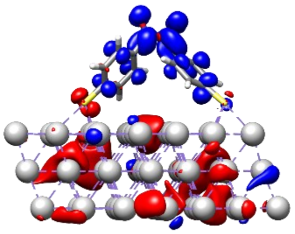<br>State 167<br>Energy: 2.932 eV<br>Osc.: 0.022	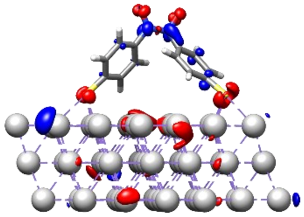<br>State 168<br>Energy: 2.944 eV<br>Osc.: 0.015	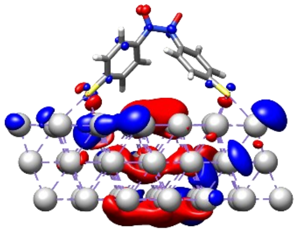<br>State 169<br>Energy: 2.953 eV<br>Osc.: 0.020	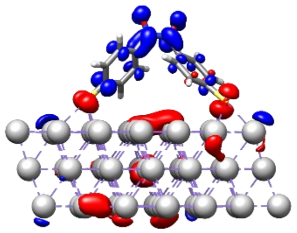<br>State 170<br>Energy: 2.960 eV<br>Osc.: 0.382

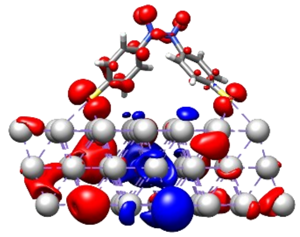

State 172
Energy: 2.975 eV
Osc.: 0.015

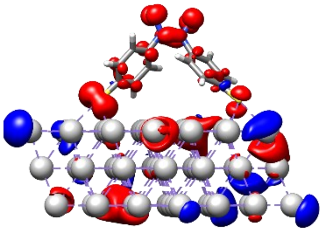

State 173
Energy: 2.994 eV
Osc.: 0.214

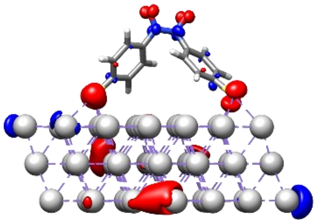

State 174
Energy: 2.997 eV
Osc.: 0.233

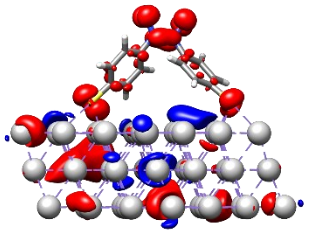

State 176
Energy: 3.017 eV
Osc.: 0.103

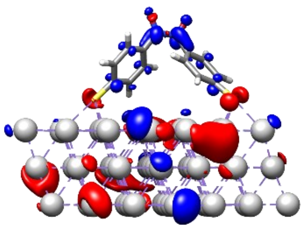

State 177
Energy: 3.026 eV
Osc.: 0.057

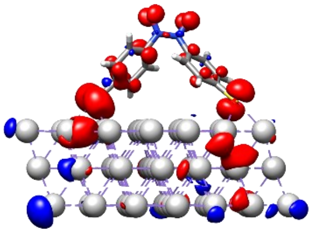

State 179
Energy: 3.034 eV
Osc.: 0.058

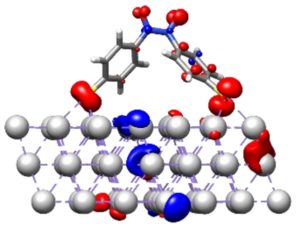

State 181
Energy: 3.046 eV
Osc.: 0.014

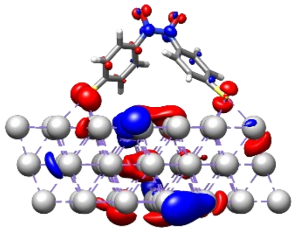

State 182
Energy: 3.054 eV
Osc.: 0.018

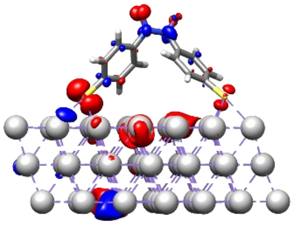

State 183
Energy: 3.059 eV
Osc.: 0.027

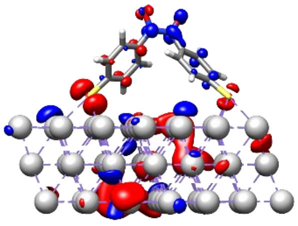

State 184
Energy: 3.065 eV
Osc.: 0.014

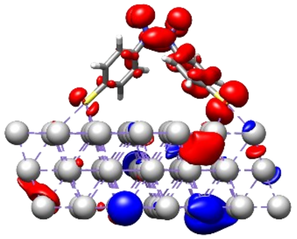

State 185
Energy: 3.069 eV
Osc.: 0.017

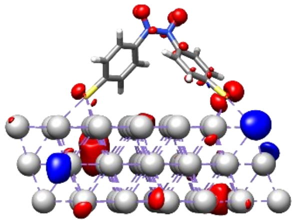

State 186
Energy: 3.082 eV
Osc.: 0.014

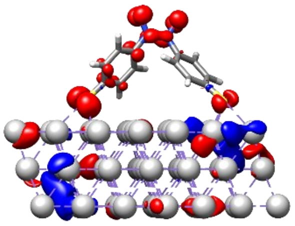

State 187
Energy: 3.093 eV
Osc.: 0.052

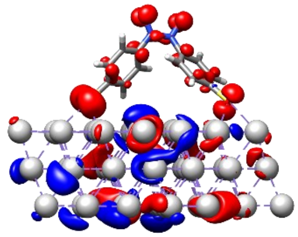

State 188
Energy: 3.098 eV
Osc.: 0.020

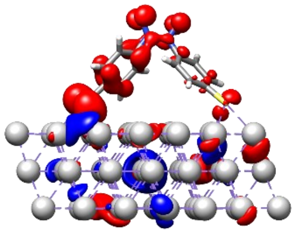

State 189
Energy: 3.109 eV
Osc.: 0.035

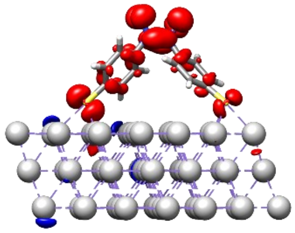

State 190
Energy: 3.114 eV
Osc.: 0.141

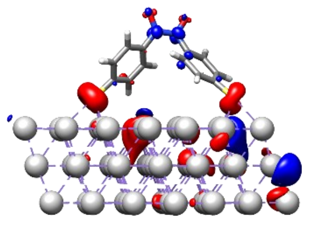

State 191
Energy: 3.123 eV
Osc.: 0.064

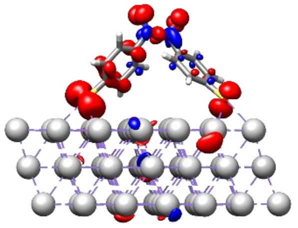

State 192
Energy: 3.126 eV
Osc.: 0.057

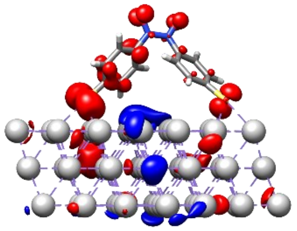

State 193
Energy: 3.135 eV
Osc.: 0.019

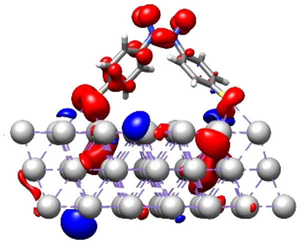

State 194
Energy: 3.141 eV
Osc.: 0.031

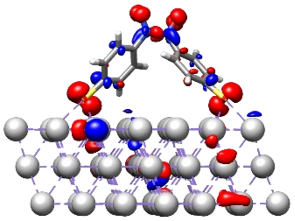

State 195
Energy: 3.143 eV
Osc.: 0.068

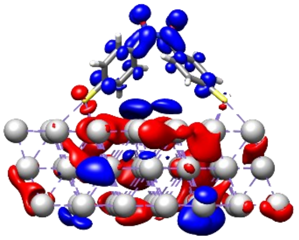

State 196
Energy: 3.149 eV
Osc.: 0.017

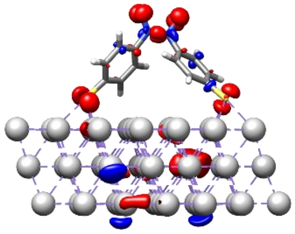

State 197
Energy: 3.162 eV
Osc.: 0.182

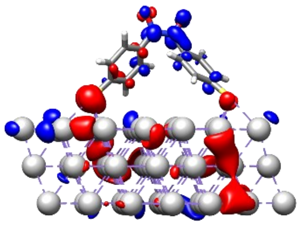

State 198
Energy: 3.172 eV
Osc.: 0.239

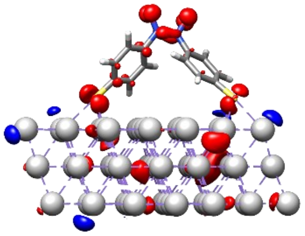

State 199
Energy: 3.185 eV
Osc.: 0.129

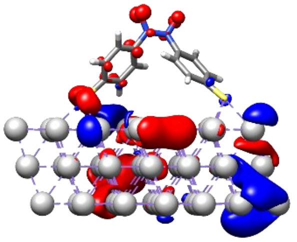

State 200
Energy: 3.187 eV
Osc.: 0.039

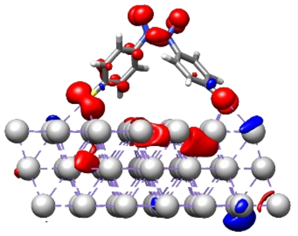

State 203
Energy: 3.205 eV
Osc.: 0.038

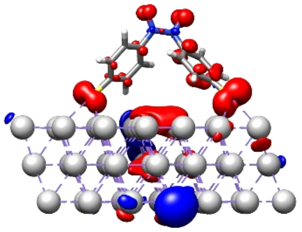

State 204
Energy: 3.212 eV
Osc.: 0.101

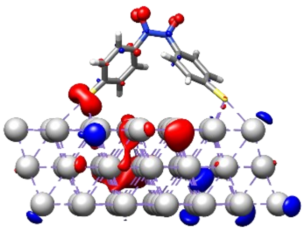

State 205
Energy: 3.223 eV
Osc.: 0.133

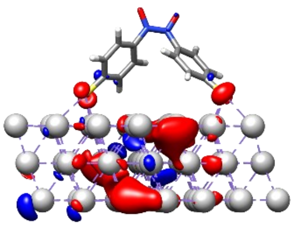

State 206
Energy: 3.232 eV
Osc.: 0.133

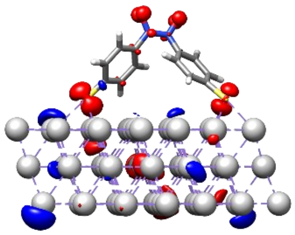

State 207
Energy: 3.238 eV
Osc.: 0.074

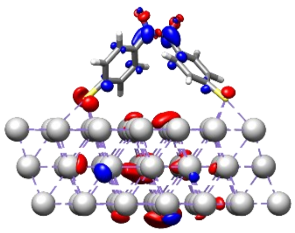

State 208
Energy: 3.242 eV
Osc.: 0.116

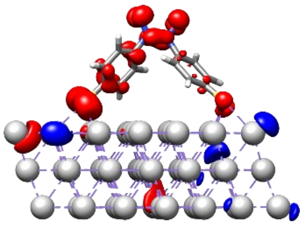<br>State 209<br>Energy: 3.260 eV<br>Osc.: 0.033	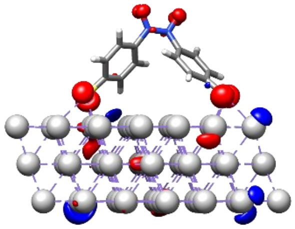<br>State 210<br>Energy: 3.260 eV<br>Osc.: 0.137	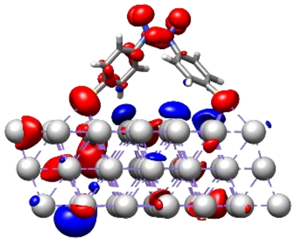<br>State 211<br>Energy: 3.266 eV<br>Osc.: 0.045	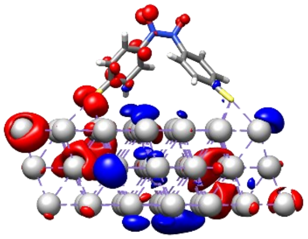<br>State 213<br>Energy: 3.276 eV<br>Osc.: 0.524
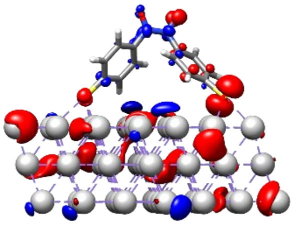<br>State 214<br>Energy: 3.279 eV<br>Osc.: 0.013	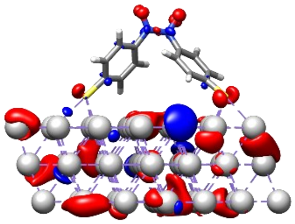<br>State 215<br>Energy: 3.291 eV<br>Osc.: 0.012	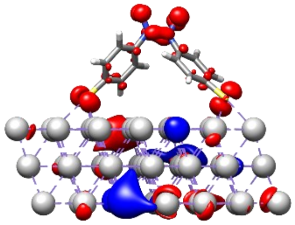<br>State 216<br>Energy: 3.296 eV<br>Osc.: 0.126	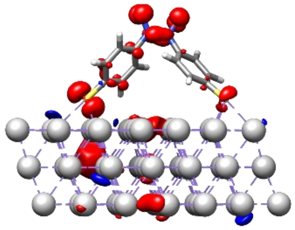<br>State 217<br>Energy: 3.300 eV<br>Osc.: 0.025
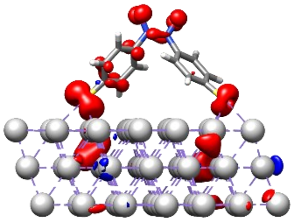<br>State 218<br>Energy: 3.305 eV<br>Osc.: 0.061	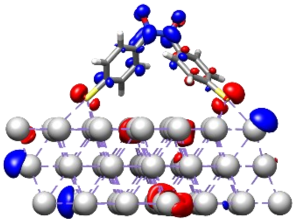<br>State 219<br>Energy: 3.312 eV<br>Osc.: 0.517	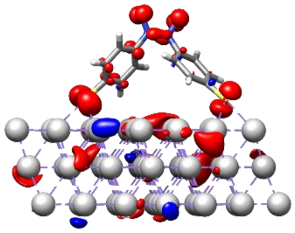<br>State 220<br>Energy: 3.321 eV<br>Osc.: 0.019	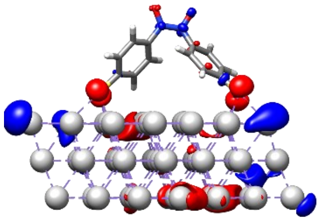<br>State 221<br>Energy: 3.329 eV<br>Osc.: 0.125
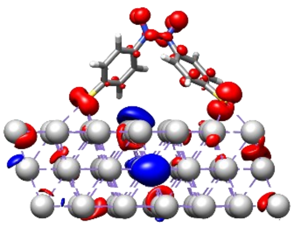<br>State 222<br>Energy: 3.330 eV<br>Osc.: 0.077	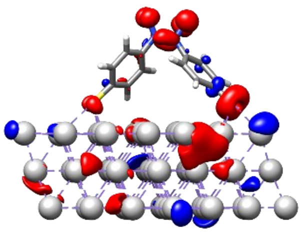<br>State 223<br>Energy: 3.336 eV<br>Osc.: 0.145		

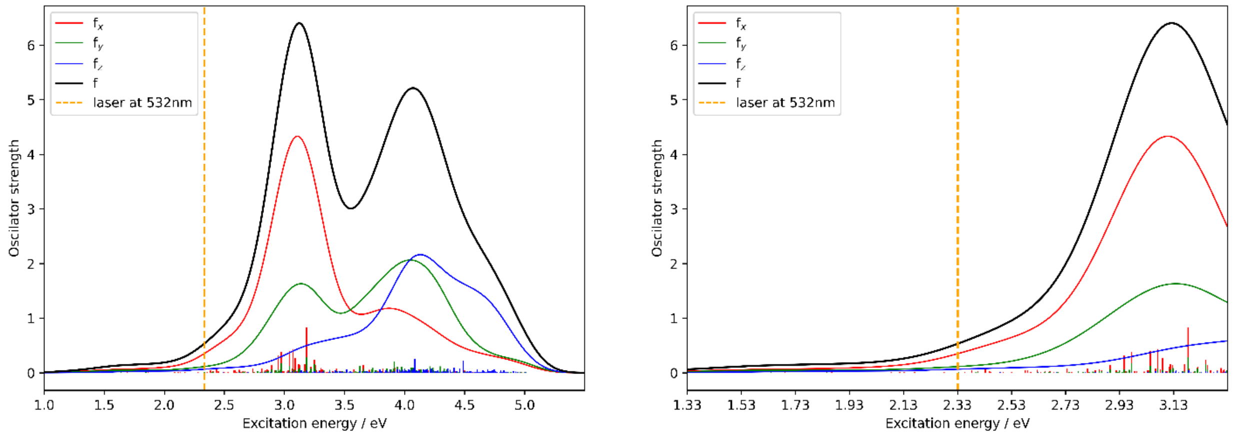

Figure S19: Electronic absorption spectrum of NTP (in black); *x*-, *y*-, and *z*-polarized spectra are given in red, green, and blue, respectively. The exaction energy of the irradiating laser (2.33 eV, 532 nm) is indicated (orange dashed line). All transitions were broadened by Gaussian functions with a full width at half maximum of 1500 cm^{-1}.

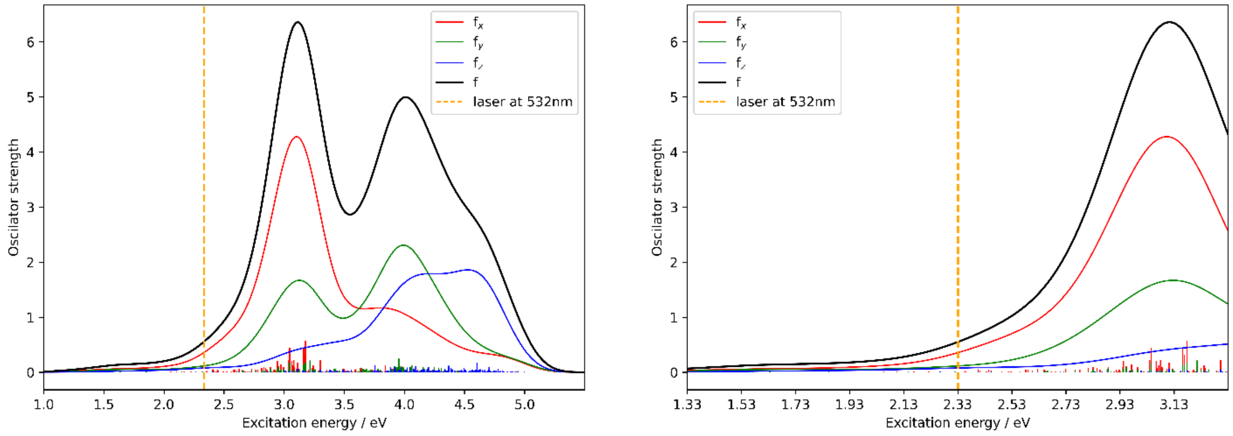

Figure S20: Electronic absorption spectrum of ATP (in black); *x*-, *y*-, and *z*-polarized spectra are given in red, green, and blue, respectively. The exaction energy of the irradiating laser (2.33 eV, 532 nm) is indicated (orange dashed line). All transitions were broadened by Gaussian functions with a full width at half maximum of 1500 cm^{-1}.

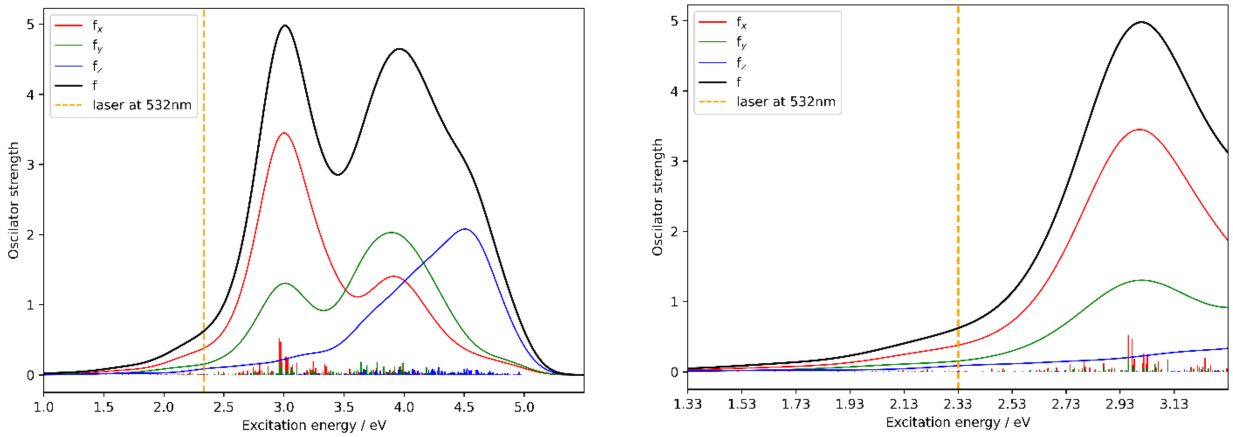

Figure S21: Electronic absorption spectrum of DMAB (in black); *x*-, *y*-, and *z*-polarized spectra are given in red, green, and blue, respectively. The exaction energy of the irradiating laser (2.33 eV, 532 nm) is indicated (orange dashed line). All transitions were broadened by Gaussian functions with a full width at half maximum of 1500 cm^{-1}.

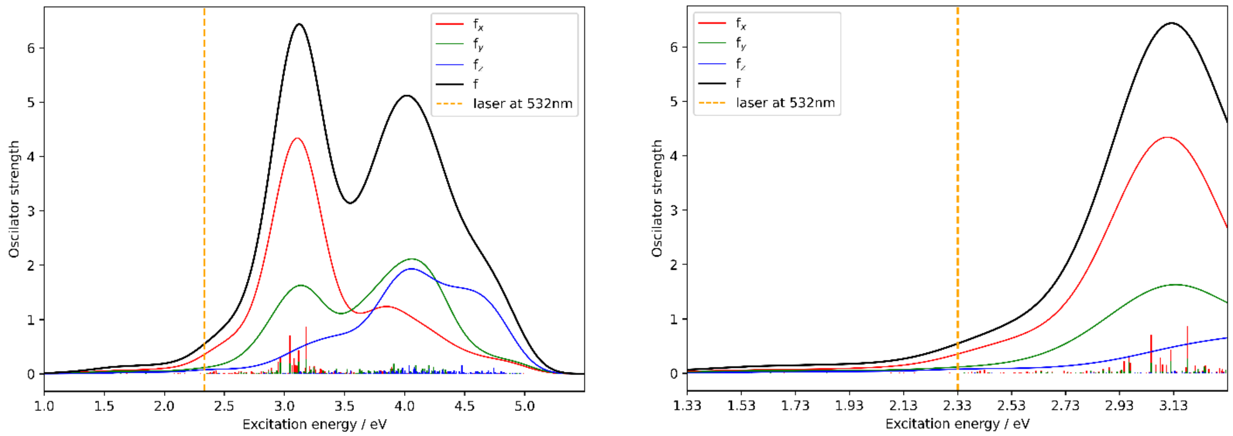

Figure S22: Electronic absorption spectrum of intermediate A (in black); *x*-, *y*-, and *z*-polarized spectra are given in red, green, and blue, respectively. The exaction energy of the irradiating laser (2.33 eV, 532 nm) is indicated (orange dashed line). All transitions were broadened by Gaussian functions with a full width at half maximum of 1500 cm^{-1}.

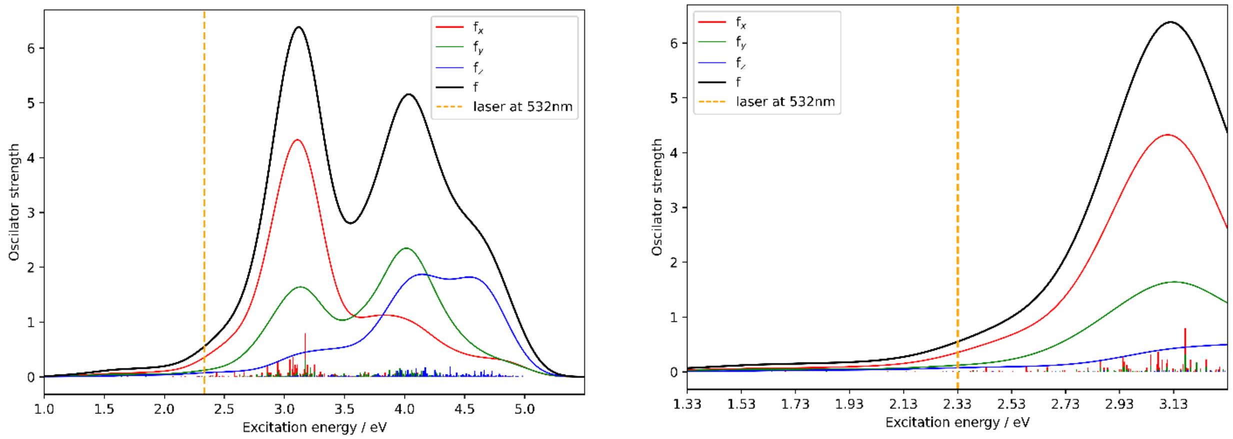

Figure S23: Electronic absorption spectrum of intermediate B (in black); *x*-, *y*-, and *z*-polarized spectra are given in red, green, and blue, respectively. The exaction energy of the irradiating laser (2.33 eV, 532 nm) is indicated (orange dashed line). All transitions were broadened by Gaussian functions with a full width at half maximum of 1500 cm^{-1}.

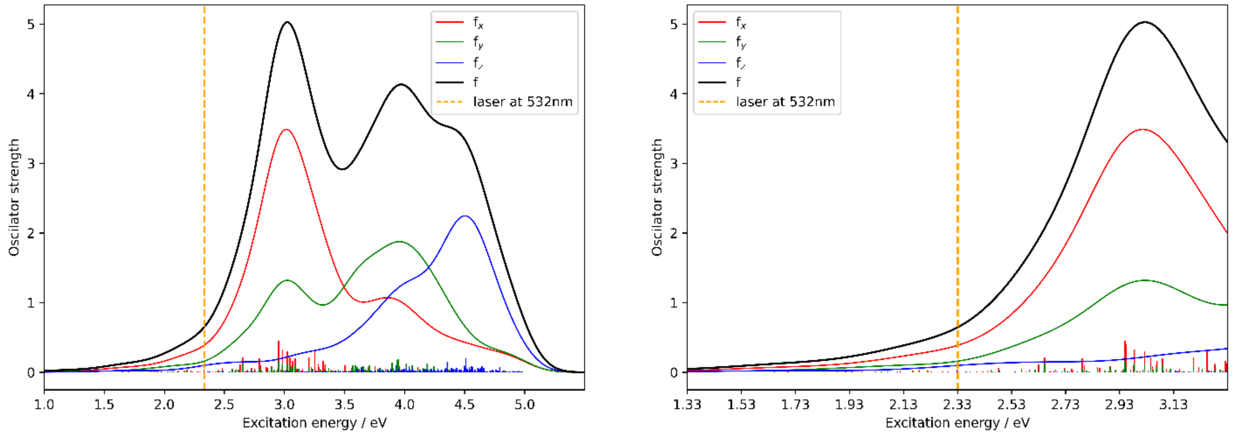

Figure S24: Electronic absorption spectrum of intermediate C (in black); *x*-, *y*-, and *z*-polarized spectra are given in red, green, and blue, respectively. The exaction energy of the irradiating laser (2.33 eV, 532 nm) is indicated (orange dashed line). All transitions were broadened by Gaussian functions with a full width at half maximum of 1500 cm^{-1}.

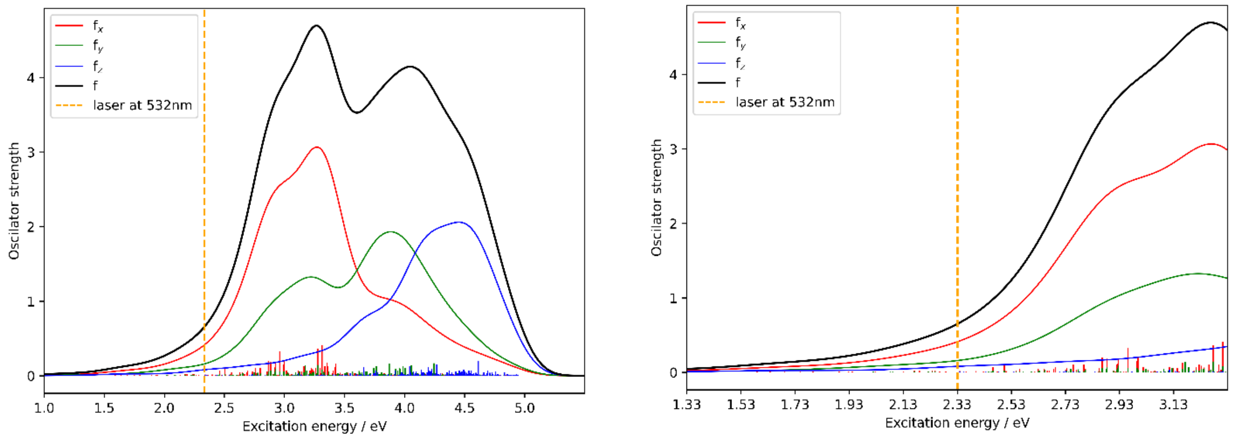

Figure S25: Electronic absorption spectrum of intermediate D (in black); *x*-, *y*-, and *z*-polarized spectra are given in red, green, and blue, respectively. The exaction energy of the irradiating laser (2.33 eV, 532 nm) is indicated (orange dashed line). All transitions were broadened by Gaussian functions with a full width at half maximum of 1500 cm^{-1}.
