# Supplementary material for: Mechanism of Plasmon-Induced Catalysis of Thiolates and the Impact of Reaction Conditions
Source: J Am Chem Soc. 2024 Jan 26;146(5):3031–42. doi: 10.1021/jacs.3c09309 (PMC10859934; doi:10.1021/jacs.3c09309)
Supplement: Supplementary file 2 — ja3c09309_si_002.pdf [file ja3c09309_si_002.pdf]

Supporting Information 1

Mechanism of Plasmon-Induced Catalysis of Thiolates and the Impact of Reaction Conditions

Xiaobin Yao[1,2], Sadaf Ehtesabi[2], Christiane Höppener[1,2], Tanja Deckert-Gaudig[1,2], Henrik Schneidewind[1], Stephan Kupfer[2], Stefanie Gräfe[2,3], Volker Deckert[1,2]

1. Leibniz Institute of Photonic Technology (IPHT), Albert-Einstein-Str. 9, 07745 Jena, Germany

2. Institute of Physical Chemistry (IPC) and Abbe Center of Photonics, Friedrich Schiller University Jena, Helmholtzweg 4, 07743 Jena, Germany

3. Fraunhofer Institute of Applied Optics and Precision Engineering, Albert-Einstein-Str. 7, 07745 Jena, Germany

Corresponding author: volker.deckert@leibniz-ipht.de

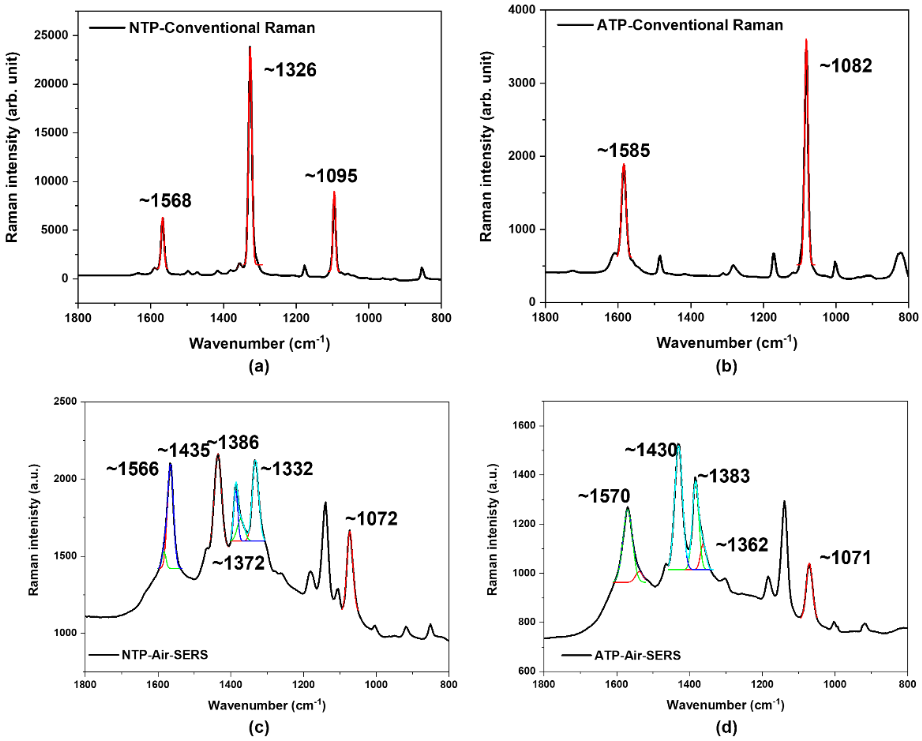

Figure S1. (a, b) Conventional Raman spectra of ATP and NTP and corresponding Gauss fitting on selected bands. Two bands at ~1082 cm^{-1} and ~1585 cm^{-1} are used as the marker bands of ATP while three bands at ~1095 cm^{-1}, ~1326 cm^{-1} and ~1568 cm^{-1} are used as marker bands of NTP. λ= 532 nm, ~28 mW/1 s for ATP spectrum and ~3.7 mW/0.5 s for NTP spectrum, 100 acc. (c, d) SERS spectra of ATP- and NTP- SAMs on Ag island film investigated under ambient conditions and corresponding Gauss fitting on selected bands. Fitting of DMAB bands from NTP (c) and ATP (d) indicates there is a band ~1360-1370 cm^{-1} hiding in the spectra. λ= 532 nm, P= ~1.50 mW, t_{acq} = 1 s, 100 acc. In all Gauss fitting processes, the second derivative was used to search possible hidden peaks, especially to the functional group regions of NO_2, N=O and N=N. For the aromatic ring breathing/C-S stretching the fitting range was set to ~1040-1120 cm^{-1}. For the NO_2/N=O group the fitting range was set to ~1270-1410 cm^{-1}. In contrast, for the azo (N=N) group from NTP and ATP samples the fitting range were set to ~1260-1490 cm^{-1} and ~1330-1460 cm^{-1}, respectively. For the aromatic ring stretching the fitting range was set to ~1510-1640 cm^{-1}. With reaching the largest R^2 value it was regarded as a successful fitting. These parameters were also applied to the following data treatments.

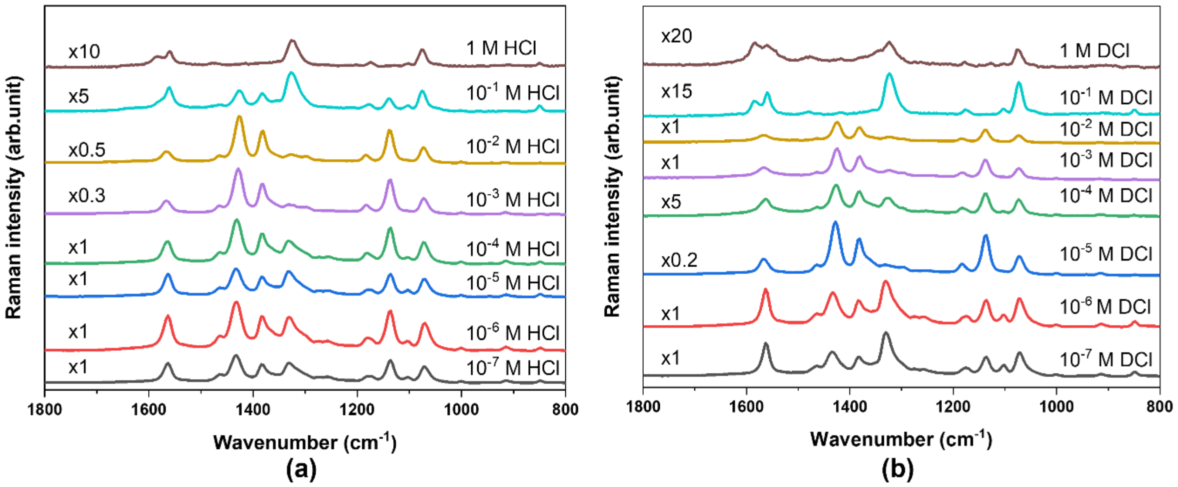

Figure S2. Concentration dependent SERS spectra of HCl_{aq} (a) and DCl_{aq} (b) treated NTP-SAMs on SERS substrates. Clearly, bands from azo group (N=N) of DMAB dominate the spectra from the concentration of 10^{-7} M to 10^{-2} M (*i.e.*, pH 2-7) in both HCl_{aq} and DCl_{aq}. When the concentrations of HCl_{aq} and DCl_{aq} are higher than 10^{-1} M, especially 1 M (*i.e.*, pH 0), NO_2 band dominates in the spectra. λ= 532 nm, P= ~260 µW, t_{acq} = 1 s, 20 acc.

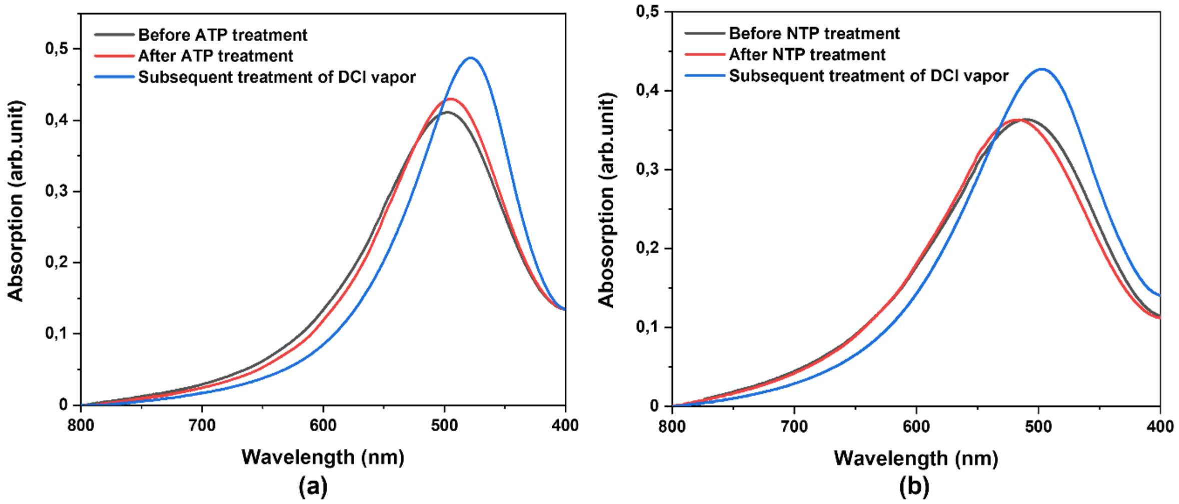

Figure S3. UV-Vis spectra of neat SERS substrates and after coating with an ATP- (a) or NTP- (b) SAMs before and after DCl vapor treatment. A cleaned glass slide was used for background correction. Average time: 0.1 s, data interval: 0.5 nm.

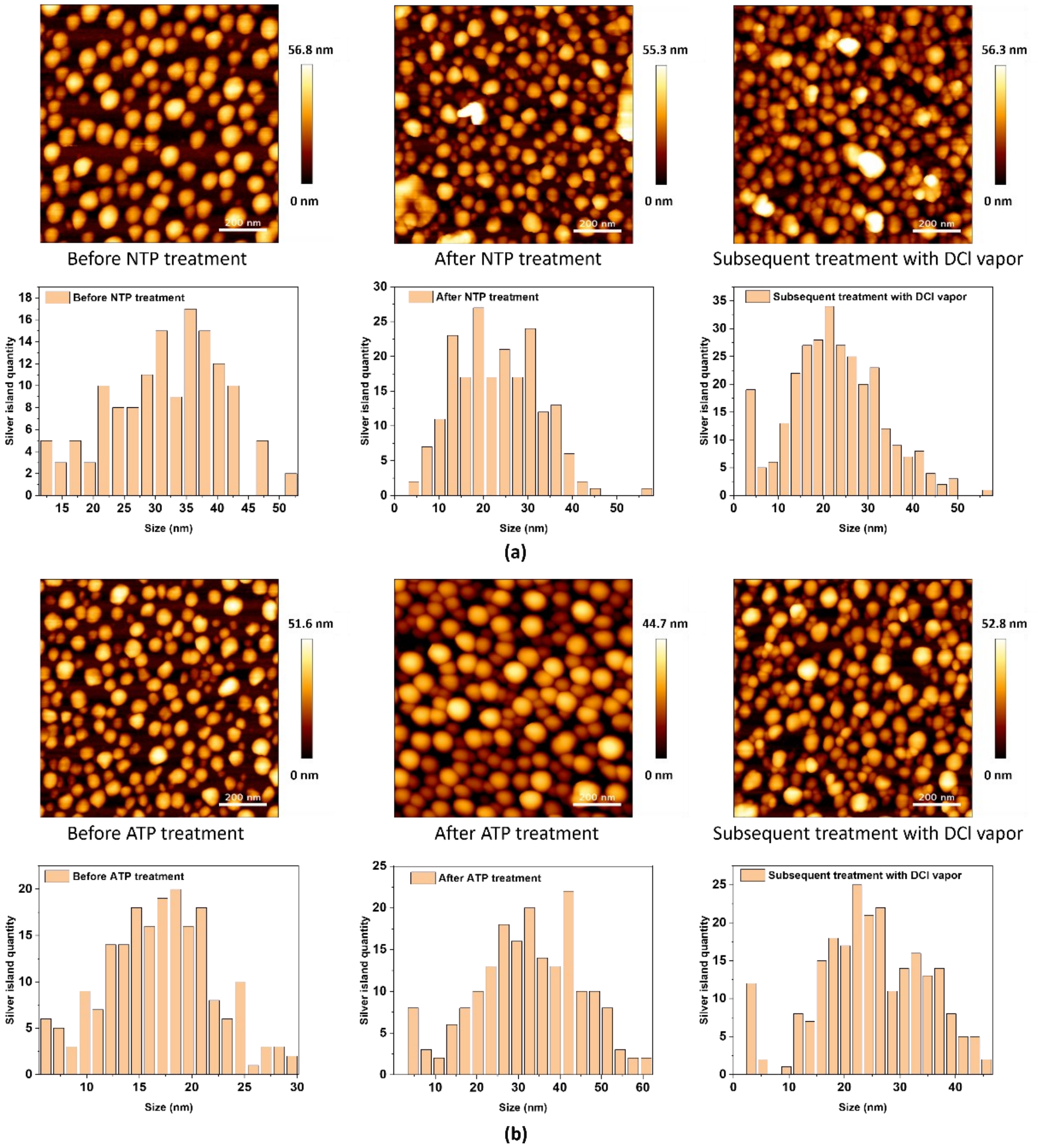

Figure S4. AFM topography of SERS substrates before and after coating with NTP (a) or ATP (b) SAMs and subsequent treatment with DCl vapor. Before and after coating with NTP or ATP SAMs, the silver islands had well-defined and regular spherical shapes. Smaller and more irregular structures were observed on the samples after treatment with DCl vapor.

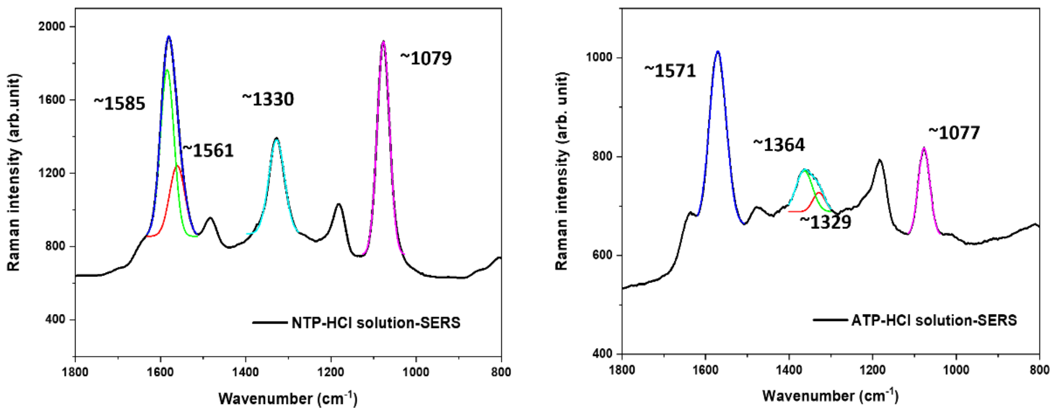

(a) (b)

Figure S5. Gauss fitting of selected bands of SERS spectra of NTP (a) and ATP (b). The spectral fitting of NTP sample in HCl solution indicates there is a band at ~1580 cm^{-1}. In contrast, the spectral fitting of ATP sample in HCl solution shows the existence of a yet unknown band at ~1360 cm^{-1}.

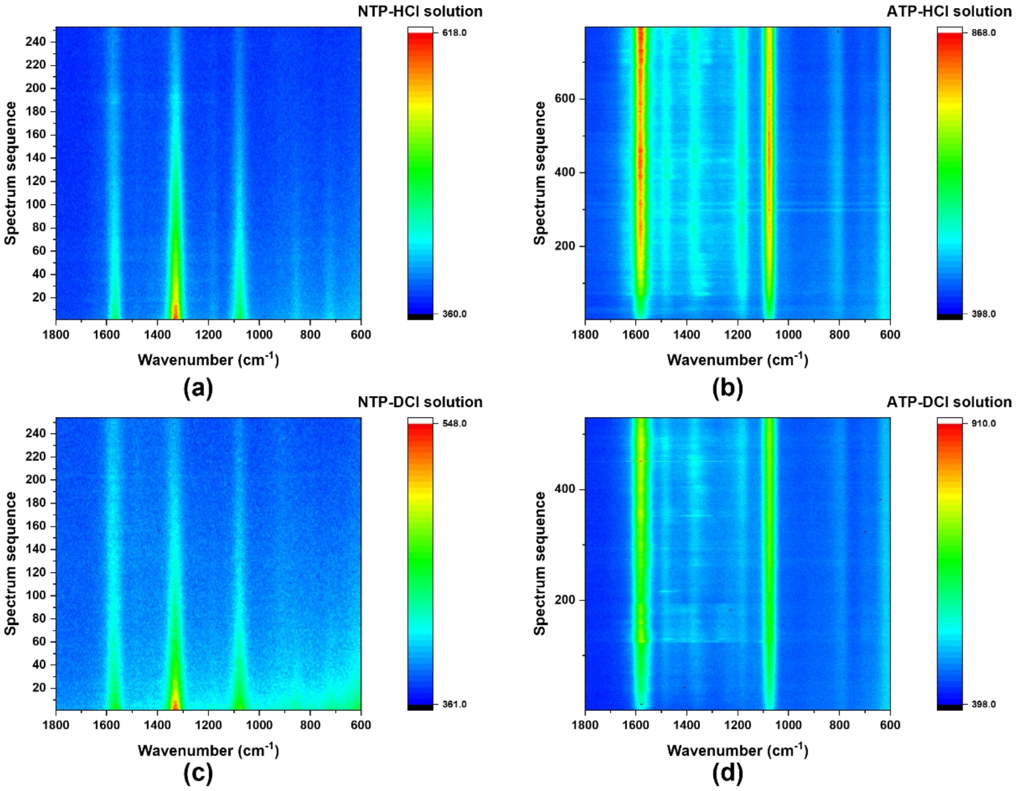

Figure S6. Contour images of time dependent measurements of NTP (a, c) and ATP (b, d) in HCl and DCl solutions, respectively. Laser wavelength: 532 nm, power: ~280 μW, acquisition time: 0.5 s/spectrum.

Table S1. Band information and assignments of NTP- and ATP- SAMs in HCl and DCl solutions from Figure 1, Figure S6 and other measurements. All bands are fitted by Origin Pro 2020b with R^2 values up to 99.9%. To NTP: The NO_2 mode was stable in HCl solution while it shifted in DCl solution. Ring stretching mode showed blue-shift in both HCl and DCl solutions. Some of them can be fitted to a band at ~1588 cm^{-1}. C-S/ring stretching mode was stable in both HCl and DCl solutions. To ATP: the new band (with further experiments, it is assigned to the N=O mode) was stable in HCl solution locating at ~1370 cm^{-1} while it shifted from ~1360 to ~1370 cm^{-1} in DCl solution. Ring stretching mode was stable in HCl solution while some of them can be fitted to a band at ~1567 cm^{-1} in DCl solution. C-S/ring breathing mode was relatively stable in both HCl and DCl solutions.

	**1M DCl solution**						**1M HCl solution**					
	Wavenumber (cm^{-1})											
**NTP**	Pos1 (Figure 1d)		Pos2		Pos3		Pos1’ (Figure 1b)		Pos2’		Pos3’	
Assignments	first	final	first	final	first	final	first	final	first	final	first	final
Ring stretching	**1569**	**1568/1589**	**1568**	**1572**	**1569**	**1562/1587**	**1567**	**1575**	**1566**	**1578**	**1565**	**1576**
**NO_2**	**1330**	**1339**	**1330**	**1334**	**1330**	**1337**	1329	1330	1329	1327	1328	1330
C-S/ring breathing	1078	1080	1078	1079	1079	1079	1078	1080	1077	1078	1077	1079
**ATP**	Pos1		Pos2 (Figure 1e)		Pos3		Pos1’ (Figure 1c)		Pos2’		Pos3’	
Assignments	first	final	first	final	first	final	first	final	first	final	first	final
Ring stretching	1566/1588	1582	1578	1568/1586	1582	1579	1581	1580	1579	1579	1580	1580
**N=O**	**1358**	**1370**	**1359**	**1369**	**1361**	**1366**	**1369**	**1370**	**1372**	**1368**	**1368**	**1372**
C-S/ring breathing	1075	1079	1075	1076	1075	1081	1076	1077	1076	1077	1076	1082

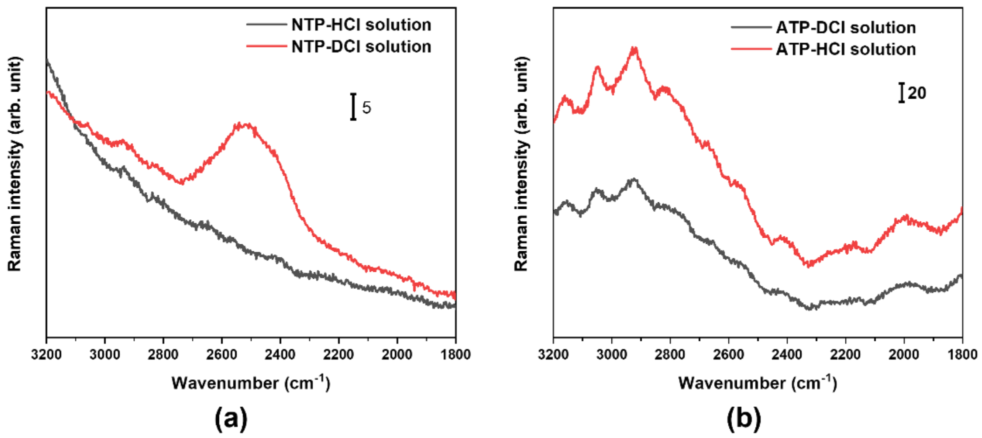

Figure S7. SERS spectra of NTP (a) and ATP (b) treated with HCl and DCl solutions. λ= 532 nm, P= ~ 280 µW, t_{acq} = 1 s, 50 acc.

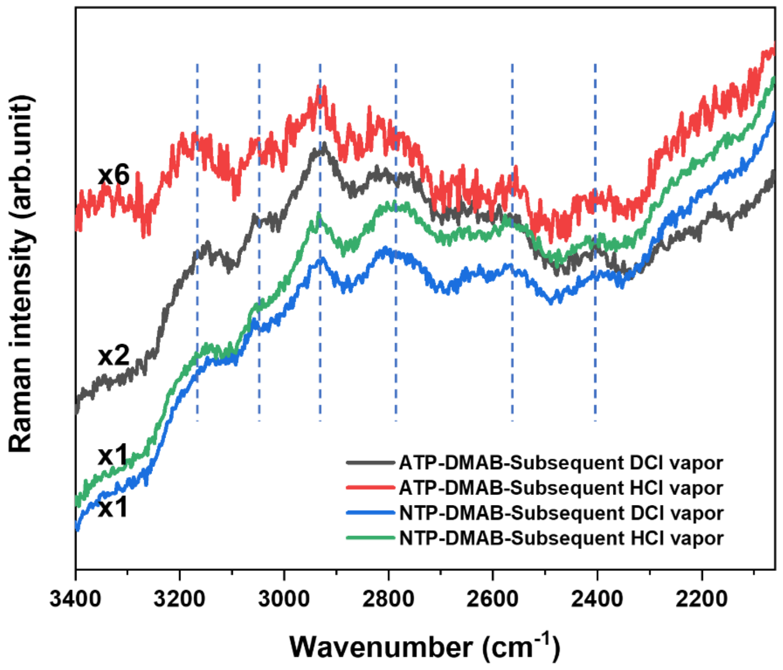

Figure S8. SERS spectra of ATP- and NTP- SAMs treated with DCl and HCl vapors. In the highly similar spectra no O-D, N-D or C-D were detected. ATP spectra were acquired with a power of ~570 μW while the NTP spectra were recorded at ~280 μW. Spectra were averaged from 40 spectra with an acquisition time of 0.5 s/spectrum.

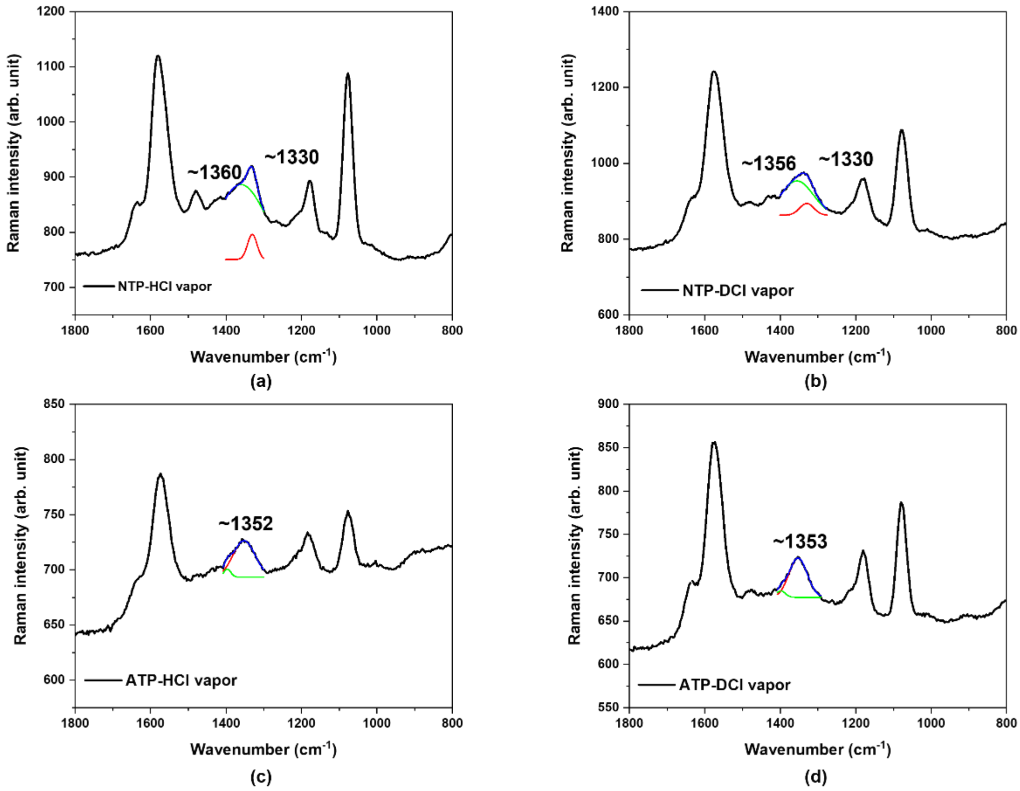

Figure S9. Gauss fitting of selected bands of SERS spectra from Figure 2. A ~1355 cm^{-1} band is fitted from NTP (a, b) and ATP (c, d) spectra in both HCl and DCl vapors. The C-S/ring breathing and ring stretching modes are extracted by peak analyzer of Origin Pro 2020b. Concerned of the merging bands, the N=O mode is extracted by Gauss fitting of Origin Pro 2020b. The differences among these bands acquired from acidic vapors are much smaller when compared to the results acquired from acidic solutions in Table S1, which indicates the influence of H_2O and D_2O.

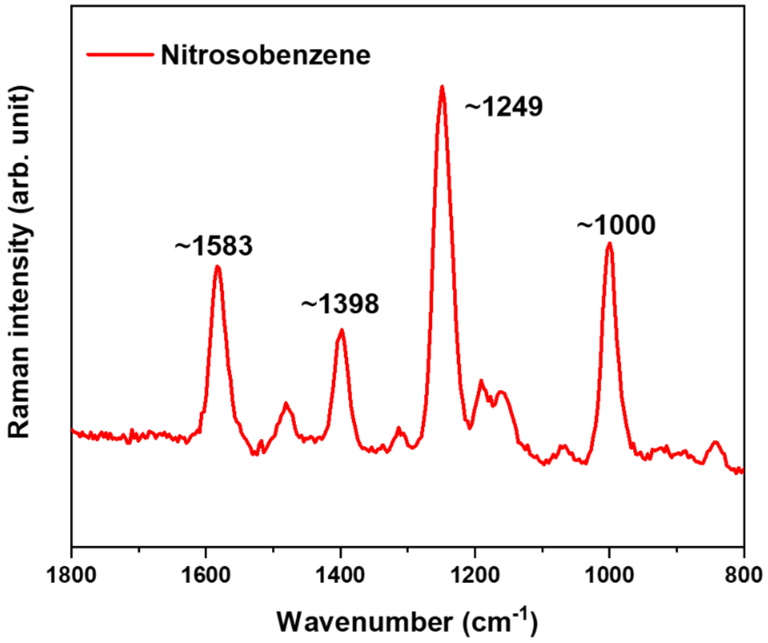

Figure S10. Conventional Raman spectrum of nitrosobenzene. The band at ~1398 cm^{-1} is assigned to N=O group. The bands at ~1583cm^{-1}, ~1249cm^{-1} and ~1000cm^{-1} are assigned to ring stretching, C-N and ring breathing modes, respectively. Nitrosobenzene was purchased from Sigma-Aldrich, Cat. No.: N24609. Laser wavelength: 532 nm, laser power: ~80 μW, acquisition time: 5 s, accumulation: 2.

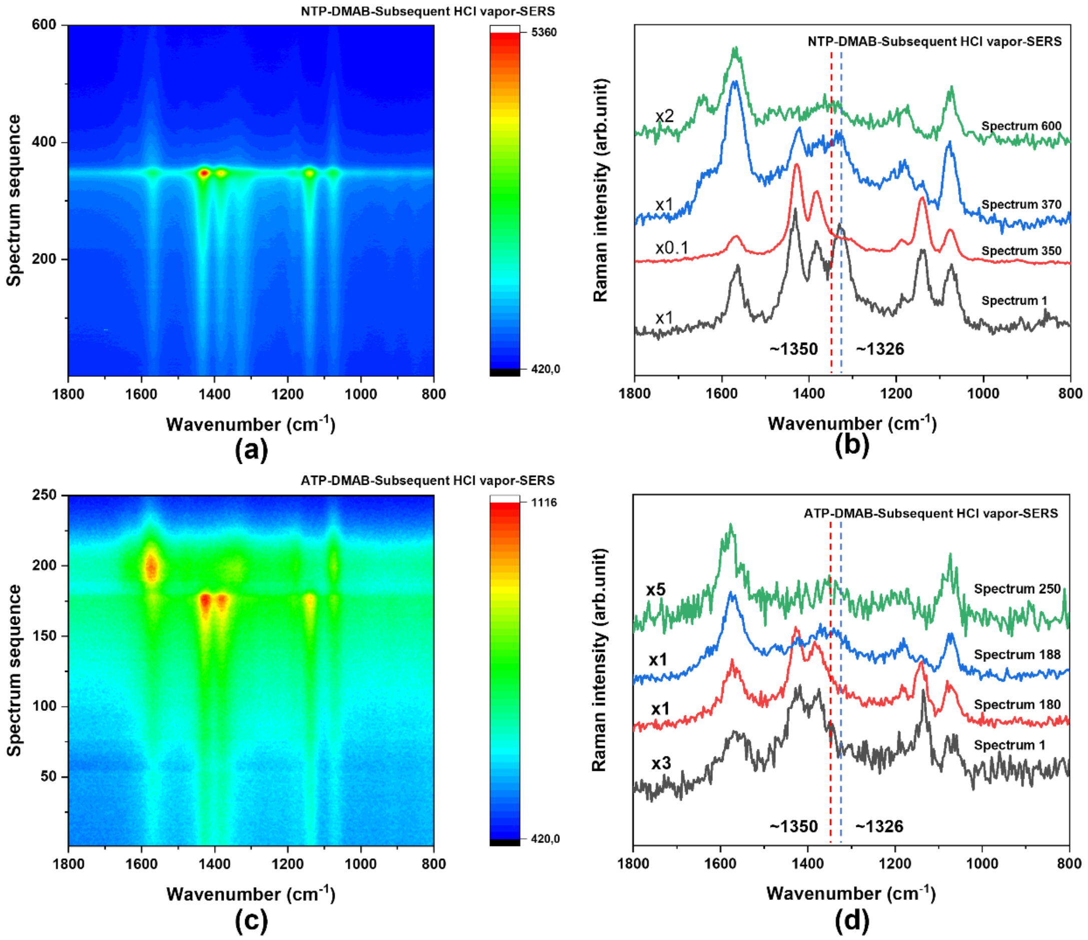

Figure S11. Time-dependent SERS spectra of NTP- (a) and ATP- (c) SAMs subsequently treated with HCl vapor. In the selected spectra (b, d), the conversion from DMAB to TP* is clearly visible. The red dash lines highlight the NO mode and the blue lines highlight the ring stretching and NO_2 modes. Laser wavelength: 532 nm, power: ~280 μW, acquisition time: 0.5 s/spectrum.

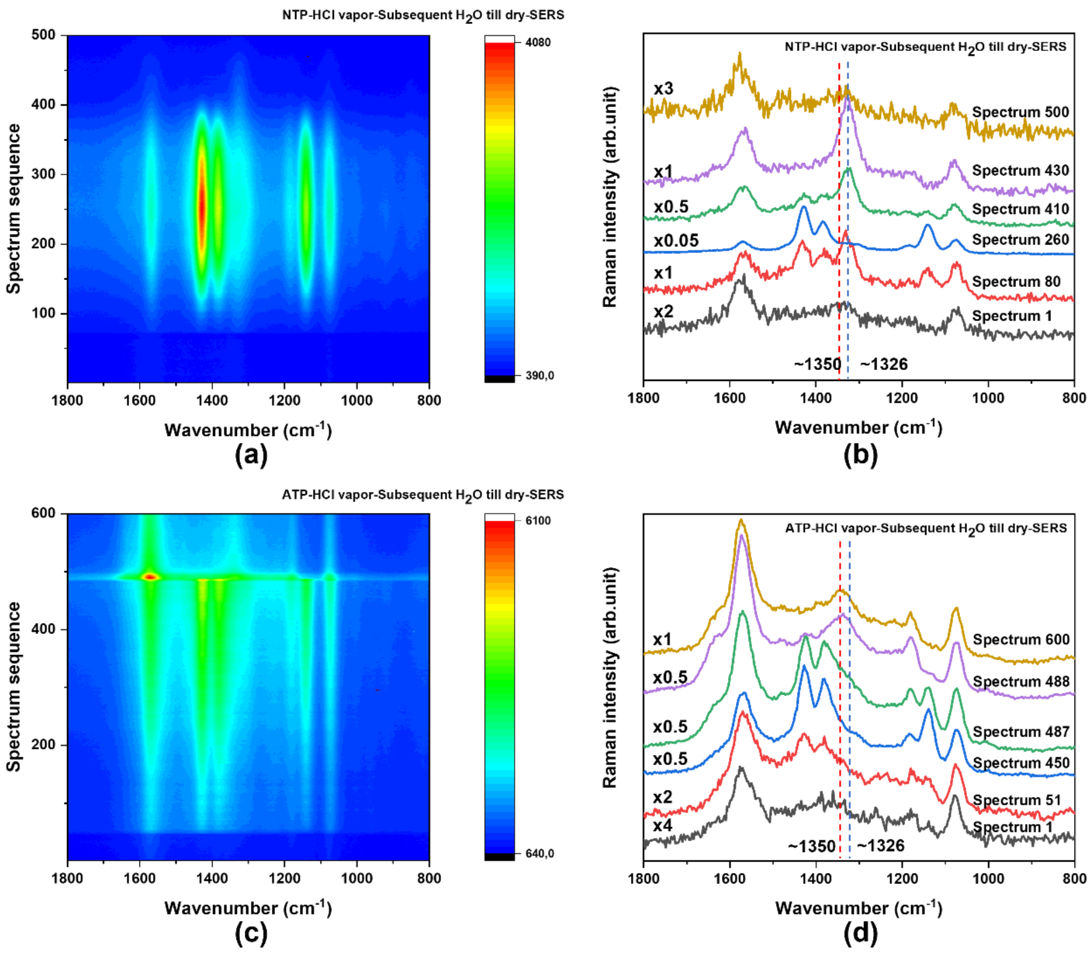

Figure S12. Time-dependent SERS spectra of NTP- (a) and ATP- (c) SAMs subjected to HCl vapor and subsequently treated with H_2O. In the selected spectra, the conversion from TP* to DMAB via NTP (b) and ATP (d) is evident. Laser wavelength: 532 nm, power: ~280 µW, acquisition time: 0.5 s/spectrum.

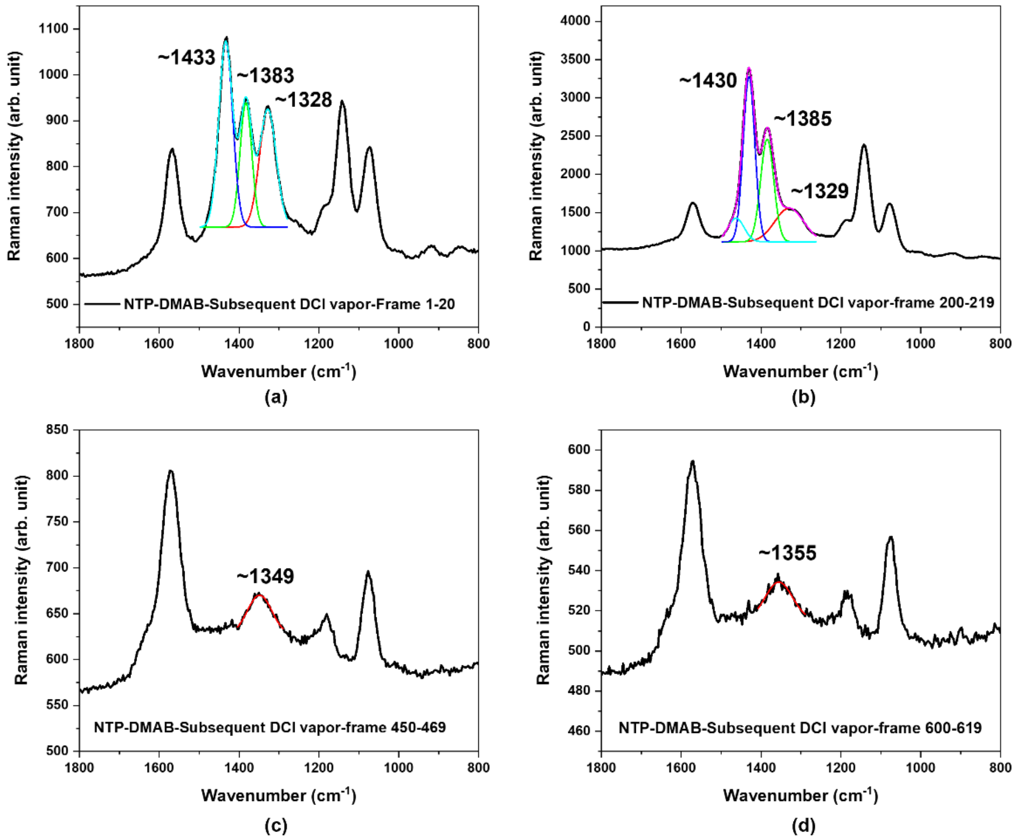

Figure S13. Gauss fitting of selected bands of averaged SERS spectra of NTP-DMAB-subsequent DCl vapor from Figure 5b. Fitted spectra were averaged from 20 continuous spectra. (a) When NTP-SAM samples were illuminated by laser, both NTP and DMAB existed. (b) After that, NO_2 band decreased while DMAB still increased. (c) Once DCl vapor covered on the samples, the DMAB bands quickly disappeared while TP* formed. (d) At the end of measurement, N=O band dominated and located at ~1355 cm^{-1}.

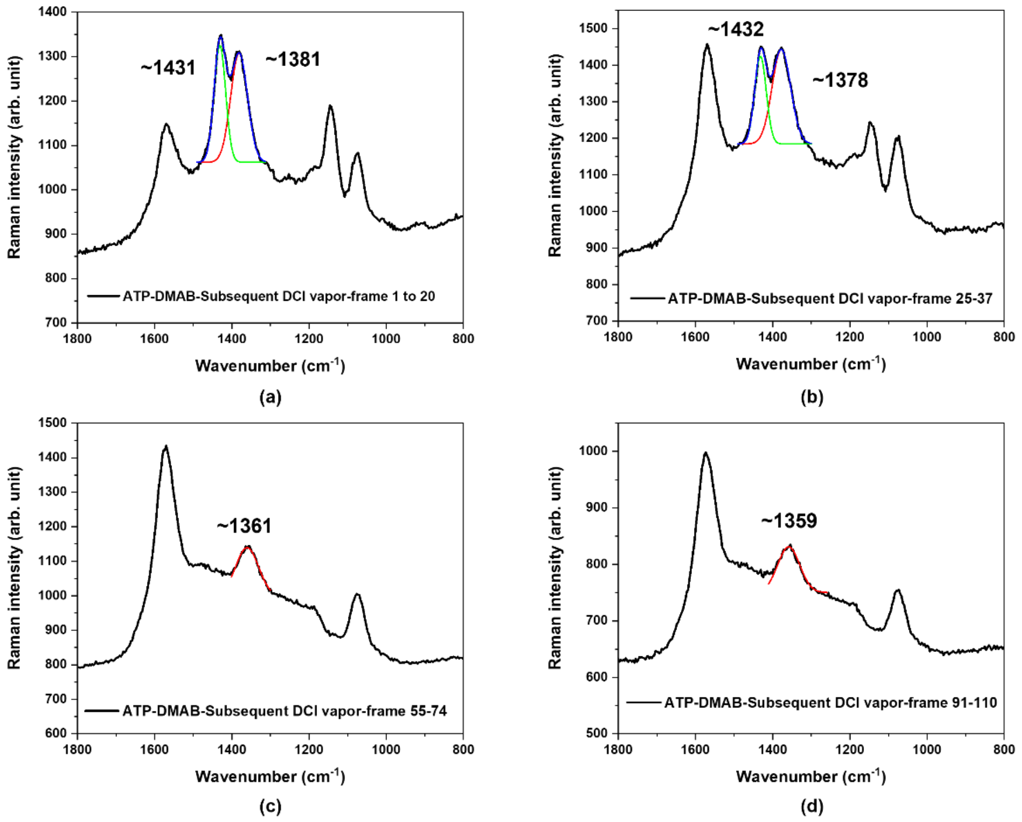

Figure S14. Gauss fitting of selected bands of averaged SERS spectra of ATP-DMAB-subsequent DCl vapor from Figure 5d. Fitted spectra were averaged from 13 to 20 continuous spectra. (a) When ATP-SAM samples were illuminated, DMAB quickly formed. (b) With the influence of DCl vapor, the intensity of the band at ~1430 cm^{-1} clearly decreased. (c-d) Once DCl vapor covered on the samples, TP* dominated and located at ~1360 cm^{-1}.

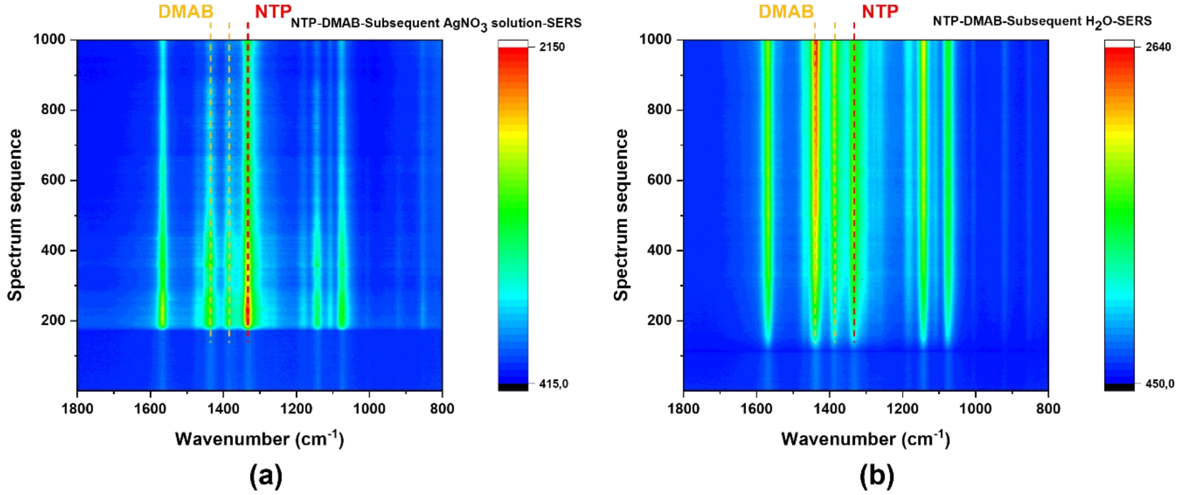

Figure S15 Time-dependent measurements of NTP-SAMs on SERS substrates treated by $1.1x10^{-4}$ M $AgNO_3$ solution (a) and H_2O (b), respectively. With the treatment of $AgNO_3$ solution, DMABs were oxidized to NTPs and this effect was kept until the substrate was dry again which indicates the suppression of Ag^+ on the reduction of NTP. In contrast, there is no clear change on the time-dependent measurement of NTP treated by H_2O in which DMAB still dominated in the entire measurement. Laser wavelength: 532 nm, power: ~280 μW, time acquisition: 0.5 s.

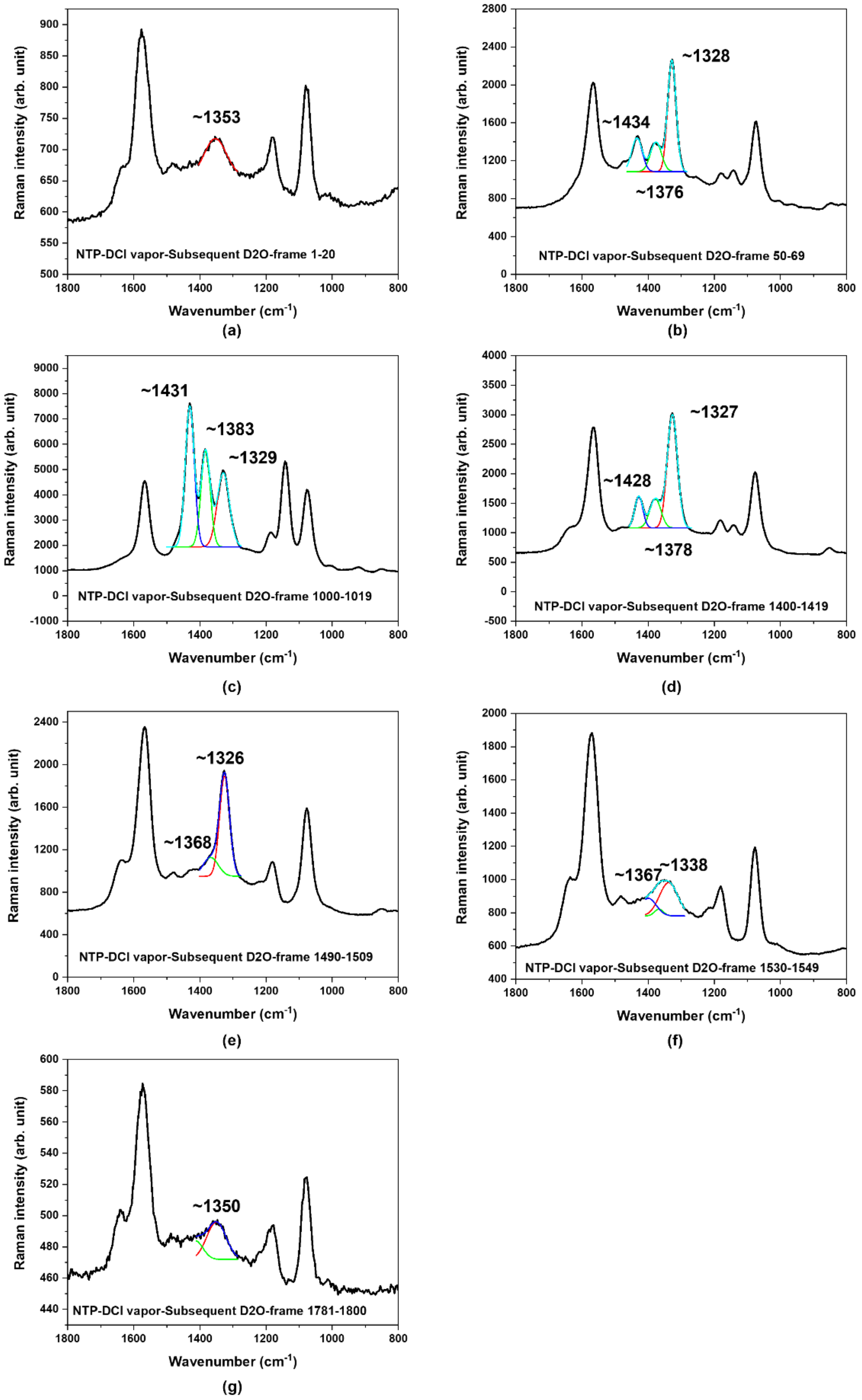

Figure S16. Gauss fitting of averaged SERS spectra of NTP-DCl vapor-subsequent D_2O from Figure 6b. Fitted spectra were averaged from 20 continuous spectra. (a) When DCl vapor covered on the

samples, a band is fitted at 1353 cm^{-1} from the averaged spectrum which is assigned to the N=O stretching of TP*. (b) Once D_2O was added, both DMAB and NTP bands were observed while NTP dominated. (c) After that, NTP was dimerized into DMAB which dominated the following spectra. (d) With the evaporation of D_2O, DMAB bands decreased. (e) At some point of the evaporation, NTP dominated the spectra. (f) With DCl concentration increased, the peak shifted towards ~1350 cm^{-1}. (g) At the end of measurement, TP* dominated the spectra again which is fitted at 1350 cm^{-1}.

1. $\mathrm{Ag} \xrightarrow{h\upsilon\ (\mathrm{DCl/HCl})} \mathrm{Ag}^* + \mathrm{e}^- + \mathrm{h}^+$ (Hot carriers from plasmon decay.)

$\mathrm{NTP} \xrightarrow{\mathrm{H}^+/\mathrm{e}^-} \mathrm{TP}^*$ (in DCl/HCl vapor)

2. $\mathrm{TP}^* \xrightarrow{\mathrm{h}^+} \mathrm{NTP}$ (injection of D_2O/H_2O increases the pH and triggers the oxidation of TP* to NTP)

$\mathrm{NTP/TP}^* \xrightarrow{\mathrm{e}^-} \mathrm{DMAB}$ (Subsequent reduction of NTP and remaining TP* react via the DMAB pathway)

3. $\mathrm{DMAB} \xrightarrow{\mathrm{h}^+} \mathrm{NTP}, \mathrm{NTP} \xrightarrow{\mathrm{H}^+/\mathrm{e}^-} \mathrm{TP}^*$(With the evaporation of water, DCl/HCl dominates the surface again and the monomer pathway is favorable.)

$\mathrm{DMAB} \xrightarrow{\mathrm{H}^+/\mathrm{e}^-} \mathrm{TP}^*$(see Route 1 - a direct dissociation is also possible.)

Scheme S1. Proposed reaction steps of Route 3 . First, NTP is reduced to TP* in DCl/HCl vapors. Second, With D_2O/H_2O treatment, TP* is oxidized to NTP and is then dimerized to DMAB. According to the results in Figure S15, it is supposed that partial Ag^+ ions from the interaction of DCl/HCl and Ag in water deplete electrons. Third, with the evaporation of water, the pH value increases. DMAB is dissociated to NTP first and is then reduced to TP* again.

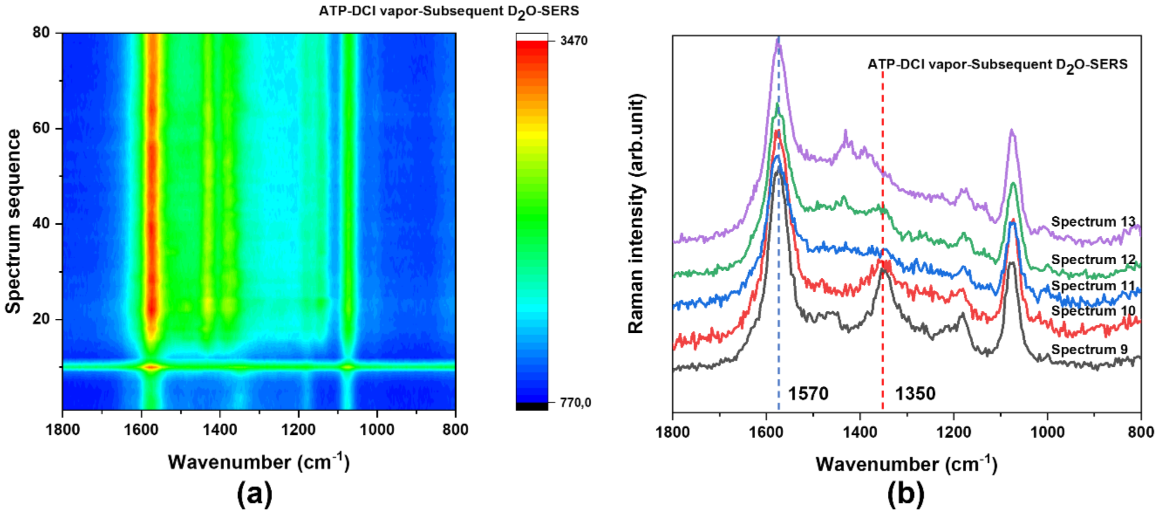

Figure S17. Time-dependent SERS spectra of an ATP-SAM treated with DCl vapor and subsequently treated with D_2O. During the conversion from TP* to DMAB, spectrum 11 shows a decrease of the band at ~1340-1350 cm^{-1} which indicates the suspicious formation of ATP prior the dimerization to DMAB. Laser wavelength: 532 nm, power: ~280 µW, acquisition time: 0.5 s/spectrum.

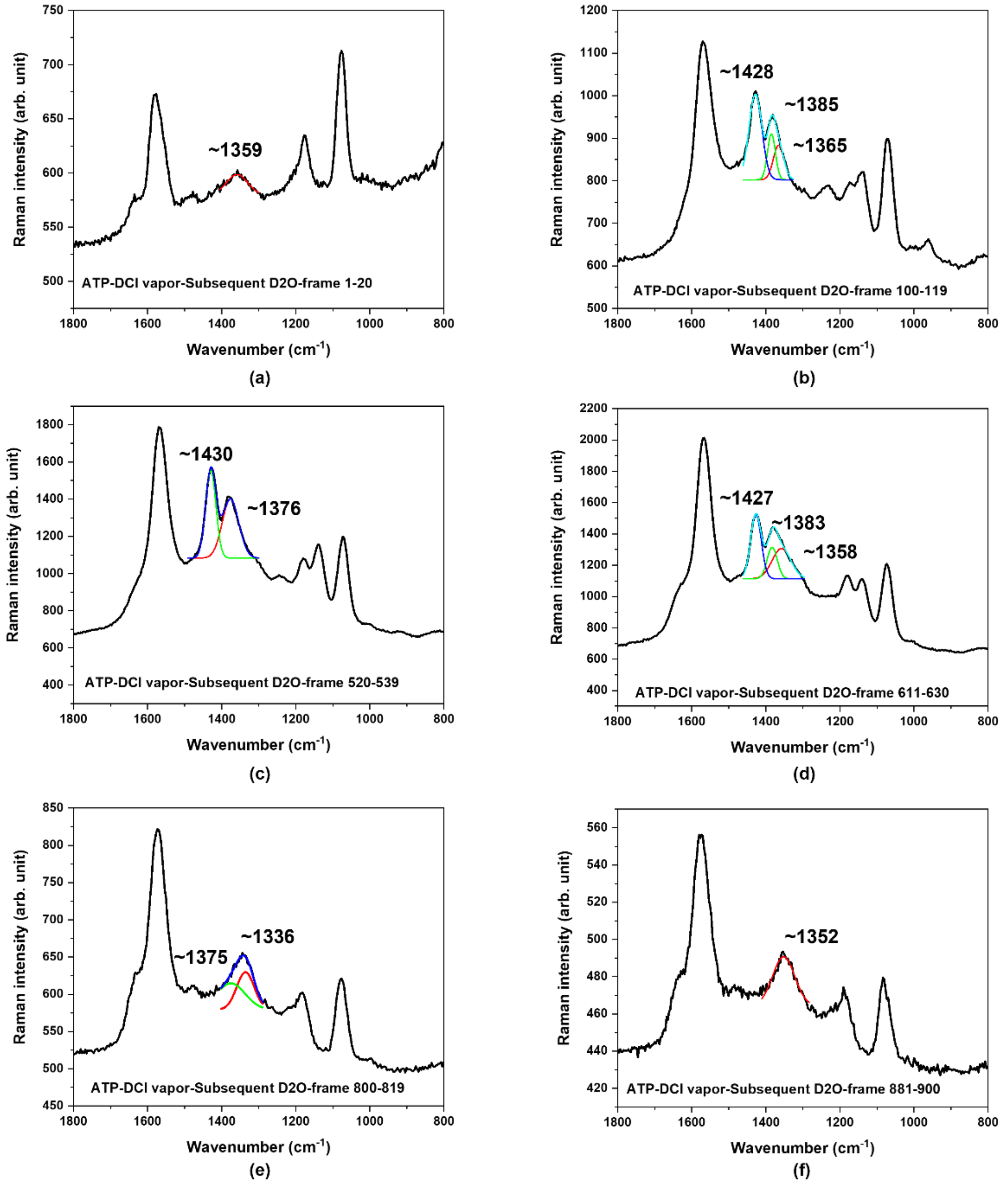

Figure S18. Gauss fitting of averaged spectra of ATP-DCl vapor-subsequent D_2O from Figure 6d. (a) When ATP was treated by DCl vapor, a band is fitted at 1359 cm^{-1} from the average spectrum which is assigned to the N=O stretching of TP*. (b-c) After D_2O treatment, DMAB bands could be fitted. (d) With the evaporation of D_2O, a band at 1358 cm^{-1} could be fitted to TP*. (e) With further evaporation, the band shifted toward ~1350 cm^{-1} which can be fitted into N=O and NO_2 bands indicating that may be a trace amount of NTP formed in this process. (f) Once the sample was dried, the N=O band of TP* dominated the spectrum which is fitted at 1352 cm^{-1}.

1. $Ag \xrightarrow{h\upsilon\ (DCl/HCl)} Ag^{*} + e^{-} + h^{+}$ (Hot carriers originated from plasmon decay.)

$ATP \xrightarrow{H^{+}/h^{+}} TP^{*}$ (in DCl/HCl vapor.)

2. $TP^{*} \xrightarrow{h^{+}} DMAB$ (The injection of D_2O/H_2O increased the pH value. A direct dimerization of TP* into DMAB induced by holes.)

$TP^{*} \xrightarrow{e^{-}} ATP \xrightarrow{O_2/h^{+}} DMAB$ (Parts of TP* may be reduced to ATP followed by dimerization to DMAB.)

3. $DMAB \xrightarrow{H^{+}/h^{+}} TP^{*}$ (the evaporation of D_2O/H_2O, referred to Route 2, possibly leads to a direct dissociation)

$DMAB^{*} \xrightarrow{h^{+}} NTP \xrightarrow{e^{-}} TP^{*}$ (A weak band can be fitted to the NO_2 group of NTP which indicates this route.)

Scheme S2. Proposed reaction steps in Route 4. First, ATP is oxidized to TP* in DCl/HCl vapors. Second, With D_2O/H_2O treatment, TP* is dimerized to DMAB. The route via ATP (TP*→ATP→DMAB) cannot be excluded due to the lack of unique marker band of ATP. Third, with the evaporation of water, the pH value increases. DMAB is dissociated to TP* again. In this process, a weak band can be fitted to NO_2 means a route via NTP to TP* may be possible but is energetic unfavorable.
